# SNP mining in *C. clementina *BAC end sequences; transferability in the *Citrus *genus (Rutaceae), phylogenetic inferences and perspectives for genetic mapping

**DOI:** 10.1186/1471-2164-13-13

**Published:** 2012-01-10

**Authors:** Patrick Ollitrault, Javier Terol, Andres Garcia-Lor, Aurélie Bérard, Aurélie Chauveau, Yann Froelicher, Caroline Belzile, Raphaël Morillon, Luis Navarro, Dominique Brunel, Manuel Talon

**Affiliations:** 1 CIRAD, UMR AGAP, Avenue Agropolis, TA A-108/02, 34398 Montpellier, Cedex 5, France; 2 Centro de Proteccion Vegetal y Biotechnologia, IVIA, Apartado Oficial,46113 Moncada (Valencia), Spain; 3 Centro de genomica, IVIA, Apartado Oficial, 46113 Moncada (Valencia), Spain; 4 INRA, UR EPGV, 2 rue Gaston Cremieux, 91057, Evry, France

## Abstract

**Background:**

With the increasing availability of EST databases and whole genome sequences, SNPs have become the most abundant and powerful polymorphic markers. However, SNP chip data generally suffers from ascertainment biases caused by the SNP discovery and selection process in which a small number of individuals are used as discovery panels. The ongoing International Citrus Genome Consortium sequencing project of the highly heterozygous Clementine and sweet orange genomes will soon result in the release of several hundred thousand SNPs. The primary goals of this study were: (i) to estimate the transferability within the genus *Citrus *of SNPs discovered from Clementine BACend sequencing (BES), (ii) to estimate bias associated with the very narrow discovery panel, and (iii) to evaluate the usefulness of the Clementine-derived SNP markers for diversity analysis and comparative mapping studies between the different cultivated *Citrus *species.

**Results:**

Fifty-four accessions covering the main *Citrus *species and 52 interspecific hybrids between pummelo and Clementine were genotyped on a GoldenGate array platform using 1,457 SNPs mined from Clementine BES and 37 SNPs identified between and within *C. maxima, C. medica, C. reticulata *and *C. micrantha*. Consistent results were obtained from 622 SNP loci. Of these markers, 116 displayed incomplete transferability primarily in *C. medica, C. maxima *and wild *Citrus *species. The two primary biases associated with the SNP mining in Clementine were an overestimation of the *C. reticulata *diversity and an underestimation of the interspecific differentiation. However, the genetic stratification of the gene pool was high, with very frequent significant linkage disequilibrium. Furthermore, the shared intraspecific polymorphism and accession heterozygosity were generally enough to perform interspecific comparative genetic mapping.

**Conclusions:**

A set of 622 SNP markers providing consistent results was selected. Of the markers mined from Clementine, 80.5% were successfully transferred to the whole *Citrus *gene pool. Despite the ascertainment biases in relation to the Clementine origin, the SNP data confirm the important stratification of the gene pools around *C. maxima, C. medica *and *C. reticulata *as well as previous hypothesis on the origin of secondary species. The implemented SNP marker set will be very useful for comparative genetic mapping in *Citrus *and genetic association in *C. reticulata*.

## Background

Single-Nucleotide Polymorphisms (SNPs) are the most frequent type of variation found in DNA [1]. As EST databases and whole genome sequences grow in availability, SNPs have become the most abundant and powerful polymorphic codominant markers that can be selected all along the genome [2]. SNPs allow the implementation of very dense genetic linkage maps in animals and plants [3-5]. Moreover, SNPs are generally considered to have a high identity by descent rate, and thus, they are very useful for genetic association studies [6,7]. The actual array methodologies for the high throughput genotyping of SNPs are built upon the principle of measuring the relative signal strength of two expected alleles [8,9] and require the use of oligonucleotides corresponding to the direct flanking regions of the SNPs. This should present some limitations for germplasm genetic studies. The primary limitation is that the revealed genetic organization of the genotyped germplasm is strongly dependent on the discovery panel [10-15]. This ascertainment bias is particularly noted when SNPs are selected from only one sequenced heterozygous genotype, as proposed in *Vitis vitifera *L. from the whole genome sequence of the cultivar 'Pinot Noir'[16]. Moreover, unexpected alleles may exist at any polymorphism. These unknown or 'null' alleles can interfere with accurate genotyping of the expected alleles, potentially impacting genetic studies in a negative manner [17]. The frequency of these alleles should increase when working with wider genetic distances between the genotyped samples and the discovery panel. A recent review [18] analyzed the importance of the discovery panel and SNP mining methods for genetic studies on plant and animals.

Citrus is the most extensively produced tree fruit crop in the world. Despite controversial *Citrus *classification (in this study, the Swingle and Reece [19] classification is used) most authors now agree on the origin of cultivated citrus species. Scora [20] and Barret and Rhodes [21], working with biochemical and morphological polymorphism, respectively, were the first to suggest that three main primary citrus species originated most of the cultivated citrus: *C. medica *L. (citrons), *C. reticulata *Blanco (mandarins) and *C. maxima *L. Osbeck (pummelos). Molecular marker studies (Isoenzyme [22]; RFLP [23]; RAPD and SCAR [24]; AFLP [25]; and SSRs [26,27] generally support the role of these three taxa as ancestors of cultivated *Citrus*. Furthermore, these studies highlight the probable contribution of a fourth taxon, *C. micrantha *Wester, as the ancestor of limes (*C. aurantifolia *(Christm.) Swingle). All citrus species are fully sexually compatible, capable of producing fertile interspecific hybrids. Thus, they are all part of the same biological species and should probably be considered as separate races, rather than different species. Most modern cultivars have an interspecific origin [28]. All of the secondary species arising from hybridization among the primary species have been clonally propagated (facultative apomixis and horticultural practices), and as such, they present a generally high fixed heterozygosity. Clementine is such a hybrid, vegetatively propagated by grafting from the time it was selected as a chance offspring in a seedling of 'Mediterranean' mandarin (*C. reticulata*) one century ago. A haploid Clementine line has been chosen by the International Citrus Genomic Consortium (ICGC) to establish the reference Citrus whole genome sequence [29,30]. In the framework of the same international project, the diploid Clementine cv 'Nules' has been re-sequenced using new sequencing technologies (454, Roche). SNP density in Clementine has been previously estimated using BACend sequences (BES) to be close to 1 SNP/Kb [31]. As the Clementine haploid genome is estimated at 367 Mb [32], this project is expected to deliver several hundred thousand SNPs all over the *Citrus *genome.

The primary goals of the present study were: (i) to assess the use by array genotyping and the transferability of SNPs discovered from the heterozygous Clementine genome within the *Citrus *genus; (ii) to compare the genetic structure revealed by SNPs heterozygous in Clementine with the structure displayed by SNPs found at the genus level and homozygous in Clementine; (iii) to investigate hypotheses concerning the origin of some secondary species and important cultivars; and (iv) to estimate the usefulness of the Clementine-derived SNP markers for comparative mapping studies between the various cultivated *Citrus *species. For these purposes, 54 *Citrus *accessions and 52 interspecific hybrids between 'Chandler' pummelo and 'Nules' Clementine (CxN) were genotyped on a GoldenGate array platform (Illumina) using 1457 SNPs mined from Clementine cv 'Nules' BES [31] and 37 SNPs mined from between and within *C. maxima, C. medica, C. reticulata *and *C. micrantha*.

## Results

### Design of the *Citrus *Illumina GoldenGate SNP set

#### SNP selection from Clementine BES

Among the 6,617 SNPs mined *in silico *using the POLYBAYES software on 6.14 Mb of assembled sequences from BES, transitions ([A/G]+[C/T]) represented the most abundant changes (3,546; 53.6%). These were followed by transversions ([A/C]+[G/T], 2,162; 32.7%) and InDels (909; 13.7%). According to their probability robustness value, 4,904 transition and transversion SNPs were selected to be tested for their potential technical inclusion on the GoldenGate array. Based on the flanking sequences and absence/presence of additional known SNPs in the vicinity, 2,185 sequences generated a SNP_score greater than 0.6, which was considered the threshold for good marker designability. A total of 768 additional markers exhibited SNP_scores between 0.4 and 0.6 and were associated with a moderate success rate for the marker. Finally, among these 2,953 potential markers, 1,457 SNPs (1,434 with an SNP_score > 0.6 and 23 with an SNP_score between 0.4 and 0.6) were selected for the GoldenGate assay. This selection was based on the SNP distribution on the different BACend contigs and the SNP inclusion or vicinity to coding regions (additional file 1). Respectively, 60.6% were transitions ([A/G]+[C/T] = 883) and 39.4% were transversions ([A/C]+[G/T] = 311; [A/T] = 167; [G/C] = 96).

#### SNP selection from the amplified fragments of gene sequences in the Citrus genus

A total of 6.953 kb were sequenced (Sanger) following the targeted amplification of 10 gene fragments for each of the seven genotypes of the four primary taxa of cultivated species. Two hundred and four SNPs were identified (29.3 SNPs/kb; additional file 2). The designability for the GoldenGate assay was tested using 121 of the identified SNPs. Respectively, 45 and 15 displayed a SNP_score over 0.6 and between 0.4 and 0.6. Thirty seven SNPs were ultimately included in the GoldenGate assay (additional file 1). Of these, 67.5% represented transitions and 32.5% represented transversions.

### Polymorphism and allele call for the different SNPs; selection and classification of valid SNPs

**Figure 1:**
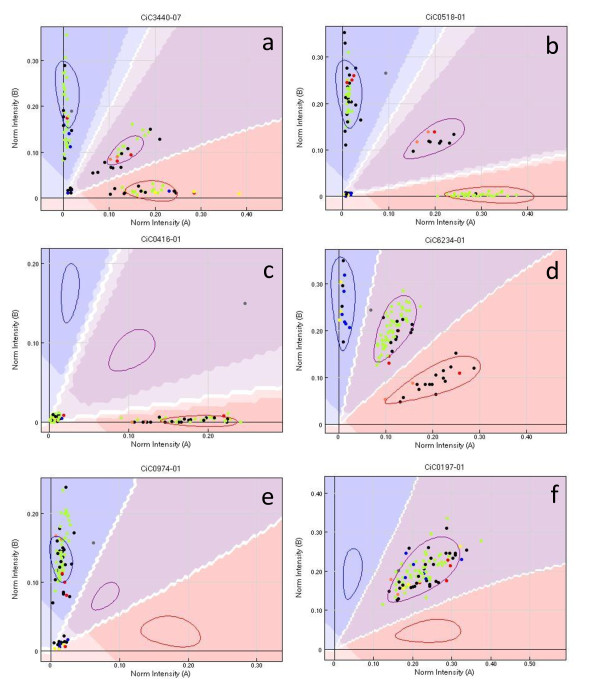
**Unexpected polymorphism distribution**. (a, b, c and d): segregation for null allele in Chandler × Clementine (CxN) population; (a): 0BxAB segregation (C3); (b) 00 × AB segregation (C3); (c) 00 × 0A segregation (C4); (d, e and f): no segregation of Clementine gametes; (d): SNP polymorphism in germplasm (C5); (e): null allele polymorphism in germplasm (C6); (f) potential fixed heterozygosity (duplicated locus; C7). Orange dots: Nules Clementine, blue dots: pummelo germplasm including cv 'Chandler'; red dots: mandarin germplasm; yellow dots: citron germplasm; green dots: C × N progeny

For all SNPs, the genotyping was visually confirmed, taking advantage of the distribution of the CxN progenies relatively to 'Nules' clementine and 'Chandler' pummelo positions. The SNPs were assigned to different categories based on the quality of the polymorphism detected, the detection of null alleles, and the type of segregation observed for 'Nules' Clementine in the CxN progeny (additional file 1). The first category (C1) consisted of 230 markers exhibiting very low technical quality which did not allow for clustering. Among the other categories:

- 608 (C2) displayed the expected segregation for clementine in the CxN progeny. However, for 80 of these loci the clustering between the three classes of genotypes was not totally clear, leading to missing data.

- 85 (C3) with validated heterozygosity in Clementine presented an unexpected segregation, revealing heterozygous (Figure 1a) or homozygous (Figure 1b) null alleles in 'Chandler' pummelo or some others germplasm accessions.

- 28 (C4) displayed segregation of the CxN progeny, supporting a heterozygous null allele in Clementine (ure 1c).

- For the four last categories, no segregation from Clementine was observed in the CxN progeny. Seventy five markers were polymorphic on the whole germplasm sample, displaying homozygous and heterozygous genotypes (C5; Figure 1d); however, 25 were of low quality. Consistent interspecific polymorphisms for a null allele were observed for 32 markers (C6; Figure 1e). For 62 markers, no polymorphism was observed within the sample. However, the cluster position corresponded to an equivalent signal of the two alleles (heterozygous-like, C7, Figure 1f), suggesting possible loci duplication. The last category of 374 markers (C8) consisted of loci with no observed polymorphism. Of the 683 polymorphic loci without null alleles (WONA; C2+C5) and the 145 loci with null alleles (WNA; C3+C4+C6), respectively 506 and 116 markers displaying the clearest differentiation between genotypic classes (unambiguous assigning of genotypes with less than 5% missing data) were selected for further analysis. For the selected WNA and WONA loci, the transition/transversion rate was 59.8/41.1 and 59.5/40.5, respectively. These values are very close to the rate initially observed for the mined SNPs. Respectively, 480 and 26 of the selected WONA loci were from BES and gene sequencing, while all markers with null allele were from BES.

To validate the genotyping data, 24 of the 54 *Citrus *accessions that were genotyped with the GoldenGate array were sequenced (Sanger) for 15 SNPs from five genes. Among the 360 genotype × SNP data, 357 (99.2%) were in agreement with the GoldenGate genotyping and Sanger sequences. In two cases (PSY-C-246 for 'Eureka' lemon and LCY2-P-75 for 'Sunki' mandarin), the GoldenGate genotyping concluded heterozygosity, while the Sanger sequencing inferred homozygosity. The opposite case was obtained with PSY-C-497 in Mexican lime.

The origin of the unexpected polymorphisms displayed by several SNP markers from the Clementine BES, such as null alleles, no heterozygosity for Clementine and 'fixed heterozygosity', was analyzed using Sanger sequencing of the amplicons of four accessions: 'Nules' Clementine, haploid Clementine, 'Chandler' pummelo and Corsican citron.

For 7 loci (CiC0002-01; CiC0049-02; CiC0063-12; CiC0074-09; CiC0091-09; CiC0113-01; CiC2553-04) genotyped homozygous for Clementine in Golden Gate, despite previously being labeled as heterozygous based on BES sequencing, the homozygosity was confirmed by Sanger sequencing. For the analyzed locus of apparent fixed heterozygosity in the PxC progeny (CiC4252-10), the haploid Clementine line also displayed a heterozygous-like pattern, thus confirming the hypothesis of a duplicated locus. For the loci with null alleles, fragment amplification was observed for all of the genotypes. Additional SNPs in the GoldenGate primer area were observed for CiC3064-07 and CiC3275-02 in 'Chandler' pummelo and Corsican citron. However, for another marker of this class (CiC2151-02), no polymorphisms or explanation for the null alleles was found.

### *Citrus *Germplasm diversity for markers without null alleles (WONA loci)

*Population genetic parameters*: detailed data for the 506 markers are given in the additional file 3 and summarized in table 1. The proportion of polymorphic loci was very high within *C. reticulata *(90.7%) while the lower polymorphic loci percentages were observed for *C. medica *(15.4%) and *C. maxima *(26.9%). The average Nei diversity (He) for all of the loci in the entire sample was 0.34, and this varied for individual locus between 0.05 and 0.50 (additional file 3). Thirty-five loci with rare alleles (frequency inferior to 0.05 - ie He < 0.095) were observed. All genotypes were differentiated with the exception of the two *C. aurantium *accessions. The data from the loci determined to be heterozygous (CHet; 476 loci) or homozygous (CHom; 30 loci) in Clementine (table 2) were analyzed separately. Significant differences were observed for the two classes of markers. The level of diversity revealed within *C. reticulata *was strongly reduced for CHom compared with CHet, with respect to the rates of polymorphic loci (56.7%/92.9%), Nei diversity (He: (0.16/0.29), and observed heterozygosity (0.17/0.34). A similar reduction of polymorphism was observed for *C. sinensis *while the average diversity described by the same parameters was significantly higher for CHom for three other secondary species (*C. limon, C. aurantifolia *and *C. paradisi*). No significant differences were observed for *C. medica *and *C. maxima*. For wild *Citrus*, fewer markers were polymorphic when homozygous in Clementine. Throughout the entire sample, the average diversity was slightly higher for the CHom class than with the CHet class (0.403/0.331) with a much stronger population stratification (0.405 and 0.109 F values, respectively).

**Table 1:** Genetic diversity parameters for all loci without null allele (WONA; 506).

	**% L. P**.	MLGs	Ho	He	F
*C. maxima *(10)	26.88	10	0.063+/-0.012	0.063+/-0.011	
*C. medica *(5)	15.42	5	0.089+/-0.021	0.057+/-0.012	
*C. reticulata *(12)	90.71	12	0.327+/-0.019	0.279+/-0.013	
*C. aurantifolia *(4)	54.55	4	0.217+/-0.021	0.186+/-0.015	
*C. aurantium *(2)	54.74	1	0.547+/-0.043	0.273+/-0.021	
*C. limon *(7)	68.97	7	0.364+/-0.030	0.267+/-0.017	
*C. paradisi *(2)	41.70	2	0.416+/-0.042	0.208+/-0.021	
*C.sinensis *(4)	65.61	4	0.653+/-0.041	0.327+/-0.020	
Wild *Citrus *(5)	47.23	5	0.118+/-0.012	0.150+/-0.013	
All samples (54)	100.00	53	0.278+/-0.010	0.336+/-0.011	0.127+/-0.019

%LP: percentage of polymorphic loci, MLGs: number of multilocus genotypes differentiated; Ho: observed heterozygosity; He: Nei diversity; F: Wright fixation index

**Table 2:** Comparison of diversity parameters for loci without null allele (WONA) homozygous (CHom; 30) or heterozygous (Chet; 476) in Clementine.

		**% L. P**.	Ho	He	F
*C. maxima *(10)	CHom	16.67	0.036+/-0.031	0.042+/-0.039	
	CHet	27.52	0.065+/-0.012	0.064+/-0.011	
*C. medica *(5)	CHom	20.00	0.061+/-0.057	0.047+/-0.038	
	CHet	15.13	0.091+/-0.022	0.057+/-0.013	
*C. reticulata *(12)	CHom	56.67	0.166+/-0.075	0.161+/-0.062	
	CHet	92.86	0.337+/-0.019	0.286+/-0.013	
*C. aurantifolia *(4)	CHom	66.67	0.366+/-0.128	0.295+/-0.064	
	CHet	53.78	0.208+/-0.021	0.179+/-0.015	
*C. aurantium *(2)	CHom	43.33	0.433+/-0.180	0.216+/-0.090	
	CHet	55.46	0.554+/-0.044	0.277+/-0.022	
*C. limon *(7)	CHom	80.00	0.579+/-0.136	0.378+/-0.068	
	CHet	68.28	0.351+/-0.031	0.259+/-0.017	
*C. paradisi *(2)	CHom	63.33	0.633+/-0.175	0.316+/-0.087	
	CHet	40.34	0.402+/-0.044	0.201+/-0.022	
*C.sinensis *(4)	CHom	36.67	0.358+/-0.172	0.182+/-0.087	
	CHet	67.44	0.671+/-0.042	0.336+/-0.021	
Wild *Citrus *(5)	CHom	26.67	0.068+/-0.044	0.200+/-0.064	
	CHet	48.53	0.121+/-0.013	0.147+/-0.013	
All samples (54)	CHom	100.00	0.239+/-0.035	0.403+/-0.024	0.405+/-0.081
	CHet	100.00	0.280+/-0.010	0.331+/-0.012	0.109+/-0.019

%LP: percentage of polymorphic loci, Ho: observed heterozygosity; He: Nei diversity; F: Wright fixation index

**Figure 2:**
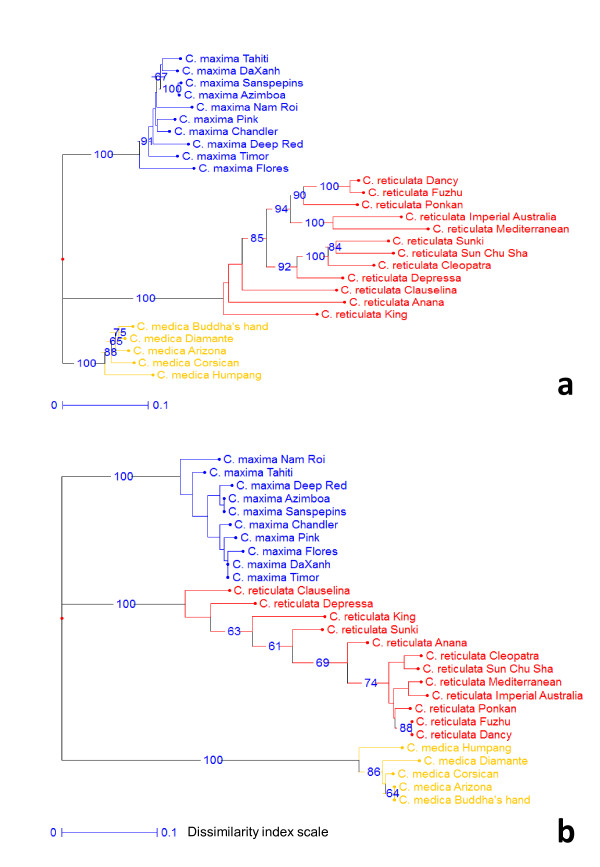
**Cluster analysis (Neighbor Joining) of the accessions of *C. reticulata, C. maxima *and *C. medica *based on loci without null allele (WONA)**. Loci heterozygous in Clementine (CHet; 476); b) Loci homozygous in Clementine (CHom; 30). Numbers near nodes are bootstrap values based on 1000 resamplings (only value > 60% are indicated).

*Structuration between basic taxa*: the analysis of the Fstat parameter in considering the accessions of *C. maxima, C. medica *and *C. reticulata *confirmed the higher differentiation between species with CHom markers (table 3). Indeed, when the Fis value indicated no significant deviance from HWE with the two classes of markers, the significant Fit value (mainly due to between species differentiation with a high positive Fst value) is close to double for the CHom markers (0.80/0.40 for Fit and 0.80/0.45 for Fst). Very similar Fst values are estimated when considering only the differentiation between *C. maxima *and *C. reticulata *(table 3). NJ representations with the two set of markers (Figure 2) clearly shows an increase in inter-specific differentiation with the CHom marker set compared to the CHet set. Particularly, with the CHom markers, *C. medica *is strongly differentiated from the two other species. Moreover, within *C. reticulata*, the dissimilarities between 'Cleopatra', Sun Chu Sha', 'Mediterranean', 'Imperial', 'Ponkan', 'Fushu' and 'Dancy' genotypes appears significantly smaller with CHom than with CHet markers.

**Table 3:** Population organization parameters (Fstat) between and within the three basic taxa and differentiation between *C.maxima *and *C. reticulata *(Fst) evaluated with loci heterozygous (476) or Homozygous in Clementine (30).

	3 basic taxa (*C. maxima, C. medica, C. reticulata*)			*C. maxima* */C. reticulata*
	**Fis**	**Fit**	**Fst**	**Fst**
All loci	-0.09+/-0.02	0.42+/-0.03	0.47+/-0.03	0.46+/-0.03
Het. Cle.	-0.10+/-0.02	0.40+/-0.03	0.45+/-0.03	0.45+/-0.03
Hom. Cle.	0.012+/-0.14	0.80+/-0.10	0.80+/-0.08	0.70+/-0.11

**Figure 3:**
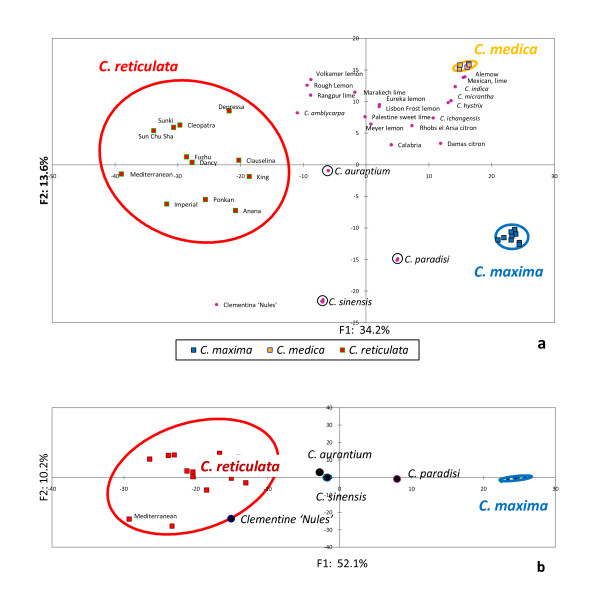
**Principal component analysis from all loci without null alleles (506 loci)**. a) Axes 1/2 with all individual active. b) Positions of secondary species in the axis 1 of an ACP established from only *C. reticulata *and *C. maxima *as active accessions.

**Figure 4:**
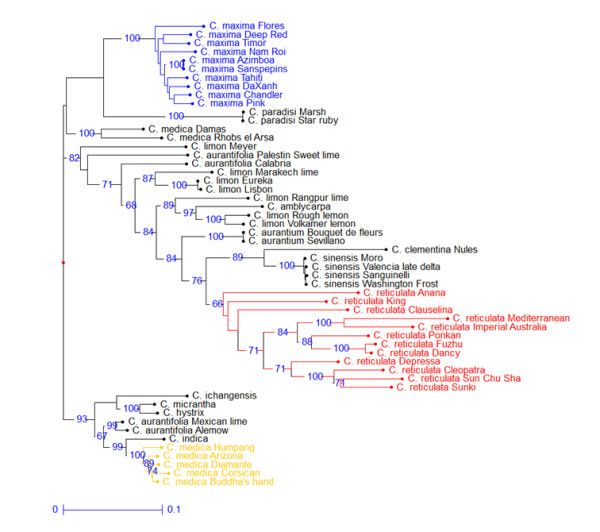
**Neighbor Joining Analysis based on all loci without null alleles (WONA; 506 loci)**. Numbers near nodes are bootstrap values based on 1000 resamplings (only value > 60% are indicated).

*Neighbor joining (NJ) and principal component analysis (PCA) of the whole germplasm sample*: NJ trees are not well adapted to describing the genetic relationships of hybrid structure with the parental gene pool. However, NJ trees allow a global synthetic representation of dissimilarities between genotypes. In factorial analyses such principal component analysis, the hybrid positions between the parents is very clear in the axes that differentiate the parental genotypes. However, representation in a two- or three-axes space may result in a biased view of the global similarities between genotypes. The two representation types (NJA and PCA) are thus complementary when analyzing the genetic relationships and potential parentage of hybrid genotypes. The genetic organization around *C. reticulata, C. medica *and *C. maxima *appears very clearly both with the PCA (Figure 3a) and NJA (Figure 4). Forty-eight percent of the entire diversity is represented by the first two axis of PCA. The first axis discriminates *C. reticulata *from the other species while the second axis separates *C. medica *and *C. maxima*. Very few within species differentiations are observed for the accessions of *C. aurantium, C. sinensis *and *C. paradise*. By contrast, *C. aurantifolia *and *C. limon *display more intra-specific polymorphism. Two accessions of *C. aurantifolia*, 'Alemow' and 'Mexican lime', exhibit an intermediate position between *C. medica *and a papeda cluster including *C. ichangensis, C. micrantha *and C*. hystrix*. The other two *C. aurantifolia *accessions (Calabria and Palestine sweet lime) are more related to the *C. limon *accessions. The *C. limon *accessions are subdivided into three close clusters: Meyer lemon is the more isolated, while 'Eureka', 'Lisbon' and 'Marrakech lime' are clustered to one side and 'Rangpur' lime, 'Rough lemon' and 'Volkamer' lemon cluster in the other side. This last cluster presents higher coordinates in the second axis of the PCA(a) and lower coordinates in the first axis, compared to the first lemon cluster. This suggests a higher contribution of *C. reticulata *and very few or no *C. maxima *contribution. Interestingly, a sub cluster of acidic mandarin ('Cleopatra', 'Sunki', 'Depressa' and 'Sun Chu Sha') also present higher coordinates in the second axis relative to the other *C. reticulata *accessions. Sweet and sour oranges are strongly differentiated by the second axis. Clementine is very close to sweet orange in this axis and is intermediary between Mediterranean mandarin and sweet orange in the first axis. To analyze the contribution of *C. maxima *and *C. reticulata *to the genome of their supposed deriving secondary species (*C. sinensis, C. aurantium *and *C. paradisi*), a second PCA was generated using only *C. maxima *and *C. reticulata *as active individuals to define the new axes (Figure 3b). Of the total diversity, 52.1% was supported by the first axis opposing the two species. The contribution of the SNP loci to the definition of this axis (estimated by the cos^2 ^of the coordinate in the new components) presents a very high correlation with the Fst value for the *C. maxima/C. reticulata *differentiation (r^2 ^= 0.948, additional file 4, figure S1). This confirms the validity of this axis for the synthesis of the relative contribution of the two basic species to the secondary ones. *C. sinensis *and *C. aurantium *display similar positions in this axis at a closer distance to the *C. reticulata *gene pool than the *C. maxima *one. *C. paradisi *has an intermediary position between these two secondary species and *C. maxima*.

*Linkage disequilibrium in the germplasm and segregating population*: 472 of the 476 WONA markers heterozygous for Clementine have been successfully genotyped (less than 5% missing data) in the 'Chandler' pummelo × 'Nules' Clementine progeny (CxN). A comparative analysis of the LD within the Citrus germplasm sample and within the CxN segregating population was done for all of the pairs of the 472 markers. The average LD (estimated by r^2^) was 0.152 and 0.060 for germplasm and CxN, respectively. A r^2 ^> 0.2 is generally considered to be a threshold for significant LD between marker pairs. Using this criteria, 27% and 6% of the locus pairs displayed significant LD within the germplasm and CxN populations, respectively. The proportion of significant LD was estimated using the exact test p-value at 5% and 1% thresholds. Regardless of the parameter used, the proportion of significant LD was much higher within the germplasm sample than with the segregating CxN population (table 4). Upon analyzing the co-distribution of LD for the germplasm and segregating populations (Additional file 4, figure S2), an important proportion of significant LD were observed within the germplasm population for the loci pairs with r^2 ^< 0.1 in the segregating CxN population.

**Table 4:** LD between 472 SNP loci within the Chandler × Nules progeny and germplasm samples.

	Average r^2^	r^2 ^> 0.2	pvalue < 5%	pvalue < 1%
Chandler × Nules	0.060	7015 (6.31%)	12962 (11.66%)	8488 (7.64%)
Germplasm	0.152	29813 (26.82%)	65758 (59.15%)	49169 (44.23%)

### *Citrus *Germplasm diversity displayed by markers with null alleles (WNA loci)

Detailed data for all WNA markers are listed in additional file 5 and summarized in table 5. Among the 116 markers with null alleles, 88, 82, 31 and 92 display null alleles for at least one accession within *C. maxima, C. medica, C. reticulata *and wild *Citrus*, respectively. Interestingly, for *C. reticulata*, nine of the 31 considered markers exhibit null allele for only one accession. The frequency of genotypes that are homozygous for the null allele is very high in *C. medica *(0.641), *C. maxima *(0.609), wild *Citrus *(0.474) and *C. aurantifolia *(0.405), but low in *C. reticulata *(0.111) and *C. sinensis *(0.086).

**Table 5:** Diversity parameters for loci with null alleles (WNA)

	**% L. P**.	MLGs	GD	Null	Ho
*C. maxima *(10)	41.38	10	0.145+/-0.036	0.609	0.037
*C. medica *(5)	17.24	5	0.060+/-0.025	0.641	0.041
*C. reticulata *(12)	80.17	12	0.361+/-0.040	0.111	0.234
*C. aurantifolia *(4)	70.69	4	0.308+/-0.038	0.405	0.045
*C. aurantium *(2)	15.52	2	0.004+/-0.008	0.103	0.151
*C. limon *(7)	73.27	6	0.317+/-0.041	0.190	0.086
*C. paradisi *(2)	8.62	1	0+/-0	0.224	0.086
*C. sinensis *(4)	24.13	1	0/-0	0.086	0.241
Wild *Citrus *(5)	76.72	5	0.329+/-0.037	0.474	0.036
All samples (54)	100	47	0.546+/-0.021	0.326	0.121

%LP: percentage of polymorphic loci, MLGs: number of multilocus genotypes differentiated; GD: genotypic diversity; Null: average proportion of Homozygous for null allele; Ho: average proportion of observed heterozygous (2 different bases).

The within species discrimination of accessions obtained with the whole set of WNA markers is respectable, with the exception of *C. sinensis *and *C. paradisi. C. reticulata*, the most polymorphic species, displayed a genotypic diversity of 0.361 and an observed heterozygosity of 0.234. The observed heterozygosity of all of the secondary species is strongly reduced when compared with the WONA loci, suggesting frequent but not observed heterozygous null alleles.

**Figure 5:**
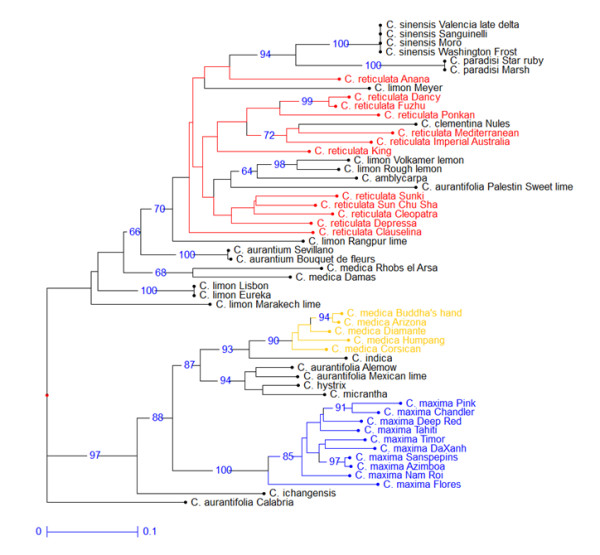
**Neighbor Joining Analysis based on SNP loci with null alleles (WNA; 116 loci)**. Numbers near nodes are bootstrap values based on 1000 resamplings (only value > 60% are indicated).

The differentiation of the three basic species (*C. maxima, C. medica *and *C. reticulata*) was confirmed by the NJA based on WNA loci (Figure 5). However, the global picture displayed by this analysis varies significantly from the previous one without null alleles (Figure 4). Indeed, *C. maxima, C. medica*, most wild C*itrus *species (except *C. amblicarpa*), as well as two *C. aurantifolia *accessions (Mexican lime and Alemow) are strongly clustered. The numerous loci sharing null alleles between *C. medica, C. maxima *and some wild species may explain this strong clustering as well as the position of supposed hybrids between these taxa (Mexican lime and Alemow). Moreover, *C. sinensis, C. paradisi *and several *C. limo*n and *C. aurantifolia *accessions are integrated within the *C. reticulata *cluster. These secondary species are suspected to be hybrids between *C. reticulata *and *C. maxima *and/or *C. medica*. Due to the recessive nature of null alleles, these secondary species appear artificially closer to their *C. reticulata *parent because of the lower frequency of null alleles in this gene-pool compared with the other ancestral species. An interesting point is that the recessive nature of a high proportion of alleles from the other species allows the approximation of the sub gene pool within *C. reticulata *at the origin of some interspecific hybrids. Clementine is clustered with 'Mediterranean' mandarin. The parentage of sweet orange and grapefruit is also clearly revealed. Volkamer and Rough lemons, as well as Palestine Sweet lime, are clustered with a group of acidic mandarins (Sunki, Sun Chu Cha, Cleopatra and Depressa). *C. amblycarpa *is also associated with this cluster.

### Markers for interspecific comparative genetic mapping

The number of loci that could be potentially mapped was estimated as the number of polymorphic loci within each species. For the WNA markers, the SNPs as well as the null allele polymorphisms were considered. As Clementine was at the origin of most of the selected SNPs markers, 'Nules' Clementine was logically the cultivar allowing the most complete genetic mapping with a total of 567 mappable markers (table 6). At the intraspecific level, the usefulness of the selected SNP loci is high for *C. reticulata *(554), *C. limon *(460), *C. aurantifolia *(377) and *C. sinensis *(361), moderate for *C. paradisi *(221) and *C. maxima *(189), low for *C. medica *(99), and very low for *C. micrantha *(17). Compared to the proportion for WONA loci, the proportion of useful WNA loci appears low for *C. paradisi *(10/116), *C. sinensis *(29/116) and *C. aurantium *(18/116), but very high for *C. reticulata *(93/116), *C. limon *(85/116) and *C. aurantifolia *(82/116). The highest number of loci for comparative mapping using WONA markers was observed for *C. reticulata*/*C. limon *(350), followed by *C. reticulata*/*C. sinensis *(301). For WNA markers, the higher values were found for *C. aurantifolia*/*C. limon *and *C. aurantifolia*/*C. reticulata *(63 markers for both interspecific comparative mapping).

**Table 6:** Mappable loci for comparative genetic mapping.

	Clem	***C. max***.	***C. med***.	***C. ret***.	*C. arf*	***C. aur***.	***C. lim***.	***C. par***.	***C. sin***.	***C. mic***.
Clementine	**567**	36	13	67	51	13	58	6	26	2
*C. maxima*	136	**189**	9	40	27	15	37	5	18	2
*C. medica*	73	28	**99**	16	17	5	16	3	4	1
*C. reticulata*	444	122	73	**554**	63	17	76	9	26	2
*C. aurantifolia*	271	78	52	264	**377**	15	63	8	21	2
*C. aurantium*	264	64	47	260	207	**295**	15	5	11	2
*C. limon*	350	88	63	350	263	256	**460**	9	23	2
*C. paradisis*	192	57	38	193	142	139	171	**221**	7	1
*C. sinensis*	321	101	49	301	216	208	269	192	**361**	2
*C. micrantha*	14	6	6	15	13	11	13	10	10	**17**

Total WONA	476	141	79	461	295	277	375	211	332	15
Total WNA	91	48	20	93	82	18	85	10	29	2

Above the diagonal, loci with null alleles (WNA) polymorphic within each species; below the diagonal, loci without null alleles (WONA) polymorphic within each species; diagonal in bold, total number of loci mappable in one species (both WNA and WONA).

## Discussion

### SNP mining in Clementine and the unexpected segregations in Chandler × Clementine progeny

Among the 6,617 SNPs mined *in silico*, 1,457 markers were selected for the GoldenGate assay based on their distribution on the different BACend contigs, as well as their inclusion in or their vicinity to the coding region. Thirty seven SNP loci found in 10 candidate genes were added for the analysis. Low technical quality was displayed by 230 markers, preventing any clustering. A total of 693 markers presented segregations that conformed to Clementine heterozygosity (C2+C3). The 'Chandler' × Clementine (CxN) progeny revealed heterozygous null alleles in Clementine for 28 markers (C4). Moreover, 481 markers appeared to be homozygous for Clementine (C5+C6+C8) while 472 of them were supposed to be heterozygous from the Clementine BACend sequencing [31]. This homozygosity was confirmed using Sanger sequencing for the seven tested markers. SNP analysis on BAC ends was carried out by analyzing nucleotide variation within assembled reads in one contig, each reading originated from different *E. coli *clones. A base miscall in one of the BAC end reads, or even a mutation introduced by the E. coli replication machinery in the BAC sequence, could create a false SNP that is not present in the genomic sequence, as it was confirmed by direct Sanger sequencing of the genomic DNA amplified by PCR. For 62 markers, potential locus duplication (C7) was suspected and confirmed for the tested locus by the heterozygous profile for the haploid Clementine line selected for whole genome sequencing [29]. Moerover multiple blasts in the reference citrus whole genome sequence (http://www.phytozome.net/clementine.php) of the corresponding sequences comfort this hypothesis (data not shown). Five hundred ninety six of the SNPs mined in Clementine BES and 26 from targeted gene sequencing were finally selected for genetic analysis. The validation of the SNP genotyping for 15 SNPs × 24 genotypes was conducted by Sanger sequencing and a validity rate of 99.2% was obtained.

### GoldenGate SNP marker transferability and loci with null allele usefulness

Marker transferability was estimated by null allele identification and dispersion in the gene pool. The null alleles may result from unexpected polymorphisms affecting the allele amplification/hybridization on the GoldenGate array. This may result from deletions spanning a polymorphic site [33,34], secondary polymorphisms interfering with genotyping at the primary polymorphic target (as was observed for two markers with Sanger sequencing of the *C. maxima *and *C. medica *accessions), and even unexpected alleles at the primary polymorphism (such as triallelic sites; [35]). All of these are important potential sources of reproducible, but inaccurate, genotypes for population genetic studies because heterozygous null alleles are indistinguishable from the expected homozygotes on most genotyping platforms. In this study, among the high quality markers, 506 WONA and 116 WNA loci were selected. The transferability of SNPs to the whole *Citrus *genus can thus be estimated as 506/622 = 81.4% if all loci are considered, or as 480/596 = 80.5% considering loci identified in the Clementine BES. The null alleles were primarily found in *C. medica, C. maxima*, and wild *Citrus *with an average homozygosity frequency for null alleles being 0.64, 0.61 and 0.47, respectively, but only 0.11 in *C. reticulata*. As these markers were identified from the Clementine BES and GoldenGate primers defined from Clementine sequences, these results are reasonable based on the strong genetic relationship between Clementine and *C. reticulata *and the important inter-specific differentiation between *C. reticulata *and the others basic taxa [28]. For the secondary species resulting from hybridization between the *C. reticulata *gene pool and the other basic taxa, the WNA loci present the advantage of frequent recessivity for the other parental gene pools. Therefore, it may allow identification of the *C. reticulata *subset that contributed to the secondary species genesis. Moreover, heterozygous null alleles should be useful for genetic mapping.

### SNPs mined in a single heterozygous genotype provide a distorted view of the gene pool diversity but confirm the high stratification of the *Citrus *genus

The selection of heterozygous markers in Clementine primarily affects the estimation of two components of genetic diversity. The first is the differentiation between the basic taxa that appears to be underestimated with CHet loci when compared with CHom loci. The second is the intra-specific diversity. *C. reticulata *within diversity (both intercultivar and heterozygosity) and *C. sinensis *heterozygosity appear to be overestimated using the CHet loci when compared with the CHom loci. This is in contrast with the results obtained for *C. paradisi, C. aurantifolia *and *C. limon*. Previous studies have shown that Clementine is highly related to *C. reticulata *with a limited introgression of *C. maxima *[24,28,36]. Therefore, it can be inferred that the majority of the Clementine heterozygosity arose from the *C. reticulata *gene pool diversity. This may explain the overestimation of *C. reticulata *diversity when compared to *C. maxima *and *C. medica*, as well as the underestimation of the *Citrus *gene pool stratification. The genetic constitution of *C. sinensis *(mainly issued from the *C. reticulata *gene pool; see below for more detail) may also explain its higher heterozygosity for the CHet markers. According to their supposed origin (see below), the heterozygosity of *C. limon *and *C. aurantifolia *is based on the interspecific differentiation between the basic taxa. On the other hand, *C. paradisi *arose from *C. maxima/C. reticulata *differentiation and *C. maxima *within diversity. Thus, the underestimation of interspecific differentiation and the underestimation of *C. maxima *within diversity with the CHet markers explain the underestimation of diversity and heterozygosity of the above mentioned secondary species. In the present study, very low intraspecific polymorphism was identified for *C. maxima *and *C. medica*, regardless of the heterozygosity of the markers in Clementine. However, previous SSR studies reported similar within species diversity in *C. maxima *and *C. reticulata *[27,28]. It is highly probable that the set of markers used in this study target primarily within *C. reticulata *polymorphisms from one side and interspecific polymorphisms from the other side. Overall, the results reported here illustrate the limit and bias of the SNP array approach for large diversity analysis on a highly stratified population when the SNP discovery is based on a very limited panel. The SNP ascertainment bias has been widely discussed in humans [10,12] and animals [11,13-15] in relation with geographical stratification. This study reports bias associated with 'racial' differentiation in cultivated plants. This bias is enforced when taking into account the WNA loci. Future accurate analysis of the interspecific mosaic structure of secondary species and intra- and interspecific polymorphism analysis should be based on a non-biased pangenomic set of markers. The availability of a reference citrus genome sequence [29,30] and the new sequencing methodologies will soon allow these objectives to be re-visited by the resequencing of several accessions of the basic taxa and secondary species.

Despite the distorted view of the gene pool diversity, the global organization around the basic taxa is still clear in both the PCA and NJ representation based in the WONA loci. The analysis of Fstat parameters on the subset of the genotypes of the three basic taxa (*C. reticulata, C. medica, C. maxima*) with a non-significant Fis value but high Fit and Fst values confirms this important organization of the allelic diversity between these taxa. Moreover, a very high proportion of loci pairs display significant linkage disequilibrium in the germplasm sample. The majority of these locus pairs with significant LD in the germplasm sample appear in equilibrium within the segregating 'Chandler pummelo × Nules Clementine' (CxN) population, testifying for very extended LD in the *Citrus *genus. Similar results were observed by Garcia-Lor et al. [28] for SSRs and InDel markers with significant LD for loci situated in different linkage groups. Breeding systems and domestication history are determinant factors of the LD structure in the germplasm of cultivated plants [37,38]. The extent of LD is generally greater for species that possess a selfing mating system [39-41] than for outcrossing ones [42-44]. The heterozygous deficit and generalized linkage disequilibrium observed in the *Citrus *genus indicates a strong population subdivision and thus a low gene flow between *C. medica, C. reticulata, C. maxima *and wild *Citrus*. The differentiation between these sexually compatible taxa may be explained by foundation effect in three geographic zones and by an initial allopatric evolution. *C. maxima *originated in the Malay Archipelago and Indonesia, *C. medica *evolved in north-eastern India and the nearby region of Burma and China, and *C. reticulata *diversification occurred over a region including Vietnam, southern China and Japan [20,45]. Secondary species arose from the hybridization of the basic taxa. The partial apomixis of most of the secondary species has certainly been an essential element in the limitation of gene flows after that human activities have put into contact the differentiated gene pools of the basic taxa. Apomixis may also explain that, in agreement with previous molecular studies [27,28,46], very few polymorphisms were found between the analyzed genotypes within *C. sinensis, C. aurantium *and *C. paradisi *although they were highly heterozygous (Ho of 0.65, 0.55 and 0.42, respectively, with the whole set of WONA markers). This confirms that most of the intra-specific polymorphism of these secondary species arose from punctual mutation, transposable element movement [47] or epigenetic variation.

### Some parentage hypotheses for secondary species are strongly comfirmed

**Figure 6:**
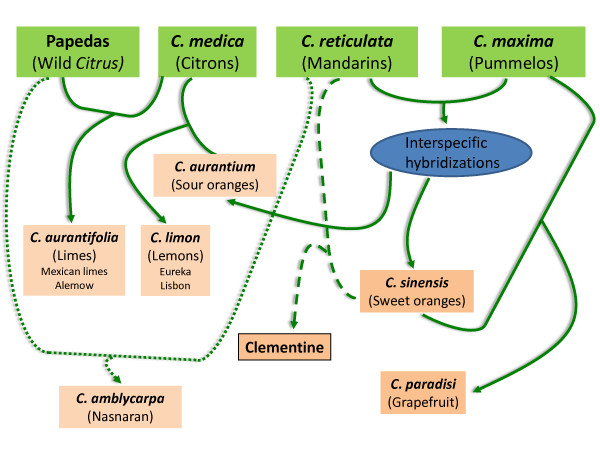
**Hypothesis on the origin of some secondary *Citrus *species**.

The parentage hypothesis of some very important commercial species or cultivars was checked using their position in the PCA and NJA representations and the loci count for which the hybrid genotype disagrees with the supposed parent ones (data not shown based on the 506 WONA loci). The synthesis of the parentage hypothesis is given in Figure 6.

*Sweet orange (C. sinensis) and sour orange (C. aurantium)*: these two species are believed to derive from hybridizations between the *C. maxima *and *C. reticulata *gene pools [24,27,48]. Their positions in the PCA analysis with *C. reticulata *and *C. maxima *used as active individuals confirm that a predominant portion of their genome arose from the *C. reticulata *gene pool, as previously deduced from SSR markers [28,49] .

*Clementine*: it is generally agreed that Father Clement selected, a little more than one century ago in Algeria, Clementine as a chance seedling from 'Mediterranean' mandarin. The mandarin female parentage was confirmed by mitochondrial genome analysis [50]. 'Granito' sour orange was initially considered to be the male parent. However, previous molecular studies suggested that Clementine was more likely a mandarin × sweet orange hybrid [24,36]. The position of Clementine relative to sweet orange, sour orange and 'Mediterranean' mandarin in the PCA analysis supports this hypothesis. The hypothesis of a 'Mediterranean' mandarin × sweet orange is definitively authenticated with only one locus out of 506 suggesting incompatible genotypes, while 86 loci disagree with the hypothesis of an hybridization between 'Mediterranean' mandarin and sour orange.

*Grapefruit (C. paradisi)*: the origin of grapefruit is attributed to a natural hybridization between pummelo (*C. maxima*) and sweet orange (*C. sinensis*). This hybridization may have occurred in the Caribbean more than 200 years ago [21,51-53]. In this study, grapefruit had an intermediary position between the sweet orange and pummelo gene pool in the PCA representation. Moreover, the NJA for the WNA markers clearly demonstrates the relationship of grapefruit and sweet orange. According to the sweet orange/pummelo combination, 96.3% to 98.0% of the 506 analyzed markers agree with this hypothesis. The best fitting is found with Tahiti pummelo. When searching for a potential sour orange × pummelo origin, the percentage of loci in disagreement varies between 12.5% and 14.5%. *C. maxima *is a polymorphic non-apomictic species. Therefore, due to the limited number of pummelo accessions analyzed, the absence of a 100% fit is reasonable. Moreover, as previously discussed, null alleles are relatively frequent in pummelo and it is likely that some of them have not been identified due to their heterozygous status. Therefore, the absence of some parental alleles in grapefruit, although they seem homozygous in one of the parents, may be explained by heterozygous null alleles for this parent. Upon looking for alleles present in grapefruit but absent in the two potential parents, only one to four loci disagree according to the considered pummelos. Thus, the data strongly confirm the hypothesis of the sweet orange × pummelo hybridization.

*'Eureka' and 'Lisbon' lemon*: Based on RFLP, RAPD and CAPS data, Nicolosi et al. [24] were the first to propose that lemon arose from a hybridization between *C. aurantium *and *C. medica*. This hypothesis was supported by nuclear SSR analysis [28]. In the present study, 'Eureka' and 'Lisbon' lemon varieties were highly heterozygous and very similar. These lemons are very likely two somatic mutants of the same ancestor. In PCA, their position was between the *C. aurantium *and *C. medica *group in each factorial axis. According to the citron accession, 96.0% to 97.8% of the 506 analyzed markers agree with this hypothesis. Moreover, null alleles are relatively frequent in citron and pummelo (contributing to sour-orange genesis). As for grapefruit, a search for alleles present in lemon but absent in the two potential parents reveals only one locus (CiC4841-04) out of 506 that disagrees with this hypothesis.

*Mexican lime and Alemow*: these two citrus were considered two distinct species, *C. aurantifolia *and *C. macrophylla*, respectively, by Tanaka [54]. However, Swingle and Reece [19] joined the two in *C. aurantifolia*. In all of the analysis reported here, these two were very close and intermediary between the citron cluster and a papeda cluster including *C. hystrix *and *C. micrantha*. For Mexican lime, this position is in agreement with the hypothesis proposed by Nicolosi et al. [24]. These authors proposed that Mexican lime was a hybrid between *C. micrantha *x *C. medica*. The maternal phylogeny was recently confirmed by Froelicher et al. [50]. According to the citron cultivar, 97.4% to 98.5% of the 506 analyzed markers agree with the *C. micrantha *x *C. medica *hypothesis with 'Humpang' citron providing the best fit. Moreover, no allele observed in Mexican lime was absent in the two potential parents. A *C. hystrix *x *C. medica *hypothesis produced very close results (97.3% to 97.7%). Very similar results were observed for 'Alemow' with 96.7% to 97.4% and 96.0% and 96.9% of loci in agreements with *C. micrantha *x *C. medica *and *C. hystrix *x *C. medica *origins, respectively. The papeda maternal parentage of Alemow was demonstrated by mitochondrial genome analysis [50]. Mexican lime and Alemow clearly have similar origins, and the papeda × *C. medica *hypothesis was confirmed by this data. An enhanced study of papeda germplasm will be necessary to definitively conclude *C. micrantha *or another papeda as the female parent.

*C. amblycarpa *is native to Indonesia where it is called Djerook leemo (http://www.ars-grin.gov/cgi-bin/npgs/html/taxon.pl?10679). It is generally considered to be a mandarin hybrid, and its common English name is Nasnaran mandarin. However, Froelicher et al. [50] showed that it has a papeda mitotype, identical to *C. micrantha *and *C. hystrix*. In PCA, *C. amblycarpa *displays an intermediary position between the two mentioned papedas and the acidic mandarin group ('Depressa', 'Sunki', 'Cleopatra' and 'Sun Chu Cha' mandarins). Its relationship with these mandarins is confirmed by the sharing of the same cluster in the NJA based on the WNA loci. A search for a potential direct papeda × *C. reticulata *origin was conducted. The best observed fit was a *C. amblycarpa *x 'Sun Chu Cha' mandarin hybridization with 92.5% of loci in agreement. For only 0.4% of the loci, one allele absent in the two parents was found in *C. amblycarpa*. Therefore, the hypothesis of papeda × acidic mandarin is proposed for *C. amblycarpa*.

### A very useful set of SNPs markers for the comparative genetic mapping in *Citrus *and association genetic studies in *C. reticulata*

Compared with other crops, genetic mapping in citrus is still undeveloped. The recent genetic maps based on codominant markers (primarily SSRs) [55-57] integrated around 150 markers, while maps based on dominant markers such as AFLPs [58], SRAPs, ISSRs, and RAPDs [59] included a little more than 200 markers. The markers mined in Clementine BES will be very useful for genetic mapping and association genetics in *C. reticulata *(554 polymorphic loci). The usefulness of these markers is more limited for the other basic taxa with 189, 99 and 17 polymorphic markers within *C. maxima, C. medica *and *C. micrantha*, respectively. For the secondary species, the marker number varies from 221 for *C. paradisi *to 460 for *C. limon*. For *C. sinensis, C. aurantium *and *C. paradisi*, where very little inter-cultivar diversity was found, the numbers of polymorphic loci are very similar to the number of heterozygous loci in a concrete genotype (and thus, directly mappable from a segregating population having such a genotype as a parent). For the other species, a consensus map should be established from several progenies to map all of the indicated markers. Moreover, it is probable that secondary species, such as *C. paradisi, C. sinensis, C. aurantium *and *C. limon*, have numerous heterozygous null alleles (inherited from the *C. maxima *or *C. medica *gene pools) for WNA loci in phylogenetic heterozygosity (*C. reticulata/C. maxima *or *C. reticulata/C. medica*). Therefore, the real number of mappable loci should be higher for these species. The biallelic nature of SNP markers limits the possibility to establish two anchored maps (male and female) from a single cross. This is because the allelelic phase of the markers heterozygous for the two parents can only be inferred for homozygous progenies. Multiallelic markers like SSRs are more powerful for such application [46].

The 547 markers heterozygous in Clementine are currently mapped in the framework of the International Citrus Genome Consortium (ICGC; [60]) and will contribute to the assembly of the reference citrus whole genome sequence. Interestingly, at least 346 of these markers should also be mapped on sweet orange in order to anchor the sweet orange genetic map developed by an US consortium [57] and the Clementine map to establish a saturated consensus citrus genetic map. Furthermore, it is also notable to mention that a large proportion of the analyzed SNPs are located in or close to putative coding regions [31]; therefore, these 'functional SNPs' may provide an important resource for the identification of genes associated with specific trait loci.

## Conclusions

A set of 622 SNP markers providing consistent results was selected. Of the selected markers mined in Clementine BES, 80.5% were successfully transferred to the whole *Citrus *gene pool. The 116 loci with incomplete transferability displayed null allele homozygotes primarily in *C. medica, C. maxima *and wild *Citrus *species. The recessivity of the null alleles from these basic species should be useful in the identification of the subgene pools of *C. reticulata *at the origin of several interspecific hybrid species or varieties. Heterozygous null alleles should be useful for genetic mapping, particularly in secondary species. The two main biases associated with the SNP mining in Clementine were an overestimation of *C. reticulata *diversity and an underestimation of interspecific differentiation. However, the organization of the gene pool remained important, with high interspecific Fst values and very frequent significant linkage disequilibrium between markers pairs in equilibrium in the segregating population. Thus, despite the ascertainment biases, the SNP data confirms the important stratification of the gene pools around *C. maxima, C. medica *and *C. reticulata*, as well as the previous hypothesis on the origin of secondary species. The shared intra-specific polymorphism and accession heterozygosity will permit interspecific comparative genetic mapping. The implemented SNP marker set will also be very useful for association genetic studies in *C. reticulata*.

## Methods

### Plant material

In addition to Clementine cv 'Nules' (whose BES were used for SNP mining), 53 varieties from the citrus germplasm bank of IVIA (Spain) and INRA/CIRAD (France) were used for the transferability and diversity study of SNPs within the *Citrus *Genus. According to the Swingle and Reece classification [19] and the Nicolosi et al. [24] hypothesis on the origin of cultivated citrus species, 29 belong to the three primary species (12 *C. reticulata*, 10 *C. maxima *and seven *C. medica*), 19 represent secondary species (two *C. aurantium*, four *C. sinensis*, two *C. paradisi*, seven *C. limon *and four *C. aurantifolia*), and five are wild species (additional file 6). Among the *C. medica *accessions, the present work confirmed the previously doubted classification of two cultivars as true citron ('Damas' and 'Rhob el Arsa' cultivars). These cultivars were thus excluded from the evaluation of within species diversity and between species organization of diversity.

A segregating population of 52 interspecific hybrids of 'Chandler' pummelo × 'Nules' Clementine (CxN, developed by Cirad in Corsica) was used to confirm Clementine heterozygosity. This population was helpful in making the genotypic assignments of the germplasm samples and in comparing the linkage disequilibrium (LD) distribution of the germplasm (depending on the evolutionary history of the gene pool and marker linkage) and the segregating population (depending only on the marker linkage).

### DNA extraction

Total DNA was extracted from fresh leaves according to Doyle and Doyle [61].

### SNP mining from Clementine BACend sequences

As described in Terol et al. [31], *in silico *SNP mining was performed from 46,339 *C. clementina *cv. Nules BACend sequences (BESs) covering 28.1 Mb of genomic sequences. Assembly of BESs that did not contain repetitive sequences was performed using CAP3 [62]. A total of 6,461 contigs, including 19,057 reads and covering 6.14 Mb of sequence, were produced. The SNPs were mined in these contigs using POLYBAYES software. A total of 6,617 putative SNPs (1.08 SNPs per kb) were found. A total of 4,904 SNPs were *in silico *tested for their potential use in the Illumina Golden Gate array following the Illumina procedure.

### SNP mining in candidate genes

In an effort to identify SNPs within the *Citrus *genus, two cultivars of *C. medica *(Corsican and Budha's hand citrons), two cultivars of *C. reticulata *('Cleopatra' and 'Mediterranean' mandarin), two cultivars of *C. maxima *('Chandler' and 'Pink' pummelos) and one *C. micrantha *accession were selected. Primers (additional file 2) were defined from EST sequences available in the public databases for six genes implicated in primary and secondary metabolite biosynthesis pathways involved in determining citrus fruit quality (sugars, acids, flavonoids and carotenoids: Chalcone isomerase -CHI-, Vacuolar citrate/H+ symporter -TRPA-, Phosphofructokinase -PKF-, Lycopene β-cyclase -LCY2-, Phytoene synthase -PSY-, Lycopene β-cyclase -LCYB-) and four candidates genes linked to salt tolerance (Cation/H+ antiporter -CAX-, Ascorbate oxydase -AOC-, High-affinity K+ Transporter 1 -HKT1- and Tréhalose-6-Phosphate Synthase -TS-). PCR amplifications of the samples were performed using a Mastercycler EP Gradient S thermocycler (Eppendorf) in a 100 μL final volume containing 0.025 U/μL of Pfu DNA polymerase (Fermentas), 2 ng/μL of genomic DNA, 0.2 mM of each dNTP, 2 mM MgSO_4_, 75 mM Tris-HCl (pH 8.8), 20 mM (NH_4_)_2_SO_4_, and 0.2 μM of each primer. The following PCR program was used: denaturation at 94°C for 5 min; 35 repetitions of 30 s at 94°C, 1 min at 55°C or 60°C (according to primer T_m_), and 45 s at 72°C; and a final elongation step of 4 min at 72°C. PCR product purification was performed using the QIAquick^® ^PCR Purification Kit (Qiagen). Amplicons of the seven selected genotypes were sequenced by the Sanger method from the 5' extremity using dideoxynucleotides marked with fluorescence (Big Dye Terminator Cycle Sequencing Kit v3.1). Sequences were aligned with BioEdit [63] and SNPs were detected along the sequences. Of the identified polymorphisms, 121 were *in silico *tested for their potential use in the Illumina GoldenGate assay.

### SNP genotyping with an Illumina GoldenGate microarray

For each selected SNP locus, three primers were designed using the Illumina Assay Design Tool (https://icom.illumina.com). Sequence and primer information for the selected SNPs are listed in the additional file 1. The DNA of the samples to be genotyped was quantified according to Illumina specifications using PicoGreen (Molecular Probes) and a Gemini XPS Fluorescence Microplate Reader (Molecular Devices). The genotyping reactions were performed according to the standard Illumina GoldenGate assay instructions (http://www.illumina.com). In brief, 250 ng of template DNA was used per sample. SNP-specific oligonucleotides containing both detection specific sequences and universal primer sequences were hybridized, extended and ligated to a common oligonucleotide containing a universal primer sequence. Ligated products were amplified using a universal primer set. Genotypes were determined by hybridizing the amplified products to a bead array which was complementary to the sequence specific tags. The fluorescence of the bead array was determined using a Bead Array Reader (Illumina). Two genotype controls ('Nules' Clementine and 'Chandler' pummelo) were repeated twice in each plate. The data were collected and analyzed using the Genome Studio software (Illumina). The automatic allele calling was visually checked and corrected if necessary, taking advantage of the segregating Pummelo × Clementine population.

### SNP genotyping validation by Sanger sequencing of amplicons

Of the 54 *Citrus *accessions genotyped with the GoldenGate array, 24 were used to validate the genotyping data for 15 SNPs from five genes (*LCY2, LCYb, PKF, PSY, TRPA*). This subset included 'Nules' Clementine, seven accessions of *C. reticulata*, five *C. maxima*, four *C. medica*, two *C. aurantium*, one *C. sinensis*, one *C. paradisi*, one *C. limon*, one *C. aurantifolia*, and one *C. micrantha *(additional file 6). The primers and PCR amplification, purification and sequencing were the same as that used for the SNP mining in the candidate genes.

### Study of the origin of unexpected SNPs by Sanger analysis

The origin of unexpected polymorphisms displayed by several SNP markers from the Clementine BES, such as null alleles, no heterozygosity for Clementine and 'fixed heterozygosity', was analyzed by Sanger sequencing of the amplicons of four accessions: 'Nules' Clementine, haploid Clementine, 'Chandler' pummelo and Corsican citron. Primers flanking the SNP site were defined from the contig sequences obtained from the BES [31] to produce amplified fragments ranging from 200 to 620 bp (additional file 7). PCR amplification, purification and sequencing were performed in the same manner as the SNP mining in the candidate genes.

### Data analysis

Neighbor-joining analysis [64] was computed using DARwin software version 5.0 [65]. Genetic dissimilarities were calculated using the simple matching dissimilarity index (d_i-j_) between pairs of accessions:

$$d_{i-j}=1-1∕L\overset{L}{\underset{l=1}{\sum }}m_{l}∕2$$

with d_i-j_, the dissimilarity between units i and j; L, the number of loci; m_l_, the number of matching alleles for locus l. Weighted neighbor-joining trees were computed from the dissimilarity matrix with 1000 bootstraps to test branch robustness. Principal Component Analyses (PCA) were computed using XLSTAT on the matrix of the frequencies for each allele. Genetic population parameters (Ho, observed heterozygosity; He, expected heterozygosity equivalent to Nei diversity index [66]; and Fstats - Fis, Fit and Fst - based on the parameters of Wright [67] and Weir & Cockerham [68] were calculated with GENETIX v. 4.03 software.

For each locus with null alleles, the genotypic diversity (GD) was estimated as

$$GD=1-\overset{G}{\underset{l=1}{\sum }}g_{i}^{2}$$

with G indicating the total number of observed genotypes and g_i _indicating the frequency of each observed genotype.

Linkage disequilibrium (LD) was estimated by r^2 ^(chi square Pearson's correlation coefficient). The significances were estimated with the exac-test pvalue using PowerMarker software v. 3.25 [69].

## Authors' contributions

PO has managed the work, analyzed the data and wrote the paper. JT and MT have generated the Clementine BES data, selected the SNPs from the Clementine BES and contributed to the data validation. AGL, CL and RM have identified SNPs in the candidate genes. DB, AB and AC have developed the GoldenGate array and the SNP genotyping. YF has developed the Chandler × Clementine population and provided DNA. LN has provided most of the germplasm and contributed to the writing of the paper. All authors read and approved the final manuscript.

## Supplementary Material

Additional file 1 **Information on SNP markers included in the GoldenGate array**. This file contains the main information on the SNP markers included in the GoldenGate array. It includes GenBank accession number, sequence surrounding the SNPs, SNP position, GoldenGate primers and designability rank, genotyping code, identification of the loci used for the diversity study. Click here for file

Additional file 2 **Primers for SNP mining in candidate genes and polymorphism results for 7 genotypes representative of 4 basic *Citrus *taxa**. This file contains the main information on the primers used for SNP mining in candidate gene sequences (GenBank accession number, primer sequences, annealing temperature and theoretical amplicon size from EST data) and result data (size of exploitable sequence, number and frequency of SNPs). Click here for file

Additional file 3 **Detailed diversity results for loci without null allele (WONA)**. This file contains main data on the results obtained with WONA loci. It includes heterozygosity in Clementine, observed and theoretical heterozygosity in the whole population and each species, Fstat parameters in the whole population and between and within the three main species (*C. reticulata, C. medica, C. maxima*) and structuration level between *C. reticulata *and *C. maxima*. Click here for file

Additional file 4 **Additional figures**. This file contains two figures. Figure S1: Correlation between the contribution of SNPs loci to the first axis of PCA analysis (*C. maxima *and *C. reticulata *as active individuals) and the Fst values for *C. maxima/C. reticulata *differentiation. Figure S2: Co-distribution of LD between loci without null allele (WONA) pairs for Chandler × Clementine progeny and germplasm population Click here for file

Additional file 5 **Detailed diversity results for loci with null allele (WNA)**. This file contains main data on the results obtained with WNA loci. It includes (i) SNP heterozygosity and heterozygosity for null allele in Clementine, (ii) number of individual in heterozygosity (SNPs), homozygous for one SNP allele and homozygous for null allele in the whole population and within the different species, (iii) genotypic diversity in the whole population (PIC). Click here for file

Additional file 6 **List of germplasm analyzed**. This file contains the list of citrus germplasm accession analyzed. It includes the gerplasm bank, the accession number, the varietal group, the common name, the Latin name according Swingle and Reece and Tanaka classifications and the use for SNP genotyping validation by sequencing. Click here for file

Additional file 7 **Primers for analyzing the origin of unexpected genotyping in the GoldenGate array**. this file contains the main information on the primers used for analyzing the origin of unexpected genotyping in the GoldenGate array. It includes the locus name, the abnormality type, the primer sequences, annealing temperature and amplicon size. Click here for file
