# Supplementary material for: SNP mining in C. clementina BAC end sequences; transferability in the Citrus genus (Rutaceae), phylogenetic inferences and perspectives for genetic mapping
Source: BMC Genomics. 2012 Jan 10;13:13. doi: 10.1186/1471-2164-13-13 (PMC3320530; doi:10.1186/1471-2164-13-13)
Supplement: Additional file 2 — Primers for SNP mining in candidate genes and polymorphism results for 7 genotypes representative of 4 basic Citrus taxa. This file contains the main information on the primers used for SNP mining in candidate gene sequences (GenBank accession number, primer sequences, annealing temperature and theoretical amplicon size from EST data) and result data (size of exploitable sequence, number and frequency of SNPs). [file 1471-2164-13-13-S2.PDF]

Additional file 2: primers for SNP mining in candidate genes and polymorphism results for 7 genotypes representative of 4 basic *Citrus* taxa

Gene code	Gene	GenBank accession	Primer Sequences	Ta	EST (bp)	EGS (bp)	SNP Nb.	SNP/Kb
**CHI**	Chalcone isomerase	DY263683	F: TTGTTCTGATGGCCTAATGG<br>R: AAAGGCTGTCACCGATGAAT	58	721	647	41	63.4
**TRPA**	Vacuolar citrate/H+ symporter	EF028327	F: GGCGCCACTCCTACCTTCCC<br>R: CGGTCATTGAAGAGTGCTCCCC	60	987	715	18	25.2
**PKF**	Phosphofructokinase	AF095520	F: CGCCGACCTCAGTCCCGTC<br>R: GCTGCACGCCCCATAAGCCG	60	807	630	15	23.8
**PSY**	Phytoene synthase	AB037975	F: GCTCGTTGATGGGCCTAATGC<br>R: CGGGCGTAAGAGGGATTTTGC	58	727	560	19	33.9
**LCY2**	Lycopene β-cyclase	FJ516403	F: GCATGGCAACTCTTCTTAGCCCG<br>R: AGCTCGCAAGTAAGGCTCATTCCC	60	850	725	23	31.7
**LCYB**	Lycopene β-cyclase	AY166796	F: GAATTCTTGCCCCAAGTTCA<br>R: TATGGGCCACAAATCTTTCC	58	1206	710	21	29.6
**AOC**	Ascorbate oxydase	DY293375	F : TCAGTGAGAACCCTAAAGC<br>R : CAGTACAACCCCAGTAAGC	55	801	696	15	21.6
**HKT1**	High-affinity K+ Transporter 1	DY297409	F : GTCCATGGAGAAAAAGAACC<br>R : TGCTAGTGTCCGTGAAGAAG	58	641	863	34	39.4
**TS**	Trehalose-6-Phosphate Synthase	FC875388	F : TGCAGAACCTGTAATGAAGC<br>R : CTGGTAGGATGCCGACTTAG	55	505	567	10	17.6
**CAX1**	Cation/H+ membran antiporter	DY278781	F : GTTGCTGATGCTACAGATG<br>R : CCTCTCTCTCTTCTTTACCG	50	805	840	8	9.5
**Total**						**6953**	**204**	**29.3**
