# Supplementary material for: SNP mining in C. clementina BAC end sequences; transferability in the Citrus genus (Rutaceae), phylogenetic inferences and perspectives for genetic mapping
Source: BMC Genomics. 2012 Jan 10;13:13. doi: 10.1186/1471-2164-13-13 (PMC3320530; doi:10.1186/1471-2164-13-13)
Supplement: Additional file 3 — Detailed diversity results for loci without null allele (WONA). This file contains main data on the results obtained with WONA loci. It includes heterozygosity in Clementine, observed and theoretical heterozygosity in the whole population and each species, Fstat parameters in the whole population and between and within the three main species (C. reticulata, C. medica, C. maxima) and structuration level between C. reticulata and C. maxima. [file 1471-2164-13-13-S3.PDF]

Additional file 3: Detailed diversity results for WONA loci

	Clem.	total population			*C. maxima*		*C. medica*		*C. reticulata*		*C. aurantifolia*		*C. aurantium*		*C. limon*		*C. paradisi*		*C.sinensis*		Papeda		3 basic taxa Fstat			Md/Pum dif.	
Locus	He	He	Ho	Fis	Ho	He	Ho	He	Ho	He	Ho	He	Ho	He	Ho	He	Ho	He	Ho	He	Ho	He	Fis	Fit	Fst	Cos^2 F1	Fst
CiC0004-01	1	0.32	0.41	-0.25	0.50	0.38	0.00	0.00	0.75	0.47	0.00	0.00	0.00	0.00	0.14	0.13	1.00	0.50	1.00	0.50	0.00	0.00	-0.45	-0.28	0.12	0.06	0.01
CiC0004-05	1	0.14	0.15	-0.07	0.00	0.00	0.00	0.00	0.33	0.28	0.00	0.00	1.00	0.50	0.14	0.13	0.00	0.00	0.00	0.00	0.00	0.00	-0.14	-0.02	0.11	0.19	0.12
CiC0019-01	1	0.44	0.37	0.18	0.00	0.00	0.00	0.00	0.33	0.28	0.50	0.38	1.00	0.50	0.86	0.49	0.00	0.00	1.00	0.50	0.20	0.18	-0.14	0.78	0.81	**0.88**	0.81
CiC0019-05	1	0.45	0.35	0.23	0.00	0.00	0.00	0.00	0.33	0.28	0.50	0.38	1.00	0.50	0.86	0.49	0.00	0.00	1.00	0.50	0.00	0.32	-0.14	0.78	0.81	**0.88**	0.81
CiC0021-01	1	0.20	0.22	-0.12	0.00	0.00	0.00	0.00	0.42	0.33	0.00	0.00	0.00	0.00	0.14	0.13	0.00	0.00	1.00	0.50	0.20	0.18	-0.21	-0.03	0.15	**0.31**	0.16
CiC0023-03	1	0.48	0.22	0.55	0.00	0.00	0.00	0.00	0.50	0.38	0.50	0.38	1.00	0.50	0.00	0.00	0.00	0.00	0.00	0.00	0.00	0.00	-0.28	0.68	0.75	**0.83**	0.72
CiC0023-07	1	0.32	0.26	0.21	0.00	0.00	0.00	0.00	0.50	0.49	0.00	0.00	1.00	0.50	0.57	0.41	0.00	0.00	0.00	0.00	0.00	0.00	0.03	0.54	0.52	**0.59**	0.54
CiC0025-02	1	0.07	0.07	-0.03	0.00	0.00	0.00	0.00	0.17	0.15	0.00	0.00	0.00	0.00	0.00	0.00	0.00	0.00	0.00	0.00	0.20	0.18	-0.03	-0.01	0.02	0.13	0.03
CiC0046-02	1	0.30	0.30	0.03	0.00	0.00	0.00	0.00	0.58	0.50	0.00	0.00	0.00	0.00	0.29	0.24	1.00	0.50	1.00	0.50	0.00	0.00	-0.12	0.33	0.40	**0.47**	0.41
CiC0046-03	1	0.38	0.41	-0.05	0.00	0.00	0.00	0.00	0.58	0.50	0.50	0.38	1.00	0.50	0.71	0.46	0.00	0.00	1.00	0.50	0.20	0.18	-0.12	0.42	0.48	**0.55**	0.50
CiC0054-05	1	0.36	0.39	-0.08	0.00	0.00	0.00	0.00	0.83	0.49	0.00	0.00	1.00	0.50	1.00	0.50	0.00	0.00	0.00	0.00	0.00	0.00	-0.68	0.23	0.54	**0.81**	0.56
CiC0094-02	1	0.44	0.30	0.34	0.00	0.00	0.00	0.00	0.08	0.08	0.25	0.22	1.00	0.50	0.43	0.34	1.00	0.50	1.00	0.50	0.00	0.00	0.02	0.95	0.95	**0.96**	0.95
CiC0100-04	1	0.49	0.37	0.25	0.00	0.00	1.00	0.50	0.50	0.38	0.00	0.38	1.00	0.50	0.14	0.34	1.00	0.50	1.00	0.50	0.20	0.18	-0.40	0.51	0.65	**0.82**	0.72
CiC0145-02	1	0.43	0.48	-0.11	0.70	0.46	0.20	0.50	0.67	0.44	0.50	0.50	0.00	0.00	0.43	0.34	1.00	0.50	0.00	0.00	0.40	0.32	-0.24	-0.26	-0.02	0.00	-0.02
CiC0153-03	1	0.49	0.49	0.01	0.20	0.42	0.80	0.48	0.60	0.48	1.00	0.50	1.00	0.50	0.40	0.48	0.00	0.00	0.00	0.00	0.25	0.22	0.01	0.02	0.01	0.04	-0.04
CiC0158-03	1	0.35	0.44	-0.28	0.00	0.00	0.20	0.18	0.92	0.50	0.00	0.00	1.00	0.50	0.29	0.24	0.00	0.00	1.00	0.50	0.20	0.18	-0.72	-0.11	0.35	**0.79**	0.43
CiC0181-02	1	0.49	0.26	0.46	0.00	0.00	0.40	0.32	0.17	0.15	0.00	0.00	1.00	0.50	0.00	0.49	1.00	0.50	1.00	0.50	0.25	0.47	-0.11	0.79	0.81	**0.94**	0.91
CiC0202-05	1	0.36	0.21	0.43	0.00	0.00	0.00	0.00	0.45	0.35	0.25	0.22	1.00	0.50	0.14	0.34	0.00	0.00	0.00	0.00	0.20	0.18	-0.24	0.68	0.74	**0.73**	0.76
CiC0215-01	1	0.36	0.23	0.36	0.00	0.00	0.67	0.44	0.25	0.50	0.25	0.22	1.00	0.50	0.43	0.46	0.00	0.00	0.00	0.00	0.00	0.00	0.37	0.55	0.29	**0.37**	0.39
CiC0245-11	1	0.49	0.25	0.49	0.00	0.00	0.60	0.42	0.00	0.00	0.00	0.38	1.00	0.50	0.00	0.48	1.00	0.50	1.00	0.50	0.20	0.18	-0.38	0.85	0.89	**0.97**	1.00
CiC0251-03	1	0.47	0.39	0.18	0.00	0.00	0.00	0.00	0.17	0.15	0.50	0.38	1.00	0.50	0.86	0.49	1.00	0.50	1.00	0.50	0.20	0.18	-0.03	0.90	0.90	**0.94**	0.91
CiC0264-01	1	0.42	0.39	0.09	0.00	0.00	0.00	0.00	0.42	0.41	0.25	0.22	1.00	0.50	0.71	0.46	1.00	0.50	1.00	0.50	0.20	0.18	0.05	0.68	0.66	**0.73**	0.67
CiC0264-02	1	0.14	0.15	-0.07	0.20	0.18	0.00	0.00	0.08	0.08	0.00	0.00	0.00	0.00	0.00	0.00	0.00	0.00	1.00	0.50	0.00	0.00	-0.03	-0.05	-0.02	0.04	-0.02
CiC0265-01	1	0.35	0.42	-0.18	0.00	0.00	0.00	0.00	0.45	0.35	0.50	0.38	1.00	0.50	0.86	0.49	1.00	0.50	1.00	0.50	0.00	0.32	-0.24	-0.02	0.17	0.26	0.19
CiC0277-02	1	0.44	0.37	0.18	0.00	0.00	0.00	0.00	0.33	0.28	0.25	0.22	1.00	0.50	0.43	0.34	1.00	0.50	1.00	0.50	0.20	0.18	-0.14	0.78	0.81	**0.82**	0.81
CiC0277-03	1	0.47	0.33	0.29	0.10	0.10	0.00	0.00	0.08	0.08	0.25	0.22	1.00	0.50	0.43	0.34	1.00	0.50	1.00	0.50	0.20	0.18	0.01	0.90	0.90	**0.91**	0.90
CiC0279-03	1	0.44	0.44	0.01	0.00	0.00	0.00	0.00	0.50	0.38	0.50	0.38	1.00	0.50	0.57	0.41	1.00	0.50	1.00	0.50	0.20	0.18	-0.28	0.63	0.71	**0.75**	0.72
CiC0281-10	1	0.35	0.19	0.47	0.20	0.32	0.00	0.00	0.17	0.15	0.00	0.00	0.00	0.00	0.00	0.00	0.00	0.00	1.00	0.50	0.20	0.18	0.26	0.75	0.66	**0.67**	0.67
CiC0281-12	1	0.09	0.09	-0.04	0.00	0.00	0.00	0.00	0.33	0.28	0.00	0.00	0.00	0.00	0.00	0.00	0.00	0.00	0.00	0.00	0.00	0.00	-0.14	-0.02	0.11	0.17	0.12
CiC0301-02	1	0.27	0.31	-0.18	0.00	0.00	0.00	0.00	0.83	0.49	0.00	0.00	0.00	0.00	0.29	0.24	0.00	0.00	1.00	0.50	0.00	0.00	-0.68	-0.05	0.37	**0.67**	0.39
CiC0345-03	1	0.24	0.21	0.16	0.00	0.00	0.00	0.00	0.45	0.48	0.00	0.00	0.00	0.00	0.14	0.13	0.00	0.00	1.00	0.50	0.00	0.00	0.12	0.42	0.34	**0.32**	0.36
CiC0368-01	1	0.50	0.40	0.22	0.22	0.20	0.00	0.00	0.42	0.47	0.25	0.22	0.00	0.00	0.71	0.46	0.00	0.00	1.00	0.50	0.20	0.42	0.12	0.55	0.49	0.09	0.12
CiC0368-05	1	0.41	0.36	0.14	0.00	0.00	0.00	0.00	0.58	0.50	0.25	0.22	1.00	0.50	0.57	0.41	0.00	0.00	1.00	0.50	0.00	0.38	-0.12	0.42	0.48	**0.62**	0.50
CiC0391-01	1	0.28	0.33	-0.19	0.00	0.00	0.00	0.00	0.75	0.47	0.25	0.22	0.00	0.00	0.29	0.24	0.00	0.00	1.00	0.50	0.20	0.18	-0.56	-0.05	0.33	**0.68**	0.34
CiC0402-02	1	0.50	0.35	0.30	0.00	0.00	0.00	0.00	0.08	0.08	1.00	0.50	1.00	0.50	1.00	0.50	0.00	0.00	1.00	0.50	0.00	0.32	0.02	0.95	0.95	**0.96**	0.95
CiC0402-04	1	0.47	0.37	0.22	0.10	0.10	0.00	0.00	0.17	0.15	0.50	0.38	1.00	0.50	1.00	0.50	0.00	0.00	1.00	0.50	0.20	0.18	-0.02	0.84	0.85	**0.89**	0.85
CiC0446-01	1	0.41	0.35	0.15	0.00	0.00	0.00	0.00	0.42	0.41	0.00	0.00	1.00	0.50	0.57	0.41	1.00	0.50	1.00	0.50	0.00	0.00	0.05	0.68	0.66	**0.69**	0.67
CiC0446-02	1	0.15	0.13	0.16	0.00	0.00	0.00	0.00	0.50	0.44	0.00	0.00	0.00	0.00	0.00	0.00	0.00	0.00	0.00	0.00	0.00	0.00	-0.07	0.22	0.27	**0.35**	0.28
CiC0461-04	1	0.41	0.31	0.24	0.00	0.00	0.00	0.00	0.42	0.41	0.00	0.00	1.00	0.50	1.00	0.50	0.00	0.00	0.00	0.00	0.40	0.48	0.05	0.68	0.66	**0.72**	0.67
CiC0461-05	1	0.41	0.30	0.27	0.00	0.00	0.00	0.00	0.42	0.41	0.00	0.00	1.00	0.50	1.00	0.50	0.00	0.00	0.00	0.00	0.20	0.42	0.05	0.67	0.65	**0.69**	0.66
CiC0466-01	1	0.20	0.22	-0.12	0.00	0.00	0.00	0.00	0.50	0.38	0.00	0.00	0.00	0.00	0.00	0.00	0.00	0.00	1.00	0.50	0.20	0.18	-0.28	-0.03	0.19	**0.37**	0.21
CiC0475-07	1	0.40	0.26	0.36	0.20	0.18	0.00	0.00	0.25	0.22	0.25	0.22	1.00	0.50	0.29	0.24	1.00	0.50	0.00	0.00	0.00	0.00	-0.07	0.72	0.74	**0.71**	0.74
CiC0502-01	1	0.20	0.22	-0.12	0.00	0.00	0.00	0.00	0.08	0.08	0.00	0.00	1.00	0.50	0.14	0.13	0.00	0.00	1.00	0.50	0.20	0.18	0.02	-0.01	-0.02	0.02	-0.01

CiC0521-01	1	0.43	0.41	0.07	0.00	0.00	0.00	0.00	0.50	0.38	0.50	0.38	1.00	0.50	0.43	0.34	1.00	0.50	1.00	0.50	0.20	0.18	-0.28	0.63	0.71	**0.82**	0.72
CiC0528-05	1	0.45	0.30	0.34	0.00	0.00	0.00	0.00	0.42	0.33	0.25	0.22	1.00	0.50	0.43	0.50	0.00	0.00	1.00	0.50	0.00	0.38	-0.21	0.71	0.76	**0.83**	0.77
CiC0550-10	1	0.29	0.31	-0.08	0.10	0.10	0.00	0.00	0.42	0.33	0.00	0.38	1.00	0.50	0.29	0.24	0.00	0.00	1.00	0.50	0.20	0.18	-0.17	-0.08	0.08	0.17	0.07
CiC0561-04	1	0.36	0.47	-0.30	0.00	0.00	0.00	0.00	0.92	0.50	0.00	0.00	1.00	0.50	0.57	0.41	0.00	0.00	1.00	0.50	0.20	0.18	-0.83	-0.06	0.42	**0.75**	0.43
CiC0580-05	1	0.41	0.42	-0.01	0.00	0.00	0.00	0.00	0.58	0.47	0.00	0.00	1.00	0.50	0.57	0.41	1.00	0.50	1.00	0.50	0.20	0.18	-0.19	0.49	0.57	**0.62**	0.58
CiC0580-06	1	0.45	0.28	0.39	0.10	0.10	0.00	0.00	0.50	0.44	0.25	0.47	1.00	0.50	0.43	0.34	0.00	0.00	0.00	0.00	0.00	0.00	-0.06	0.60	0.63	**0.58**	0.55
CiC0593-01	1	0.10	0.11	-0.05	0.00	0.00	0.00	0.00	0.08	0.08	0.00	0.00	0.00	0.00	0.00	0.00	0.00	0.00	1.00	0.50	0.00	0.00	0.02	-0.01	-0.02	0.02	-0.01
CiC0598-01	1	0.29	0.24	0.18	0.00	0.00	0.00	0.00	0.25	0.47	0.00	0.00	0.00	0.00	0.29	0.24	1.00	0.50	1.00	0.50	0.20	0.18	0.51	0.65	0.28	0.31	0.31
CiC0599-01	1	0.27	0.31	-0.18	0.00	0.00	0.00	0.00	0.67	0.44	0.00	0.00	0.00	0.00	0.14	0.13	1.00	0.50	1.00	0.50	0.20	0.18	-0.45	-0.04	0.28	**0.45**	0.30
CiC0604-01	1	0.36	0.46	-0.29	0.00	0.00	0.00	0.00	0.75	0.47	0.50	0.38	1.00	0.50	0.86	0.49	0.00	0.00	1.00	0.50	0.20	0.18	-0.56	-0.05	0.33	**0.65**	0.34
CiC0607-01	1	0.20	0.22	-0.12	0.10	0.10	0.00	0.00	0.58	0.41	0.25	0.22	0.00	0.00	0.29	0.24	0.00	0.00	0.00	0.00	0.00	0.00	-0.30	-0.09	0.16	0.26	0.15
CiC0610-01	1	0.36	0.35	0.02	0.10	0.10	0.00	0.00	0.67	0.49	0.25	0.22	0.00	0.00	0.29	0.24	1.00	0.50	1.00	0.50	0.00	0.00	-0.27	0.32	0.46	**0.61**	0.46
CiC0640-03	1	0.20	0.19	0.07	0.00	0.00	0.00	0.00	0.42	0.33	0.25	0.22	0.00	0.00	0.14	0.13	0.00	0.00	0.00	0.00	0.40	0.48	-0.21	-0.03	0.15	**0.27**	0.16
CiC0701-01	1	0.40	0.37	0.09	0.00	0.00	0.00	0.00	0.58	0.41	0.50	0.38	1.00	0.50	1.00	0.50	0.00	0.00	0.00	0.00	0.20	0.18	-0.36	0.55	0.67	**0.81**	0.68
CiC0701-07	1	0.48	0.22	0.55	0.00	0.00	0.00	0.00	0.50	0.38	0.00	0.00	1.00	0.50	0.14	0.13	0.00	0.00	0.00	0.00	0.00	0.00	-0.28	0.68	0.75	**0.83**	0.72
CiC0717-04	1	0.29	0.32	-0.08	0.00	0.00	0.75	0.47	0.08	0.08	0.25	0.22	1.00	0.50	0.29	0.24	1.00	0.50	1.00	0.50	0.00	0.00	-0.36	0.05	0.30	0.02	-0.01
CiC0748-01	1	0.14	0.15	-0.07	0.00	0.00	0.00	0.00	0.08	0.08	0.00	0.00	0.00	0.00	0.14	0.13	0.00	0.00	1.00	0.50	0.20	0.18	0.02	-0.01	-0.02	0.06	-0.01
CiC0748-02	1	0.15	0.17	-0.08	0.00	0.00	0.00	0.00	0.17	0.15	0.00	0.00	0.00	0.00	0.14	0.13	0.00	0.00	1.00	0.50	0.20	0.18	-0.03	-0.01	0.02	0.08	0.03
CiC0749-04	1	0.12	0.13	-0.06	0.10	0.10	0.00	0.00	0.33	0.28	0.00	0.00	0.00	0.00	0.00	0.00	0.00	0.00	0.00	0.00	0.25	0.22	-0.11	-0.07	0.04	0.07	0.03
CiC0765-01	1	0.46	0.41	0.12	0.00	0.00	0.00	0.00	0.42	0.33	0.25	0.22	1.00	0.50	0.86	0.49	1.00	0.50	1.00	0.50	0.20	0.42	-0.21	0.71	0.76	**0.82**	0.77
CiC0765-03	1	0.46	0.44	0.04	0.10	0.10	0.00	0.00	0.50	0.38	0.25	0.22	1.00	0.50	0.86	0.49	1.00	0.50	1.00	0.50	0.20	0.42	-0.23	0.57	0.65	**0.73**	0.65
CiC0771-01	1	0.45	0.36	0.22	0.11	0.10	0.20	0.18	0.42	0.33	0.00	0.38	1.00	0.50	0.29	0.24	1.00	0.50	1.00	0.50	0.00	0.00	-0.15	0.59	0.64	**0.65**	0.69
CiC0771-03	1	0.44	0.43	0.04	0.00	0.00	0.00	0.00	0.50	0.38	0.25	0.22	1.00	0.50	0.71	0.46	1.00	0.50	1.00	0.50	0.20	0.18	-0.28	0.63	0.71	**0.77**	0.72
CiC0773-01	1	0.41	0.43	-0.03	0.20	0.18	0.00	0.00	0.67	0.44	0.50	0.38	1.00	0.50	1.00	0.50	0.00	0.00	0.00	0.00	0.20	0.18	-0.35	0.33	0.50	**0.65**	0.48
CiC0776-06	1	0.37	0.42	-0.11	0.00	0.00	0.00	0.00	0.73	0.50	0.25	0.22	1.00	0.50	0.57	0.41	0.00	0.00	1.00	0.50	0.00	0.32	-0.42	0.16	0.41	**0.66**	0.43
CiC0780-01	1	0.15	0.17	-0.08	0.00	0.00	0.00	0.00	0.17	0.15	0.00	0.00	1.00	0.50	0.43	0.34	0.00	0.00	0.00	0.00	0.00	0.00	-0.03	-0.01	0.02	0.13	0.03
CiC0784-01	1	0.38	0.06	0.85	0.00	0.00	0.00	0.00	0.00	0.00	0.25	0.22	0.00	0.00	0.14	0.13	0.00	0.00	0.00	0.00	0.00	0.00	1.00	1.00	1.00	**0.97**	1.00
CiC0800-01	1	0.23	0.26	-0.14	0.10	0.10	0.00	0.00	0.00	0.00	0.25	0.22	1.00	0.50	0.43	0.34	0.00	0.00	1.00	0.50	0.00	0.00	0.01	0.00	-0.01	0.05	0.01
CiC0843-01	1	0.21	0.24	-0.13	0.40	0.32	0.80	0.48	0.00	0.00	0.00	0.00	0.00	0.00	0.00	0.00	0.00	0.00	1.00	0.50	0.00	0.00	-0.38	-0.06	0.23	**0.25**	0.19
CiC0851-12	1	0.42	0.22	0.47	0.00	0.00	0.00	0.00	0.08	0.08	0.00	0.00	1.00	0.50	0.14	0.13	1.00	0.50	1.00	0.50	0.00	0.00	0.02	0.95	0.95	**0.96**	0.95
CiC0868-01	1	0.34	0.20	0.40	0.30	0.38	0.00	0.00	0.08	0.08	0.00	0.00	0.00	0.00	0.00	0.00	1.00	0.50	1.00	0.50	0.00	0.00	0.21	0.73	0.67	**0.68**	0.68
CiC0868-02	1	0.15	0.17	-0.08	0.20	0.18	0.00	0.00	0.00	0.00	0.00	0.00	0.00	0.00	0.00	0.00	1.00	0.50	1.00	0.50	0.00	0.00	-0.05	0.00	0.05	0.12	0.07
CiC0892-01	1	0.10	0.11	-0.05	0.00	0.00	0.00	0.00	0.33	0.28	0.00	0.00	0.00	0.00	0.00	0.00	0.00	0.00	0.00	0.00	0.20	0.18	-0.14	-0.02	0.11	0.23	0.12
CiC0892-02	1	0.20	0.22	-0.12	0.00	0.00	0.00	0.00	0.42	0.33	0.00	0.00	0.00	0.00	0.00	0.00	1.00	0.50	1.00	0.50	0.00	0.00	-0.21	-0.03	0.15	0.28	0.16
CiC0908-03	1	0.45	0.38	0.16	0.00	0.00	0.00	0.00	0.42	0.33	0.50	0.38	1.00	0.50	0.71	0.50	0.00	0.00	1.00	0.50	0.20	0.18	-0.20	0.70	0.75	**0.82**	0.76
CiC0928-03	1	0.41	0.39	0.06	0.00	0.00	0.00	0.00	0.50	0.44	0.00	0.00	1.00	0.50	0.57	0.41	1.00	0.50	1.00	0.50	0.20	0.18	-0.07	0.59	0.62	**0.66**	0.63
CiC0928-06	1	0.41	0.39	0.06	0.00	0.00	0.00	0.00	0.50	0.44	0.00	0.00	1.00	0.50	0.57	0.41	1.00	0.50	1.00	0.50	0.20	0.18	-0.07	0.59	0.62	**0.66**	0.63
CiC0948-01	1	0.28	0.33	-0.19	0.00	0.00	0.00	0.00	0.75	0.47	0.25	0.22	0.00	0.00	0.14	0.13	1.00	0.50	1.00	0.50	0.00	0.00	-0.56	-0.05	0.33	**0.55**	0.34
CiC0948-05	1	0.49	0.33	0.34	0.10	0.10	0.00	0.00	0.42	0.33	0.25	0.47	1.00	0.50	0.00	0.50	1.00	0.50	1.00	0.50	0.00	0.38	-0.17	0.65	0.70	**0.73**	0.70
CiC0977-02	1	0.44	0.24	0.46	0.20	0.42	0.00	0.00	0.33	0.28	0.00	0.00	0.00	0.00	0.00	0.00	1.00	0.50	1.00	0.50	0.00	0.00	0.26	0.65	0.52	**0.40**	0.42
CiC1014-03	1	0.49	0.24	0.52	0.00	0.00	0.00	0.00	0.67	0.44	0.00	0.00	1.00	0.50	0.00	0.00	0.00	0.00	0.00	0.00	0.00	0.32	-0.45	0.56	0.70	**0.80**	0.64
CiC1041-01	1	0.12	0.09	0.24	0.00	0.00	0.00	0.00	0.33	0.38	0.00	0.00	0.00	0.00	0.00	0.00	0.00	0.00	0.00	0.00	0.00	0.00	0.17	0.31	0.16	0.19	0.18
CiC1067-02	1	0.37	0.22	0.40	0.30	0.26	0.00	0.00	0.17	0.15	0.00	0.00	0.00	0.00	0.00	0.00	1.00	0.50	1.00	0.50	0.00	0.00	-0.08	0.70	0.73	**0.81**	0.74
CiC1119-03	1	0.12	0.12	-0.06	0.00	0.00	0.00	0.00	0.42	0.33	0.00	0.00	0.00	0.00	0.00	0.00	0.00	0.00	0.00	0.00	0.00	0.00	-0.21	-0.03	0.15	0.29	0.16
CiC1123-01	1	0.06	0.06	-0.02	0.00	0.00	0.00	0.00	0.17	0.15	0.00	0.00	0.00	0.00	0.00	0.00	0.00	0.00	0.00	0.00	0.00	0.00	-0.03	-0.02	0.01	0.12	0.03
CiC1123-02	1	0.05	0.06	-0.02	0.00	0.00	0.00	0.00	0.17	0.15	0.00	0.00	0.00	0.00	0.00	0.00	0.00	0.00	0.00	0.00	0.00	0.00	-0.03	-0.01	0.02	0.13	0.03
CiC1135-01	1	0.36	0.35	0.02	0.40	0.50	0.00	0.00	0.00	0.00	0.50	0.38	1.00	0.50	0.43	0.34	0.00	0.00	1.00	0.50	0.20	0.18	0.26	0.60	0.46	**0.47**	0.50

CiC1155-01	1	0.05	0.06	-0.02	0.00	0.00	0.00	0.00	0.17	0.15	0.00	0.00	0.00	0.00	0.00	0.00	0.00	0.00	0.00	0.00	0.00	0.00	-0.03	-0.01	0.02	0.13	0.03
CiC1203-01	1	0.09	0.09	-0.04	0.10	0.10	0.00	0.00	0.25	0.22	0.00	0.00	0.00	0.00	0.00	0.00	0.00	0.00	0.00	0.00	0.00	0.00	-0.06	-0.06	0.00	0.03	-0.01
CiC1208-01	1	0.21	0.17	0.22	0.10	0.26	0.00	0.00	0.08	0.08	0.25	0.22	0.00	0.00	0.14	0.13	0.00	0.00	1.00	0.50	0.00	0.32	0.48	0.47	-0.01	0.03	0.00
CiC1208-08	1	0.14	0.15	-0.07	0.00	0.00	0.00	0.00	0.08	0.08	0.25	0.22	0.00	0.00	0.14	0.13	0.00	0.00	1.00	0.50	0.00	0.00	0.02	-0.01	-0.02	0.08	-0.01
CiC1209-02	1	0.29	0.31	-0.07	0.00	0.00	0.00	0.00	0.50	0.44	0.33	0.28	0.00	0.00	0.29	0.24	0.00	0.00	1.00	0.50	0.20	0.18	-0.06	0.21	0.26	**0.34**	0.27
CiC1209-04	1	0.48	0.44	0.07	0.10	0.10	0.00	0.00	0.58	0.41	0.75	0.47	0.00	0.00	0.71	0.46	0.00	0.00	1.00	0.50	0.20	0.42	-0.30	0.49	0.60	**0.27**	0.15
CiC1229-01	1	0.29	0.25	0.18	0.10	0.10	0.75	0.47	0.33	0.49	0.00	0.00	0.00	0.00	0.00	0.00	0.00	0.00	1.00	0.50	0.00	0.00	0.14	0.28	0.17	**0.31**	0.26
CiC1229-05	1	0.40	0.41	-0.01	0.00	0.00	0.20	0.18	0.58	0.47	0.25	0.22	1.00	0.50	0.71	0.46	0.00	0.00	1.00	0.50	0.20	0.18	-0.17	0.43	0.51	**0.74**	0.59
CiC1231-05	1	0.18	0.20	-0.10	0.00	0.00	0.00	0.00	0.42	0.33	0.25	0.22	0.00	0.00	0.00	0.00	0.00	0.00	1.00	0.50	0.00	0.00	-0.21	-0.03	0.15	0.21	0.16
CiC1251-02	1	0.49	0.14	0.73	0.00	0.18	0.00	0.00	0.00	0.00	0.00	0.38	1.00	0.50	0.00	0.49	0.00	0.00	1.00	0.50	0.00	0.00	1.00	1.00	0.90	**0.82**	0.90
CiC1260-01	1	0.05	0.06	-0.02	0.00	0.00	0.00	0.00	0.17	0.15	0.00	0.00	0.00	0.00	0.00	0.00	0.00	0.00	0.00	0.00	0.00	0.00	-0.03	-0.01	0.02	0.09	0.03
CiC1313-02	1	0.40	0.21	0.48	0.00	0.00	0.00	0.00	0.09	0.35	0.00	0.00	1.00	0.50	0.14	0.13	1.00	0.50	1.00	0.50	0.00	0.38	0.77	0.94	0.73	**0.67**	0.74
CiC1315-03	1	0.38	0.28	0.27	0.50	0.46	0.00	0.00	0.33	0.28	0.00	0.00	0.00	0.00	0.14	0.13	0.00	0.00	1.00	0.50	0.00	0.00	-0.08	0.35	0.40	**0.47**	0.37
CiC1331-02	1	0.42	0.37	0.12	0.10	0.10	0.00	0.00	0.50	0.38	0.50	0.38	1.00	0.50	1.00	0.50	0.00	0.00	0.00	0.00	0.20	0.18	-0.23	0.57	0.65	**0.76**	0.65
CiC1422-01	1	0.45	0.34	0.25	0.00	0.00	0.00	0.00	0.33	0.28	0.25	0.22	1.00	0.50	0.67	0.50	0.00	0.00	1.00	0.50	0.20	0.18	-0.14	0.78	0.81	**0.88**	0.81
CiC1425-03	1	0.46	0.37	0.20	0.00	0.00	0.00	0.00	0.25	0.22	0.50	0.38	1.00	0.50	1.00	0.50	0.00	0.00	1.00	0.50	0.20	0.18	-0.08	0.84	0.85	**0.92**	0.86
CiC1426-01	1	0.32	0.30	0.10	0.00	0.00	0.00	0.00	0.75	0.47	0.25	0.22	0.00	0.00	0.14	0.13	0.00	0.00	1.00	0.50	0.00	0.00	-0.56	0.35	0.58	**0.74**	0.60
CiC1426-06	1	0.48	0.37	0.23	0.00	0.00	0.00	0.00	0.17	0.15	0.50	0.38	1.00	0.50	1.00	0.50	0.00	0.00	1.00	0.50	0.40	0.48	-0.03	0.90	0.90	**0.94**	0.91
CiC1428-01	1	0.38	0.41	-0.05	0.00	0.00	0.00	0.00	0.67	0.50	0.50	0.38	1.00	0.50	0.00	0.24	1.00	0.50	1.00	0.50	0.20	0.18	-0.28	0.29	0.45	**0.54**	0.46
CiC1428-05	1	0.44	0.33	0.26	0.00	0.00	0.00	0.00	0.25	0.22	0.50	0.38	1.00	0.50	0.14	0.13	1.00	0.50	1.00	0.50	0.20	0.18	-0.08	0.84	0.85	**0.86**	0.86
CiC1436-01	1	0.50	0.39	0.22	0.00	0.00	0.00	0.00	0.17	0.15	0.75	0.47	1.00	0.50	0.86	0.49	1.00	0.50	1.00	0.50	0.00	0.48	-0.03	0.90	0.90	**0.95**	0.91
CiC1441-01	1	0.47	0.37	0.22	0.00	0.00	0.00	0.00	0.17	0.15	0.50	0.38	1.00	0.50	1.00	0.50	0.00	0.00	1.00	0.50	0.40	0.32	-0.03	0.90	0.90	**0.94**	0.91
CiC1441-05	1	0.49	0.41	0.18	0.50	0.46	0.40	0.32	0.08	0.08	1.00	0.50	1.00	0.50	0.43	0.34	0.00	0.00	1.00	0.50	0.00	0.32	-0.07	0.51	0.55	**0.62**	0.58
CiC1444-03	1	0.41	0.25	0.42	0.00	0.00	0.00	0.00	0.17	0.15	0.00	0.00	1.00	0.50	0.14	0.13	1.00	0.50	1.00	0.50	0.00	0.00	-0.03	0.89	0.89	**0.90**	0.90
CiC1447-07	1	0.46	0.49	-0.06	0.00	0.00	0.00	0.00	0.75	0.47	0.25	0.22	0.00	0.00	1.00	0.50	1.00	0.50	1.00	0.50	0.20	0.42	-0.56	0.34	0.58	**0.71**	0.58
CiC1453-01	1	0.23	0.22	0.03	0.00	0.00	0.00	0.00	0.50	0.44	0.00	0.00	0.00	0.00	0.00	0.00	0.00	0.00	1.00	0.50	0.20	0.18	-0.07	0.22	0.27	**0.38**	0.28
CiC1459-02	1	0.47	0.37	0.22	0.00	0.00	0.00	0.00	0.17	0.15	0.25	0.22	1.00	0.50	1.00	0.50	1.00	0.50	1.00	0.50	0.20	0.18	-0.03	0.90	0.90	**0.95**	0.91
CiC1459-03	1	0.21	0.17	0.22	0.40	0.48	0.00	0.00	0.00	0.00	0.00	0.00	0.00	0.00	0.00	0.00	0.00	0.00	1.00	0.50	0.00	0.00	0.22	0.50	0.35	**0.38**	0.39
CiC1504-01	1	0.40	0.56	-0.38	0.00	0.00	0.00	0.00	1.00	0.50	0.50	0.38	1.00	0.50	0.57	0.41	1.00	0.50	1.00	0.50	0.20	0.18	-1.00	-0.06	0.47	**0.97**	0.48
CiC1505-02	1	0.07	0.07	-0.03	0.00	0.00	0.00	0.00	0.25	0.22	0.00	0.00	0.00	0.00	0.00	0.00	0.00	0.00	0.00	0.00	0.00	0.00	-0.08	-0.02	0.06	0.16	0.08
CiC1516-02	1	0.24	0.24	0.00	0.50	0.46	0.00	0.00	0.08	0.08	0.00	0.00	0.00	0.00	0.00	0.00	1.00	0.50	1.00	0.50	0.00	0.00	-0.03	0.21	0.23	**0.31**	0.24
CiC1525-01	1	0.12	0.13	-0.06	0.00	0.00	0.00	0.00	0.18	0.17	0.00	0.00	0.00	0.00	0.00	0.00	0.00	0.00	1.00	0.50	0.00	0.00	-0.04	-0.01	0.03	0.17	0.04
CiC1528-01	1	0.21	0.24	-0.13	0.00	0.00	0.00	0.00	0.42	0.33	0.00	0.00	0.00	0.00	0.14	0.13	1.00	0.50	1.00	0.50	0.00	0.00	-0.21	-0.03	0.15	0.17	0.16
CiC1550-02	1	0.20	0.22	-0.12	0.00	0.00	0.00	0.00	0.67	0.44	0.25	0.22	0.00	0.00	0.29	0.24	0.00	0.00	0.00	0.00	0.00	0.00	-0.45	-0.04	0.28	**0.56**	0.30
CiC1564-03	1	0.24	0.27	-0.15	0.67	0.44	0.00	0.00	0.08	0.08	0.00	0.00	0.00	0.00	0.00	0.00	1.00	0.50	1.00	0.50	0.00	0.00	-0.36	-0.05	0.23	**0.38**	0.24
CiC1564-06	1	0.49	0.37	0.26	0.00	0.00	0.80	0.48	0.50	0.38	0.25	0.22	1.00	0.50	0.29	0.24	0.00	0.00	1.00	0.50	0.00	0.00	-0.40	0.41	0.58	**0.86**	0.72
CiC1596-01	1	0.49	0.50	-0.01	0.00	0.00	0.00	0.00	0.50	0.38	1.00	0.50	1.00	0.50	1.00	0.50	1.00	0.50	1.00	0.50	0.00	0.32	-0.28	0.63	0.71	**0.77**	0.72
CiC1638-01	1	0.37	0.37	0.00	0.00	0.00	1.00	0.50	0.50	0.38	0.00	0.00	0.00	0.00	0.00	0.49	1.00	0.50	1.00	0.50	0.40	0.32	-0.53	-0.13	0.26	**0.38**	0.21
CiC1646-07	1	0.05	0.06	-0.02	0.00	0.00	0.00	0.00	0.17	0.15	0.00	0.00	0.00	0.00	0.00	0.00	0.00	0.00	0.00	0.00	0.00	0.00	-0.03	-0.01	0.02	0.13	0.03
CiC1655-02	1	0.41	0.39	0.06	0.00	0.00	0.00	0.00	0.50	0.44	0.00	0.00	1.00	0.50	0.57	0.41	1.00	0.50	1.00	0.50	0.00	0.00	-0.07	0.59	0.62	**0.64**	0.63
CiC1663-05	1	0.09	0.09	-0.04	0.00	0.00	0.00	0.00	0.33	0.28	0.00	0.00	0.00	0.00	0.00	0.00	0.00	0.00	0.00	0.00	0.00	0.00	-0.14	-0.02	0.11	0.17	0.12
CiC1702-02	1	0.14	0.11	0.20	0.00	0.00	0.00	0.00	0.17	0.28	0.00	0.00	0.00	0.00	0.29	0.24	0.00	0.00	0.00	0.00	0.20	0.18	0.45	0.49	0.07	0.11	0.10
CiC1723-01	1	0.10	0.11	-0.05	0.00	0.00	0.00	0.00	0.17	0.15	0.00	0.00	0.00	0.00	0.29	0.24	0.00	0.00	0.00	0.00	0.20	0.18	-0.03	-0.01	0.02	0.11	0.03
CiC1749-05	1	0.29	0.28	0.05	0.00	0.00	0.00	0.00	0.75	0.50	0.00	0.00	1.00	0.50	0.43	0.34	0.00	0.00	0.00	0.00	0.00	0.38	-0.47	0.13	0.41	**0.71**	0.42
CiC1757-02	1	0.47	0.37	0.22	0.00	0.00	0.00	0.00	0.17	0.15	0.50	0.38	1.00	0.50	0.57	0.41	1.00	0.50	1.00	0.50	0.20	0.18	-0.03	0.90	0.90	**0.90**	0.91
CiC1757-04	1	0.31	0.35	-0.11	0.70	0.50	0.00	0.00	0.08	0.08	0.25	0.22	1.00	0.50	0.43	0.34	0.00	0.00	1.00	0.50	0.00	0.00	-0.30	0.15	0.35	**0.49**	0.36
CiC1766-09	1	0.46	0.52	-0.12	0.22	0.35	0.50	0.38	0.25	0.22	0.75	0.47	0.00	0.00	1.00	0.50	0.00	0.00	1.00	0.50	0.60	0.42	0.10	0.37	0.30	0.04	-0.03

CiC1811-02	1	0.24	0.24	0.00	0.50	0.46	0.00	0.00	0.17	0.15	0.00	0.00	0.00	0.00	0.14	0.13	0.00	0.00	1.00	0.50	0.00	0.00	-0.04	0.14	0.17	**0.26**	0.16
CiC1862-01	1	0.49	0.41	0.18	0.00	0.00	0.00	0.00	0.92	0.50	0.25	0.22	1.00	0.50	0.14	0.13	0.00	0.00	1.00	0.50	0.00	0.00	-0.83	0.37	0.66	**0.87**	0.52
CiC1868-02	1	0.29	0.13	0.56	0.20	0.42	0.00	0.00	0.00	0.00	0.00	0.00	0.00	0.00	0.00	0.00	0.00	0.00	1.00	0.50	0.00	0.00	0.57	0.86	0.67	**0.62**	0.70
CiC1868-04	1	0.47	0.34	0.29	0.10	0.10	0.00	0.00	0.08	0.08	0.25	0.22	1.00	0.50	0.57	0.41	1.00	0.50	1.00	0.50	0.40	0.32	0.01	0.90	0.90	**0.90**	0.90
CiC1873-02	1	0.44	0.39	0.12	0.00	0.00	0.00	0.00	0.42	0.33	0.50	0.38	1.00	0.50	0.86	0.49	0.00	0.00	1.00	0.50	0.20	0.18	-0.21	0.71	0.76	**0.87**	0.77
CiC1876-03	1	0.27	0.24	0.10	0.00	0.00	0.00	0.00	0.50	0.49	0.00	0.00	1.00	0.50	0.43	0.34	0.00	0.00	0.00	0.00	0.00	0.00	0.03	0.37	0.35	**0.41**	0.37
CiC1881-01	1	0.41	0.35	0.15	0.00	0.00	0.00	0.00	0.50	0.38	0.50	0.38	1.00	0.50	1.00	0.50	0.00	0.00	0.00	0.00	0.20	0.18	-0.28	0.63	0.71	**0.83**	0.72
CiC1881-03	1	0.48	0.22	0.55	0.00	0.00	0.00	0.00	0.50	0.38	0.00	0.00	1.00	0.50	0.00	0.00	0.00	0.00	0.00	0.00	0.20	0.18	-0.28	0.68	0.75	**0.83**	0.72
CiC1891-01	1	0.45	0.31	0.31	0.00	0.00	0.00	0.00	0.17	0.15	0.25	0.22	1.00	0.50	0.57	0.41	1.00	0.50	1.00	0.50	0.20	0.18	-0.03	0.90	0.90	**0.94**	0.91
CiC1891-02	1	0.45	0.31	0.31	0.00	0.00	0.00	0.00	0.17	0.15	0.25	0.22	1.00	0.50	0.57	0.41	1.00	0.50	1.00	0.50	0.20	0.18	-0.03	0.90	0.90	**0.94**	0.91
CiC1897-03	1	0.30	0.30	0.03	0.00	0.00	0.00	0.00	0.42	0.41	0.00	0.00	0.00	0.00	0.43	0.34	1.00	0.50	1.00	0.50	0.20	0.42	0.05	0.26	0.22	**0.32**	0.24
CiC1931-04	1	0.23	0.22	0.03	0.00	0.00	0.00	0.00	0.67	0.49	0.25	0.22	0.00	0.00	0.29	0.24	0.00	0.00	0.00	0.00	0.00	0.00	-0.32	0.16	0.36	**0.64**	0.38
CiC1945-01	1	0.24	0.24	0.00	0.50	0.46	0.00	0.00	0.08	0.08	0.00	0.00	0.00	0.00	0.00	0.00	1.00	0.50	1.00	0.50	0.00	0.00	-0.03	0.21	0.23	**0.31**	0.24
CiC1998-02	1	0.44	0.33	0.26	0.10	0.10	0.00	0.00	0.08	0.08	0.50	0.38	1.00	0.50	0.14	0.13	1.00	0.50	1.00	0.50	0.40	0.32	0.01	0.89	0.89	**0.91**	0.90
CiC1998-10	1	0.47	0.31	0.34	0.00	0.00	0.20	0.18	0.08	0.08	0.75	0.47	1.00	0.50	0.00	0.00	1.00	0.50	1.00	0.50	0.20	0.50	-0.02	0.90	0.90	**0.96**	0.95
CiC2010-01	1	0.47	0.25	0.48	0.00	0.00	0.00	0.00	0.00	0.00	0.00	0.38	1.00	0.50	0.50	0.38	1.00	0.50	1.00	0.50	0.20	0.18	1.00	1.00	1.00	**0.97**	1.00
CiC2011-02	1	0.49	0.40	0.20	0.43	0.34	1.00	0.50	0.42	0.33	0.00	0.38	1.00	0.50	0.00	0.41	1.00	0.50	0.00	0.00	0.50	0.38	-0.42	0.05	0.33	**0.38**	0.48
CiC2011-05	1	0.20	0.19	0.07	0.20	0.32	0.00	0.00	0.42	0.33	0.00	0.00	0.00	0.00	0.00	0.00	1.00	0.50	0.00	0.00	0.00	0.00	0.08	0.09	0.01	0.00	-0.05
CiC2093-02	1	0.47	0.41	0.14	0.10	0.10	1.00	0.50	0.58	0.47	0.00	0.00	1.00	0.50	0.00	0.49	1.00	0.50	0.75	0.47	0.00	0.00	-0.38	0.13	0.37	**0.58**	0.51
CiC2093-03	1	0.39	0.39	0.02	0.00	0.00	0.00	0.00	0.58	0.47	0.00	0.00	1.00	0.50	0.57	0.41	1.00	0.50	1.00	0.50	0.00	0.00	-0.19	0.50	0.58	**0.67**	0.59
CiC2110-01	1	0.35	0.26	0.26	0.00	0.00	0.00	0.00	0.25	0.33	0.00	0.00	1.00	0.50	0.57	0.41	1.00	0.50	0.00	0.00	0.20	0.18	0.30	0.38	0.12	0.14	0.14
CiC2128-01	1	0.45	0.50	-0.10	0.00	0.00	0.00	0.00	0.58	0.41	0.50	0.38	1.00	0.50	1.00	0.50	1.00	0.50	1.00	0.50	0.20	0.18	-0.36	0.55	0.67	**0.77**	0.68
CiC2151-03	1	0.40	0.19	0.55	0.00	0.00	0.00	0.00	0.00	0.00	0.25	0.22	1.00	0.50	0.00	0.00	1.00	0.50	1.00	0.50	0.00	0.00	1.00	1.00	1.00	**0.97**	1.00
CiC2373-01	1	0.43	0.30	0.31	0.00	0.00	0.25	0.22	0.42	0.41	0.75	0.47	0.00	0.00	0.29	0.24	0.00	0.00	0.00	0.00	0.50	0.38	0.03	0.54	0.52	0.26	0.24
CiC2401-02	1	0.25	0.25	-0.01	0.11	0.10	1.00	0.50	0.33	0.28	0.25	0.47	0.00	0.00	0.00	0.00	0.00	0.00	0.00	0.00	0.50	0.38	-0.38	-0.12	0.19	0.08	0.02
CiC2401-05	1	0.46	0.48	-0.05	0.00	0.00	0.00	0.00	0.50	0.38	0.50	0.38	1.00	0.50	1.00	0.50	1.00	0.50	1.00	0.50	0.20	0.18	-0.28	0.63	0.71	**0.83**	0.72
CiC2406-01	1	0.46	0.44	0.04	0.00	0.00	0.00	0.00	0.42	0.33	0.50	0.38	1.00	0.50	1.00	0.50	1.00	0.50	1.00	0.50	0.20	0.18	-0.21	0.71	0.76	**0.82**	0.77
CiC2414-01	1	0.23	0.26	-0.14	0.00	0.00	0.00	0.00	0.50	0.38	0.25	0.22	0.00	0.00	0.00	0.00	1.00	0.50	1.00	0.50	0.00	0.00	-0.28	-0.03	0.19	**0.32**	0.21
CiC2417-04	1	0.43	0.19	0.58	0.00	0.00	0.00	0.00	0.08	0.08	0.25	0.22	1.00	0.50	0.00	0.00	0.00	0.00	1.00	0.50	0.00	0.00	0.02	0.95	0.95	**0.96**	0.95
CiC2424-01	1	0.31	0.28	0.12	0.00	0.00	0.00	0.00	0.75	0.50	0.25	0.22	0.00	0.00	0.43	0.34	0.00	0.00	0.00	0.00	0.20	0.18	-0.47	0.13	0.41	**0.69**	0.42
CiC2424-02	1	0.23	0.15	0.35	0.00	0.00	0.00	0.00	0.00	0.00	0.50	0.38	0.00	0.00	0.00	0.00	0.00	0.00	1.00	0.50	0.20	0.42	M	M		**0.00**	
CiC2434-01	1	0.38	0.41	-0.05	0.00	0.00	0.00	0.00	0.58	0.50	0.00	0.00	1.00	0.50	0.57	0.41	1.00	0.50	1.00	0.50	0.20	0.18	-0.12	0.42	0.48	**0.55**	0.50
CiC2506-01	1	0.05	0.06	-0.02	0.00	0.00	0.00	0.00	0.17	0.15	0.00	0.00	0.00	0.00	0.00	0.00	0.00	0.00	0.00	0.00	0.00	0.00	-0.03	-0.01	0.02	0.13	0.03
CiC2510-03	1	0.45	0.35	0.23	0.00	0.00	0.00	0.00	0.25	0.22	0.25	0.22	1.00	0.50	0.86	0.49	1.00	0.50	1.00	0.50	0.00	0.00	-0.08	0.84	0.85	**0.89**	0.86
CiC2510-08	1	0.44	0.24	0.46	0.00	0.00	0.00	0.00	0.08	0.08	0.25	0.22	1.00	0.50	0.00	0.00	1.00	0.50	1.00	0.50	0.00	0.32	0.02	0.95	0.95	**0.96**	0.95
CiC2518-02	1	0.48	0.41	0.15	0.00	0.00	0.00	0.00	0.33	0.28	0.25	0.47	0.00	0.00	0.86	0.49	1.00	0.50	1.00	0.50	0.40	0.48	-0.14	0.72	0.76	0.13	0.12
CiC2518-04	1	0.49	0.31	0.38	0.00	0.00	0.00	0.00	0.33	0.28	0.25	0.47	0.00	0.00	0.86	0.49	1.00	0.50	0.00	0.00	0.00	0.44	-0.14	0.72	0.76	0.13	0.12
CiC2524-01	1	0.48	0.37	0.24	0.00	0.00	0.00	0.00	0.50	0.44	0.25	0.47	1.00	0.50	0.43	0.34	1.00	0.50	1.00	0.50	0.00	0.00	-0.07	0.67	0.69	**0.66**	0.63
CiC2524-04	1	0.49	0.53	-0.08	0.00	0.00	0.80	0.48	0.50	0.44	0.50	0.38	1.00	0.50	0.83	0.49	1.00	0.50	1.00	0.50	0.20	0.42	-0.24	0.35	0.48	**0.66**	0.63
CiC2532-01	1	0.45	0.44	0.03	0.50	0.50	0.00	0.00	0.33	0.38	1.00	0.50	1.00	0.50	0.00	0.00	1.00	0.50	1.00	0.50	0.00	0.50	0.11	0.30	0.22	0.18	0.13
CiC2533-03	1	0.05	0.06	-0.02	0.00	0.00	0.00	0.00	0.17	0.15	0.00	0.00	0.00	0.00	0.00	0.00	0.00	0.00	0.00	0.00	0.00	0.00	-0.03	-0.01	0.02	0.13	0.03
CiC2533-06	1	0.35	0.30	0.15	0.00	0.00	0.00	0.00	0.50	0.50	0.00	0.00	1.00	0.50	0.86	0.49	0.00	0.00	0.00	0.00	0.20	0.18	0.06	0.47	0.43	**0.57**	0.45
CiC2538-01	1	0.25	0.15	0.42	0.20	0.42	0.00	0.00	0.08	0.08	0.00	0.00	0.00	0.00	0.00	0.00	0.00	0.00	1.00	0.50	0.00	0.00	0.47	0.55	0.15	**0.17**	0.17
CiC2612-02	1	0.46	0.59	-0.29	0.20	0.18	1.00	0.50	0.75	0.50	0.75	0.47	1.00	0.50	1.00	0.50	0.00	0.00	0.00	0.00	0.20	0.42	-0.52	-0.15	0.24	**0.52**	0.34
CiC2612-05	1	0.49	0.55	-0.11	0.33	0.28	0.80	0.48	0.75	0.50	0.75	0.47	1.00	0.50	0.71	0.46	0.00	0.00	0.00	0.00	0.00	0.48	-0.42	-0.16	0.18	**0.38**	0.24
CiC2644-01	1	0.49	0.33	0.33	0.00	0.00	0.00	0.00	0.50	0.38	0.75	0.47	0.00	0.00	0.14	0.50	1.00	0.50	1.00	0.50	0.00	0.00	-0.28	0.68	0.75	**0.74**	0.72
CiC2648-01	1	0.27	0.24	0.10	0.00	0.00	0.00	0.00	0.42	0.47	0.25	0.22	0.00	0.00	0.29	0.24	0.00	0.00	1.00	0.50	0.00	0.00	0.17	0.42	0.30	**0.40**	0.32

CiC2648-03	1	0.29	0.35	-0.21	0.00	0.00	0.00	0.00	0.58	0.41	0.00	0.00	1.00	0.50	0.29	0.24	1.00	0.50	1.00	0.50	0.00	0.00	-0.36	-0.04	0.24	**0.35**	0.25
CiC2689-05	1	0.38	0.30	0.24	0.00	0.00	0.00	0.00	0.42	0.41	0.00	0.00	1.00	0.50	1.00	0.50	0.00	0.00	0.00	0.00	0.20	0.18	0.05	0.68	0.66	**0.72**	0.67
CiC2693-01	1	0.31	0.28	0.12	0.00	0.00	0.00	0.00	0.58	0.50	0.25	0.22	0.00	0.00	0.00	0.00	1.00	0.50	1.00	0.50	0.00	0.00	-0.12	0.42	0.48	**0.57**	0.50
CiC2699-01	1	0.39	0.28	0.30	0.50	0.38	0.00	0.00	0.33	0.28	0.00	0.00	0.00	0.00	0.14	0.13	0.00	0.00	1.00	0.50	0.00	0.00	-0.22	0.41	0.52	**0.63**	0.50
CiC2708-04	1	0.40	0.33	0.18	0.00	0.00	0.00	0.00	0.50	0.38	0.00	0.00	1.00	0.50	1.00	0.50	0.00	0.00	0.00	0.00	0.20	0.18	-0.28	0.63	0.71	**0.83**	0.72
CiC2708-08	1	0.48	0.22	0.55	0.00	0.00	0.00	0.00	0.50	0.38	0.50	0.38	1.00	0.50	0.00	0.00	0.00	0.00	0.00	0.00	0.00	0.00	-0.28	0.68	0.75	**0.83**	0.72
CiC2788-09	1	0.06	0.06	-0.02	0.00	0.00	0.00	0.00	0.17	0.15	0.00	0.00	0.00	0.00	0.00	0.00	0.00	0.00	0.00	0.00	0.00	0.00	-0.03	-0.01	0.02	0.13	0.03
CiC2809-02	1	0.37	0.41	-0.09	0.00	0.00	0.00	0.00	0.83	0.49	0.00	0.00	0.00	0.00	0.29	0.24	1.00	0.50	1.00	0.50	0.50	0.38	-0.68	0.23	0.54	**0.73**	0.56
CiC2810-02	1	0.41	0.30	0.27	0.00	0.00	0.00	0.00	0.67	0.49	0.25	0.22	0.00	0.00	0.43	0.34	1.00	0.50	0.00	0.00	0.25	0.22	-0.32	0.39	0.53	**0.68**	0.55
CiC2824-01	1	0.06	0.06	-0.02	0.00	0.00	0.00	0.00	0.17	0.15	0.00	0.00	0.00	0.00	0.00	0.00	0.00	0.00	0.00	0.00	0.00	0.00	-0.03	-0.01	0.02	0.13	0.03
CiC2840-01	1	0.48	0.33	0.31	0.00	0.00	0.00	0.00	0.17	0.15	0.75	0.47	1.00	0.50	0.43	0.34	1.00	0.50	1.00	0.50	0.20	0.50	-0.03	0.90	0.90	**0.88**	0.91
CiC2900-04	1	0.43	0.30	0.32	0.20	0.18	0.00	0.00	0.08	0.08	0.25	0.22	1.00	0.50	0.43	0.46	1.00	0.50	1.00	0.50	0.00	0.00	-0.03	0.83	0.84	**0.86**	0.85
CiC2900-06	1	0.42	0.31	0.27	0.20	0.18	0.00	0.00	0.08	0.08	0.25	0.22	1.00	0.50	0.57	0.41	1.00	0.50	1.00	0.50	0.00	0.00	-0.03	0.83	0.84	**0.86**	0.85
CiC2931-01	1	0.28	0.33	-0.19	0.40	0.32	0.00	0.00	0.25	0.22	0.25	0.22	1.00	0.50	0.29	0.24	0.00	0.00	1.00	0.50	0.00	0.00	-0.14	-0.12	0.02	0.01	-0.02
CiC2931-05	1	0.28	0.33	-0.19	0.40	0.32	0.00	0.00	0.25	0.22	0.25	0.22	1.00	0.50	0.29	0.24	0.00	0.00	1.00	0.50	0.00	0.00	-0.14	-0.12	0.02	0.01	-0.02
CiC2945-01	1	0.16	0.17	-0.09	0.00	0.00	0.20	0.18	0.42	0.33	0.00	0.00	0.00	0.00	0.29	0.24	0.00	0.00	0.00	0.00	0.00	0.00	-0.18	-0.07	0.09	0.21	0.16
CiC2974-01	1	0.32	0.26	0.21	0.10	0.10	0.00	0.00	0.50	0.49	0.00	0.00	0.00	0.00	0.29	0.24	0.00	0.00	0.00	0.00	0.80	0.48	0.03	0.47	0.45	**0.55**	0.45
CiC2985-01	1	0.18	0.13	0.30	0.00	0.00	0.00	0.00	0.25	0.22	0.50	0.38	0.00	0.00	0.00	0.00	0.00	0.00	0.00	0.00	0.20	0.50	-0.08	-0.02	0.06	0.11	0.08
CiC2994-01	1	0.46	0.30	0.36	0.00	0.00	0.00	0.00	0.08	0.08	0.25	0.22	1.00	0.50	0.57	0.41	1.00	0.50	1.00	0.50	0.20	0.18	0.02	0.95	0.95	**0.95**	0.95
CiC3005-01	1	0.50	0.52	-0.03	0.10	0.38	0.80	0.48	0.33	0.28	0.50	0.38	1.00	0.50	0.86	0.49	0.00	0.00	1.00	0.50	0.40	0.48	0.11	0.43	0.36	**0.47**	0.48
CiC3056-02	1	0.44	0.37	0.18	0.30	0.26	0.00	0.00	0.42	0.33	0.00	0.38	1.00	0.50	0.29	0.24	1.00	0.50	1.00	0.50	0.00	0.00	-0.17	0.52	0.59	**0.59**	0.57
CiC3074-02	1	0.44	0.41	0.09	0.00	0.00	0.00	0.00	0.42	0.33	0.50	0.38	1.00	0.50	0.86	0.49	0.00	0.00	1.00	0.50	0.20	0.18	-0.21	0.71	0.76	**0.87**	0.77
CiC3096-02	1	0.45	0.39	0.15	0.00	0.00	0.00	0.00	0.33	0.28	0.50	0.38	1.00	0.50	0.86	0.49	0.00	0.00	1.00	0.50	0.40	0.32	-0.14	0.78	0.81	**0.88**	0.81
CiC3189-10	1	0.24	0.28	-0.16	0.00	0.00	0.20	0.18	0.55	0.40	0.00	0.00	1.00	0.50	0.14	0.13	0.00	0.00	0.00	0.00	0.40	0.32	-0.27	-0.08	0.15	**0.38**	0.24
CiC3202-03	1	0.24	0.28	-0.15	0.00	0.00	0.00	0.00	0.67	0.44	0.00	0.00	1.00	0.50	0.14	0.13	0.00	0.00	0.00	0.00	0.20	0.18	-0.45	-0.04	0.28	**0.52**	0.30
CiC3234-02	1	0.43	0.40	0.09	0.10	0.10	0.00	0.00	0.50	0.38	1.00	0.50	0.00	0.00	1.00	0.50	0.00	0.00	0.00	0.00	0.00	0.38	-0.23	0.54	0.63	**0.23**	0.11
CiC3248-01	1	0.19	0.21	-0.11	0.00	0.00	0.60	0.42	0.08	0.08	0.00	0.00	0.00	0.00	0.17	0.15	0.00	0.00	1.00	0.50	0.20	0.18	-0.25	0.02	0.22	0.02	-0.01
CiC3248-06	1	0.37	0.22	0.40	0.10	0.10	0.00	0.00	0.33	0.49	0.50	0.38	0.00	0.00	0.00	0.00	1.00	0.50	0.00	0.00	0.40	0.32	0.32	0.49	0.26	0.23	0.26
CiC3258-02	1	0.23	0.22	0.03	0.00	0.00	0.00	0.00	0.33	0.38	0.25	0.22	1.00	0.50	0.57	0.41	0.00	0.00	0.00	0.00	0.00	0.00	0.17	0.31	0.17	0.31	0.19
CiC3258-03	1	0.48	0.43	0.12	0.00	0.00	0.00	0.00	0.50	0.38	1.00	0.50	1.00	0.50	0.86	0.49	0.00	0.00	1.00	0.50	0.00	0.32	-0.28	0.63	0.71	**0.82**	0.72
CiC3261-01	1	0.46	0.29	0.37	0.00	0.00	0.00	0.00	0.50	0.44	0.00	0.00	1.00	0.50	0.00	0.49	1.00	0.50	1.00	0.50	0.00	0.00	-0.06	0.59	0.61	**0.64**	0.63
CiC3261-02	1	0.46	0.31	0.35	0.00	0.00	0.25	0.22	0.50	0.44	0.00	0.00	1.00	0.50	0.00	0.49	1.00	0.50	1.00	0.50	0.00	0.00	-0.07	0.52	0.55	**0.64**	0.63
CiC3276-02	1	0.18	0.17	0.10	0.00	0.00	0.20	0.18	0.50	0.44	0.00	0.00	0.00	0.00	0.00	0.00	0.00	0.00	0.00	0.00	0.20	0.18	-0.06	0.15	0.20	**0.36**	0.28
CiC3334-01	1	0.18	0.20	-0.10	0.20	0.18	0.80	0.48	0.25	0.22	0.00	0.00	0.00	0.00	0.14	0.13	0.00	0.00	0.00	0.00	0.00	0.00	-0.26	-0.14	0.10	0.01	-0.04
CiC3334-03	1	0.47	0.44	0.06	0.00	0.00	0.00	0.00	0.33	0.28	0.50	0.38	1.00	0.50	0.71	0.46	1.00	0.50	1.00	0.50	0.60	0.42	-0.14	0.78	0.81	**0.82**	0.81
CiC3352-01	1	0.05	0.06	-0.02	0.00	0.00	0.00	0.00	0.17	0.15	0.00	0.00	0.00	0.00	0.00	0.00	0.00	0.00	0.00	0.00	0.00	0.00	-0.03	-0.01	0.02	0.11	0.03
CiC3394-01	1	0.29	0.31	-0.08	0.00	0.00	0.00	0.00	0.75	0.50	0.00	0.00	0.00	0.00	0.00	0.00	1.00	0.50	1.00	0.50	0.20	0.18	-0.47	0.13	0.41	**0.53**	0.42
CiC3401-01	1	0.44	0.37	0.18	0.00	0.00	0.00	0.00	0.33	0.28	0.50	0.38	1.00	0.50	0.86	0.49	0.00	0.00	1.00	0.50	0.20	0.18	-0.14	0.78	0.81	**0.88**	0.81
CiC3437-02	1	0.15	0.13	0.16	0.30	0.26	0.00	0.00	0.17	0.28	0.25	0.22	0.00	0.00	0.00	0.00	0.00	0.00	0.00	0.00	0.00	0.00	0.21	0.19	-0.02	0.00	-0.06
CiC3437-07	1	0.09	0.09	-0.04	0.00	0.00	0.00	0.00	0.17	0.15	0.00	0.00	0.00	0.00	0.14	0.13	0.00	0.00	0.00	0.00	0.20	0.18	-0.03	-0.01	0.02	0.13	0.03
CiC3440-08	1	0.46	0.34	0.26	0.20	0.18	1.00	0.50	0.67	0.49	0.00	0.00	1.00	0.50	0.00	0.44	0.00	0.00	0.00	0.00	0.25	0.22	-0.37	0.04	0.30	**0.46**	0.38
CiC3448-07	1	0.46	0.49	-0.06	0.00	0.00	0.00	0.00	0.50	0.38	0.50	0.38	1.00	0.50	1.00	0.50	1.00	0.50	1.00	0.50	0.20	0.18	-0.28	0.63	0.71	**0.72**	0.71
CiC3457-01	1	0.48	0.22	0.55	0.00	0.00	0.00	0.00	0.50	0.38	0.25	0.22	1.00	0.50	0.00	0.00	0.00	0.00	0.00	0.00	0.00	0.00	-0.28	0.68	0.75	**0.83**	0.72
CiC3459-03	1	0.09	0.09	-0.04	0.00	0.00	0.25	0.22	0.17	0.15	0.00	0.00	0.00	0.00	0.14	0.13	0.00	0.00	0.00	0.00	0.00	0.00	-0.05	-0.04	0.01	0.09	0.03
CiC3490-01	1	0.14	0.15	-0.07	0.00	0.00	0.00	0.00	0.50	0.38	0.00	0.00	0.00	0.00	0.14	0.13	0.00	0.00	0.00	0.00	0.00	0.00	-0.28	-0.04	0.19	**0.32**	0.20
CiC3518-03	1	0.12	0.13	-0.06	0.10	0.10	0.00	0.00	0.08	0.08	0.00	0.00	0.00	0.00	0.00	0.00	0.00	0.00	1.00	0.50	0.00	0.00	0.01	-0.04	-0.05	0.00	-0.05
CiC3519-01	1	0.25	0.30	-0.17	0.00	0.00	0.00	0.00	0.58	0.41	0.25	0.22	0.00	0.00	0.29	0.24	0.00	0.00	1.00	0.50	0.20	0.18	-0.36	-0.04	0.24	**0.42**	0.25

CiC3519-04	1	0.20	0.08	0.63	0.00	0.00	0.00	0.00	0.18	0.50	0.00	0.00	0.00	0.00	0.14	0.13	0.00	0.00	0.00	0.00	0.00	0.00	0.67	0.79	0.37	**0.30**	0.40
CiC3532-01	1	0.31	0.35	-0.11	0.00	0.00	0.00	0.00	0.58	0.47	0.00	0.00	1.00	0.50	0.43	0.34	0.00	0.00	1.00	0.50	0.20	0.18	-0.19	0.18	0.31	**0.48**	0.33
CiC3532-02	1	0.40	0.41	-0.01	0.00	0.00	0.00	0.00	0.58	0.47	0.25	0.22	1.00	0.50	0.57	0.41	1.00	0.50	1.00	0.50	0.00	0.00	-0.19	0.50	0.58	**0.59**	0.59
CiC3536-01	1	0.34	0.28	0.18	0.00	0.00	0.00	0.00	0.25	0.22	0.25	0.22	1.00	0.50	0.14	0.50	1.00	0.50	1.00	0.50	0.20	0.18	-0.08	-0.02	0.06	0.17	0.08
CiC3546-04	1	0.48	0.33	0.32	0.00	0.00	0.00	0.00	0.17	0.15	0.50	0.38	1.00	0.50	0.57	0.41	0.00	0.00	1.00	0.50	0.20	0.42	-0.03	0.90	0.90	**0.88**	0.91
CiC3573-05	1	0.41	0.19	0.54	0.00	0.00	0.00	0.00	0.58	0.41	0.00	0.00	1.00	0.50	0.00	0.49	0.00	0.00	0.00	0.00	0.00	0.32	-0.36	0.51	0.64	**0.44**	0.25
CiC3653-01	1	0.44	0.39	0.12	0.00	0.00	0.00	0.00	0.42	0.33	0.50	0.38	1.00	0.50	0.86	0.49	0.00	0.00	1.00	0.50	0.20	0.18	-0.21	0.71	0.76	**0.87**	0.77
CiC3653-02	1	0.41	0.46	-0.12	0.00	0.00	0.00	0.00	0.75	0.47	0.50	0.38	1.00	0.50	0.86	0.49	0.00	0.00	1.00	0.50	0.20	0.18	-0.56	0.35	0.58	**0.86**	0.60
CiC3674-01	1	0.38	0.31	0.17	0.00	0.00	0.00	0.00	0.33	0.49	0.25	0.22	0.00	0.00	0.43	0.34	1.00	0.50	1.00	0.50	0.40	0.32	0.37	0.69	0.51	**0.53**	0.53
CiC3674-02	1	0.48	0.39	0.20	0.10	0.10	0.00	0.00	0.17	0.15	0.25	0.47	1.00	0.50	0.86	0.49	1.00	0.50	1.00	0.50	0.20	0.18	-0.02	0.84	0.85	**0.91**	0.85
CiC3712-01	1	0.44	0.37	0.18	0.00	0.00	0.00	0.00	0.33	0.28	0.00	0.00	1.00	0.50	0.86	0.49	0.00	0.00	1.00	0.50	0.20	0.18	-0.14	0.78	0.81	**0.83**	0.81
CiC3712-04	1	0.05	0.06	-0.02	0.00	0.00	0.00	0.00	0.17	0.15	0.00	0.00	0.00	0.00	0.00	0.00	0.00	0.00	0.00	0.00	0.00	0.00	-0.03	-0.01	0.02	0.13	0.03
CiC3739-01	1	0.21	0.20	0.06	0.00	0.00	0.00	0.00	0.33	0.38	0.00	0.00	0.00	0.00	0.00	0.00	1.00	0.50	0.75	0.47	0.00	0.00	0.18	0.30	0.15	0.17	0.19
CiC3739-02	1	0.23	0.22	0.03	0.00	0.00	0.00	0.00	0.50	0.44	0.00	0.00	0.00	0.00	0.00	0.00	1.00	0.50	0.75	0.47	0.00	0.00	-0.07	0.22	0.27	0.28	0.28
CiC3801-02	1	0.44	0.28	0.37	0.00	0.00	0.00	0.00	0.25	0.47	0.25	0.22	1.00	0.50	0.71	0.46	0.00	0.00	0.00	0.00	0.20	0.18	0.51	0.78	0.56	**0.56**	0.58
CiC3801-03	1	0.28	0.34	-0.20	0.00	0.00	0.00	0.00	0.75	0.47	0.25	0.22	0.00	0.00	0.29	0.24	0.00	0.00	1.00	0.50	0.25	0.22	-0.56	-0.05	0.33	**0.68**	0.34
CiC3809-01	1	0.09	0.09	-0.04	0.00	0.00	0.00	0.00	0.00	0.00	0.00	0.00	0.00	0.00	0.00	0.00	0.00	0.00	1.00	0.50	0.00	0.00	M	M		**0.00**	
CiC3873-02	1	0.18	0.20	-0.10	0.20	0.18	0.00	0.00	0.33	0.28	0.00	0.00	0.00	0.00	0.00	0.00	0.00	0.00	1.00	0.50	0.00	0.00	-0.11	-0.10	0.01	0.03	-0.02
CiC3918-10	1	0.15	0.09	0.40	0.10	0.10	0.00	0.00	0.17	0.38	0.00	0.00	0.00	0.00	0.14	0.13	0.00	0.00	0.00	0.00	0.00	0.00	0.50	0.53	0.08	0.13	0.08
CiC3931-01	1	0.39	0.39	0.02	0.00	0.00	0.00	0.00	0.58	0.47	0.00	0.00	1.00	0.50	0.43	0.34	1.00	0.50	1.00	0.50	0.20	0.18	-0.19	0.50	0.58	**0.67**	0.59
CiC3931-04	1	0.40	0.41	-0.01	0.00	0.00	0.00	0.00	0.58	0.47	0.00	0.00	1.00	0.50	0.57	0.41	1.00	0.50	1.00	0.50	0.20	0.18	-0.19	0.50	0.58	**0.67**	0.59
CiC3948-03	1	0.38	0.35	0.07	0.00	0.00	0.00	0.00	0.58	0.47	0.25	0.22	1.00	0.50	0.57	0.41	0.00	0.00	1.00	0.50	0.00	0.00	-0.19	0.50	0.58	**0.70**	0.59
CiC3948-04	1	0.38	0.35	0.07	0.00	0.00	0.00	0.00	0.58	0.47	0.25	0.22	1.00	0.50	0.57	0.41	0.00	0.00	1.00	0.50	0.00	0.00	-0.19	0.50	0.58	**0.70**	0.59
CiC3959-01	1	0.17	0.19	-0.10	0.30	0.26	0.00	0.00	0.17	0.15	0.00	0.00	0.00	0.00	0.00	0.00	0.00	0.00	1.00	0.50	0.00	0.00	-0.08	-0.08	0.00	0.04	-0.02
CiC3965-05	1	0.37	0.44	-0.21	0.00	0.00	0.20	0.18	0.92	0.50	0.25	0.22	1.00	0.50	0.00	0.24	1.00	0.50	1.00	0.50	0.00	0.00	-0.72	-0.11	0.35	**0.87**	0.43
CiC4015-02	1	0.40	0.37	0.09	0.40	0.42	0.00	0.00	0.25	0.41	0.25	0.22	1.00	0.50	0.29	0.24	0.00	0.00	1.00	0.50	0.40	0.32	0.29	0.32	0.04	0.00	-0.06
CiC4015-03	1	0.12	0.13	-0.06	0.10	0.10	0.00	0.00	0.42	0.33	0.00	0.00	0.00	0.00	0.00	0.00	0.00	0.00	0.00	0.00	0.00	0.00	-0.17	-0.08	0.08	0.16	0.07
CiC4033-01	1	0.48	0.30	0.38	0.00	0.00	0.00	0.00	0.00	0.00	0.25	0.22	0.00	0.00	0.71	0.46	1.00	0.50	1.00	0.50	0.25	0.22	1.00	1.00	1.00	**0.00**	
CiC4043-03	1	0.14	0.11	0.20	0.00	0.00	0.40	0.32	0.25	0.33	0.00	0.00	0.00	0.00	0.00	0.00	0.00	0.00	0.00	0.00	0.00	0.00	0.16	0.22	0.07	0.23	0.14
CiC4043-05	1	0.28	0.25	0.09	0.10	0.10	1.00	0.50	0.25	0.41	0.00	0.00	1.00	0.50	0.00	0.00	0.00	0.00	0.00	0.00	0.40	0.32	0.05	0.20	0.16	0.19	0.12
CiC4048-01	1	0.07	0.08	-0.03	0.00	0.00	0.00	0.00	0.25	0.22	0.00	0.00	0.00	0.00	0.00	0.00	0.00	0.00	0.00	0.00	0.00	0.00	-0.08	-0.02	0.06	0.16	0.08
CiC4112-02	1	0.46	0.41	0.12	0.00	0.00	0.00	0.00	0.33	0.28	0.50	0.38	1.00	0.50	0.57	0.41	1.00	0.50	1.00	0.50	0.20	0.18	-0.14	0.78	0.81	**0.82**	0.81
CiC4112-05	1	0.45	0.32	0.30	0.00	0.00	0.00	0.00	0.08	0.08	0.25	0.22	1.00	0.50	0.50	0.38	1.00	0.50	1.00	0.50	0.60	0.42	0.02	0.95	0.95	**0.95**	0.95
CiC4120-01	1	0.09	0.09	-0.04	0.00	0.00	0.00	0.00	0.25	0.22	0.00	0.00	0.00	0.00	0.00	0.00	0.00	0.00	0.00	0.00	0.20	0.18	-0.08	-0.02	0.06	0.15	0.08
CiC4122-11	1	0.38	0.46	-0.23	0.40	0.32	0.40	0.32	0.50	0.38	0.25	0.47	1.00	0.50	0.14	0.13	1.00	0.50	1.00	0.50	0.00	0.00	-0.24	-0.29	-0.04	0.01	-0.03
CiC4125-03	1	0.31	0.30	0.02	0.20	0.18	0.00	0.00	0.08	0.08	0.25	0.22	1.00	0.50	0.43	0.34	0.00	0.00	1.00	0.50	0.00	0.00	-0.03	-0.05	-0.02	0.04	-0.02
CiC4152-02	1	0.44	0.22	0.51	0.00	0.00	0.00	0.00	0.17	0.15	0.25	0.22	1.00	0.50	0.00	0.00	0.00	0.00	1.00	0.50	0.00	0.00	-0.03	0.90	0.90	**0.94**	0.91
CiC4152-10	1	0.25	0.22	0.13	0.60	0.42	0.00	0.00	0.08	0.08	0.00	0.00	0.00	0.00	0.00	0.00	0.00	0.00	1.00	0.50	0.00	0.00	-0.30	-0.06	0.19	**0.32**	0.20
CiC4155-01	1	0.46	0.39	0.17	0.00	0.00	0.00	0.00	0.17	0.28	0.75	0.47	1.00	0.50	0.71	0.46	1.00	0.50	1.00	0.50	0.20	0.18	0.45	0.89	0.80	**0.82**	0.81
CiC4175-06	1	0.46	0.31	0.33	0.00	0.00	0.00	0.00	0.33	0.28	0.25	0.47	0.00	0.00	0.71	0.46	1.00	0.50	1.00	0.50	0.00	0.00	-0.14	0.72	0.76	0.13	0.12
CiC4209-02	1	0.47	0.39	0.18	0.00	0.00	0.00	0.00	0.17	0.15	0.25	0.22	1.00	0.50	1.00	0.50	1.00	0.50	1.00	0.50	0.40	0.32	-0.03	0.90	0.90	**0.95**	0.91
CiC4225-01	1	0.09	0.09	-0.04	0.00	0.00	0.00	0.00	0.33	0.28	0.00	0.00	0.00	0.00	0.00	0.00	0.00	0.00	0.00	0.00	0.00	0.00	-0.14	-0.02	0.11	**0.29**	0.12
CiC4240-04	1	0.32	0.30	0.10	0.40	0.50	0.00	0.00	0.08	0.08	0.00	0.00	1.00	0.50	0.57	0.41	0.00	0.00	1.00	0.50	0.00	0.00	0.22	0.52	0.38	**0.40**	0.40
CiC4240-08	1	0.47	0.35	0.26	0.00	0.00	0.00	0.00	0.08	0.08	0.50	0.38	1.00	0.50	0.57	0.41	1.00	0.50	1.00	0.50	0.20	0.18	0.02	0.95	0.95	**0.95**	0.95
CiC4244-02	1	0.46	0.24	0.49	0.00	0.00	0.00	0.00	0.33	0.28	0.25	0.22	1.00	0.50	0.14	0.13	0.00	0.00	1.00	0.50	0.00	0.00	-0.14	0.79	0.82	**0.88**	0.81
CiC4303-01	1	0.39	0.28	0.30	0.00	0.00	0.00	0.00	0.33	0.38	0.00	0.00	1.00	0.50	0.29	0.24	0.00	0.00	1.00	0.50	0.00	0.00	0.17	0.76	0.71	**0.69**	0.72
CiC4310-01	1	0.42	0.30	0.30	0.00	0.00	0.20	0.18	0.25	0.33	0.25	0.22	0.00	0.00	0.29	0.24	1.00	0.50	1.00	0.50	0.40	0.32	0.23	0.77	0.69	**0.79**	0.76

CiC4310-05	1	0.10	0.11	-0.05	0.10	0.10	0.00	0.00	0.00	0.00	0.00	0.00	0.00	0.00	0.00	0.00	0.00	0.00	1.00	0.50	0.00	0.00	0.01	0.00	-0.01	0.05	0.01
CiC4338-01	1	0.09	0.10	-0.04	0.00	0.00	0.00	0.00	0.00	0.00	0.00	0.00	0.00	0.00	0.00	0.00	0.00	0.00	1.00	0.50	0.00	0.00	M	M		**0.00**	
CiC4356-06	1	0.40	0.36	0.11	0.00	0.00	0.20	0.18	0.00	0.00	0.75	0.47	0.00	0.00	1.00	0.50	1.00	0.50	1.00	0.50	0.00	0.00	-0.05	0.91	0.92	**0.00**	
CiC4359-02	1	0.25	0.30	-0.17	0.00	0.00	0.00	0.00	0.50	0.38	0.50	0.38	1.00	0.50	0.57	0.41	0.00	0.00	0.00	0.00	0.00	0.00	-0.28	-0.03	0.19	**0.33**	0.21
CiC4370-01	1	0.15	0.17	-0.08	0.50	0.38	0.00	0.00	0.25	0.22	0.00	0.00	0.00	0.00	0.00	0.00	0.00	0.00	0.00	0.00	0.00	0.00	-0.20	-0.13	0.05	0.05	0.02
CiC4370-02	1	0.44	0.30	0.34	0.00	0.00	0.00	0.00	0.25	0.22	0.25	0.22	1.00	0.50	0.29	0.24	1.00	0.50	1.00	0.50	0.00	0.00	-0.08	0.85	0.86	**0.82**	0.86
CiC4383-01	1	0.10	0.11	-0.05	0.10	0.10	0.00	0.00	0.00	0.00	0.00	0.00	0.00	0.00	0.00	0.00	0.00	0.00	1.00	0.50	0.00	0.00	0.01	0.00	-0.01	0.06	0.01
CiC4385-01	1	0.28	0.26	0.08	0.10	0.10	0.00	0.00	0.08	0.08	0.25	0.22	1.00	0.50	0.29	0.24	1.00	0.50	1.00	0.50	0.00	0.00	0.01	-0.04	-0.05	0.00	-0.05
CiC4385-07	1	0.40	0.43	-0.06	0.22	0.20	0.00	0.00	0.70	0.46	0.00	0.00	1.00	0.50	0.71	0.46	0.00	0.00	0.75	0.47	0.25	0.22	-0.37	0.28	0.47	**0.61**	0.45
CiC4406-02	1	0.49	0.26	0.48	0.00	0.00	0.00	0.00	0.50	0.44	0.50	0.38	1.00	0.50	0.00	0.00	0.00	0.00	0.00	0.00	0.20	0.18	-0.07	0.67	0.69	**0.67**	0.63
CiC4415-01	1	0.41	0.39	0.06	0.00	0.00	0.00	0.00	0.50	0.44	0.00	0.00	1.00	0.50	0.57	0.41	1.00	0.50	1.00	0.50	0.00	0.00	-0.07	0.59	0.62	**0.64**	0.63
CiC4440-01	1	0.07	0.07	-0.03	0.10	0.10	0.00	0.00	0.17	0.15	0.00	0.00	0.00	0.00	0.00	0.00	0.00	0.00	0.00	0.00	0.00	0.00	-0.02	-0.05	-0.03	0.03	-0.04
CiC4440-03	1	0.11	0.11	-0.05	0.10	0.10	0.00	0.00	0.17	0.15	0.25	0.22	0.00	0.00	0.14	0.13	0.00	0.00	0.00	0.00	0.00	0.00	-0.02	-0.05	-0.03	0.03	-0.04
CiC4465-08	1	0.21	0.24	-0.13	0.10	0.10	0.00	0.00	0.33	0.28	0.00	0.00	0.00	0.00	0.14	0.13	1.00	0.50	1.00	0.50	0.00	0.00	-0.11	-0.07	0.04	0.05	0.03
CiC4473-01	1	0.43	0.48	-0.11	0.10	0.10	0.00	0.00	0.50	0.49	0.50	0.38	1.00	0.50	1.00	0.50	1.00	0.50	1.00	0.50	0.00	0.00	0.03	0.47	0.45	**0.47**	0.45
CiC4510-02	1	0.21	0.24	-0.13	0.10	0.10	0.20	0.18	0.08	0.08	0.25	0.22	0.00	0.00	0.14	0.13	1.00	0.50	1.00	0.50	0.20	0.18	-0.01	-0.06	-0.05	0.00	-0.05
CiC4542-01	1	0.29	0.31	-0.08	0.00	0.00	0.00	0.00	0.50	0.44	0.00	0.00	1.00	0.50	0.14	0.13	0.00	0.00	1.00	0.50	0.20	0.18	-0.07	0.22	0.27	**0.25**	0.28
CiC4570-01	1	0.20	0.22	-0.12	0.00	0.00	0.00	0.00	0.33	0.28	0.00	0.00	0.00	0.00	0.14	0.13	1.00	0.50	1.00	0.50	0.00	0.00	-0.14	-0.02	0.11	0.13	0.12
CiC4581-01	1	0.29	0.28	0.05	0.00	0.00	0.00	0.00	0.58	0.50	0.25	0.22	0.00	0.00	0.29	0.24	0.00	0.00	1.00	0.50	0.20	0.18	-0.12	0.33	0.40	**0.59**	0.41
CiC4598-01	1	0.07	0.07	-0.03	0.00	0.00	0.00	0.00	0.25	0.22	0.00	0.00	0.00	0.00	0.00	0.00	0.00	0.00	0.00	0.00	0.00	0.00	-0.08	-0.02	0.06	0.20	0.08
CiC4613-01	1	0.47	0.35	0.26	0.00	0.00	0.00	0.00	0.08	0.08	0.25	0.22	1.00	0.50	1.00	0.50	1.00	0.50	1.00	0.50	0.20	0.18	0.02	0.95	0.95	**0.96**	0.95
CiC4613-02	1	0.18	0.20	-0.10	0.00	0.00	0.20	0.18	0.42	0.33	0.00	0.00	0.00	0.00	0.43	0.34	0.00	0.00	0.00	0.00	0.20	0.18	-0.18	-0.07	0.09	**0.28**	0.16
CiC4614-01	1	0.05	0.06	-0.02	0.00	0.00	0.00	0.00	0.17	0.15	0.00	0.00	0.00	0.00	0.00	0.00	0.00	0.00	0.00	0.00	0.00	0.00	-0.03	-0.01	0.02	0.10	0.03
CiC4616-01	1	0.27	0.31	-0.18	0.10	0.10	0.00	0.00	0.58	0.41	0.25	0.22	0.00	0.00	0.29	0.24	0.00	0.00	1.00	0.50	0.20	0.18	-0.30	-0.09	0.16	**0.28**	0.15
CiC4620-07	1	0.07	0.07	-0.03	0.00	0.00	0.00	0.00	0.17	0.15	0.00	0.00	0.00	0.00	0.14	0.13	0.00	0.00	0.00	0.00	0.00	0.00	-0.03	-0.01	0.02	0.10	0.03
CiC4643-02	1	0.25	0.30	-0.17	0.00	0.00	0.00	0.00	0.58	0.41	0.25	0.22	0.00	0.00	0.29	0.24	0.00	0.00	1.00	0.50	0.20	0.18	-0.36	-0.04	0.24	**0.42**	0.25
CiC4652-02	1	0.38	0.28	0.26	0.00	0.50	0.00	0.00	0.00	0.15	0.50	0.38	1.00	0.50	0.50	0.38	0.00	0.00	1.00	0.50	0.20	0.18	1.00	1.00	0.28	**0.24**	0.29
CiC4681-02	1	0.12	0.09	0.24	0.00	0.00	0.20	0.18	0.27	0.35	0.00	0.00	0.00	0.00	0.00	0.00	0.00	0.00	0.00	0.00	0.00	0.00	0.22	0.29	0.09	0.18	0.17
CiC4681-05	1	0.17	0.12	0.35	0.00	0.00	0.00	0.00	0.36	0.46	0.00	0.00	0.00	0.00	0.00	0.00	0.00	0.00	0.00	0.00	0.20	0.18	0.27	0.48	0.28	0.24	0.30
CiC4687-04	1	0.34	0.35	-0.04	0.00	0.00	0.00	0.00	0.67	0.50	0.00	0.00	1.00	0.50	0.14	0.13	1.00	0.50	1.00	0.50	0.00	0.00	-0.28	0.29	0.45	**0.62**	0.46
CiC4689-02	1	0.07	0.07	-0.03	0.00	0.00	0.00	0.00	0.25	0.22	0.00	0.00	0.00	0.00	0.00	0.00	0.00	0.00	0.00	0.00	0.00	0.00	-0.08	-0.02	0.06	0.17	0.08
CiC4689-04	1	0.50	0.44	0.12	0.00	0.00	1.00	0.50	0.50	0.38	0.75	0.47	1.00	0.50	0.00	0.49	1.00	0.50	1.00	0.50	0.20	0.42	-0.53	0.35	0.57	**0.72**	0.72
CiC4714-01	1	0.20	0.22	-0.12	0.00	0.00	0.00	0.00	0.33	0.28	0.00	0.00	0.00	0.00	0.14	0.13	1.00	0.50	1.00	0.50	0.00	0.00	-0.14	-0.02	0.11	0.13	0.12
CiC4717-02	1	0.38	0.30	0.24	0.00	0.00	0.00	0.00	0.42	0.41	0.00	0.00	1.00	0.50	1.00	0.50	0.00	0.00	0.00	0.00	0.20	0.18	0.05	0.68	0.66	**0.72**	0.67
CiC4717-04	1	0.47	0.28	0.42	0.10	0.10	1.00	0.50	0.42	0.41	0.00	0.00	1.00	0.50	0.00	0.00	0.00	0.00	0.00	0.00	0.20	0.18	-0.25	0.31	0.45	**0.66**	0.60
CiC4747-02	1	0.30	0.26	0.15	0.40	0.50	0.00	0.00	0.42	0.33	0.00	0.00	0.00	0.00	0.00	0.00	0.00	0.00	1.00	0.50	0.00	0.00	0.05	0.24	0.20	0.14	0.13
CiC4747-06	1	0.15	0.17	-0.08	0.00	0.00	0.00	0.00	0.17	0.15	0.00	0.00	0.00	0.00	0.00	0.00	1.00	0.50	1.00	0.50	0.00	0.00	-0.03	-0.01	0.02	0.10	0.03
CiC4770-01	1	0.37	0.26	0.30	0.00	0.00	0.20	0.18	0.33	0.44	0.00	0.38	1.00	0.50	0.00	0.49	1.00	0.50	1.00	0.50	0.00	0.00	0.25	0.39	0.18	**0.28**	0.27
CiC4790-01	1	0.18	0.20	-0.10	0.10	0.10	0.00	0.00	0.42	0.33	0.25	0.22	0.00	0.00	0.14	0.13	0.00	0.00	0.00	0.00	0.20	0.18	-0.17	-0.08	0.08	0.16	0.07
CiC4790-02	1	0.12	0.13	-0.06	0.10	0.10	0.00	0.00	0.00	0.00	0.00	0.00	0.00	0.00	0.00	0.00	0.00	0.00	1.00	0.50	0.20	0.18	0.01	0.00	-0.01	0.05	0.01
CiC4796-02	1	0.50	0.43	0.15	0.00	0.00	1.00	0.50	0.50	0.38	0.25	0.47	1.00	0.50	0.43	0.34	0.00	0.00	1.00	0.50	0.20	0.42	-0.53	0.35	0.57	**0.86**	0.72
CiC4827-01	1	0.20	0.22	-0.12	0.00	0.00	0.00	0.00	0.08	0.08	0.00	0.00	1.00	0.50	0.43	0.34	0.00	0.00	1.00	0.50	0.00	0.00	0.02	-0.01	-0.02	0.05	-0.01
CiC4831-01	1	0.43	0.26	0.41	0.00	0.00	0.00	0.00	0.25	0.33	0.25	0.22	1.00	0.50	0.29	0.49	0.00	0.00	1.00	0.50	0.20	0.18	0.30	0.83	0.75	**0.79**	0.76
CiC4831-03	1	0.50	0.18	0.65	0.10	0.10	0.00	0.00	0.08	0.08	0.25	0.47	1.00	0.50	0.00	0.41	0.00	0.00	0.75	0.47	0.00	0.00	0.02	0.90	0.90	**0.91**	0.90
CiC4853-01	1	0.20	0.23	-0.12	0.20	0.18	0.00	0.00	0.17	0.15	0.00	0.00	0.00	0.00	0.00	0.00	1.00	0.50	1.00	0.50	0.25	0.22	-0.04	-0.07	-0.03	0.00	-0.04
CiC4857-03	1	0.29	0.31	-0.08	0.00	0.00	0.00	0.00	0.58	0.47	0.00	0.00	1.00	0.50	0.43	0.34	0.00	0.00	1.00	0.50	0.00	0.00	-0.19	0.18	0.31	**0.44**	0.33
CiC4858-01	1	0.17	0.15	0.13	0.00	0.00	0.00	0.00	0.58	0.47	0.00	0.00	0.00	0.00	0.00	0.00	0.00	0.00	0.00	0.00	0.00	0.00	-0.19	0.18	0.31	**0.50**	0.33

CiC4876-02	1	0.48	0.41	0.15	0.00	0.00	0.00	0.00	0.17	0.15	0.50	0.38	1.00	0.50	0.86	0.49	1.00	0.50	1.00	0.50	0.40	0.32	-0.03	0.90	0.90	**0.95**	0.91
CiC4876-07	1	0.47	0.41	0.14	0.00	0.00	0.00	0.00	0.25	0.22	0.50	0.38	1.00	0.50	0.86	0.49	1.00	0.50	1.00	0.50	0.20	0.18	-0.08	0.84	0.85	**0.93**	0.86
CiC4884-01	1	0.11	0.11	-0.05	0.10	0.10	0.00	0.00	0.17	0.15	0.25	0.22	0.00	0.00	0.00	0.00	0.00	0.00	0.00	0.00	0.25	0.22	-0.02	-0.05	-0.03	0.03	-0.04
CiC4893-01	1	0.47	0.35	0.26	0.00	0.00	0.00	0.00	0.08	0.08	0.25	0.22	1.00	0.50	1.00	0.50	1.00	0.50	1.00	0.50	0.20	0.18	0.02	0.95	0.95	**0.96**	0.95
CiC4894-09	1	0.14	0.15	-0.07	0.00	0.00	0.00	0.00	0.42	0.33	0.00	0.00	0.00	0.00	0.14	0.13	0.00	0.00	0.00	0.00	0.20	0.18	-0.21	-0.03	0.15	0.27	0.16
CiC4905-01	1	0.29	0.31	-0.08	0.00	0.00	0.00	0.00	0.58	0.47	0.25	0.22	1.00	0.50	0.57	0.41	0.00	0.00	0.00	0.00	0.00	0.00	-0.19	0.18	0.31	**0.39**	0.33
CiC4932-01	1	0.25	0.22	0.13	0.00	0.00	0.00	0.00	0.33	0.44	0.50	0.38	1.00	0.50	0.43	0.34	0.00	0.00	0.00	0.00	0.00	0.00	0.30	0.48	0.25	**0.34**	0.27
CiC4950-06	1	0.15	0.17	-0.08	0.10	0.10	0.00	0.00	0.08	0.08	0.00	0.00	0.00	0.00	0.00	0.00	1.00	0.50	1.00	0.50	0.00	0.00	0.01	-0.04	-0.05	0.00	-0.05
CiC4975-01	1	0.50	0.30	0.40	0.10	0.10	0.80	0.48	0.08	0.08	0.00	0.38	1.00	0.50	0.00	0.49	1.00	0.50	1.00	0.50	0.25	0.22	-0.34	0.68	0.76	**0.91**	0.90
CiC4975-03	1	0.46	0.31	0.33	0.00	0.00	0.00	0.00	0.08	0.08	0.25	0.22	1.00	0.50	0.43	0.34	1.00	0.50	1.00	0.50	0.20	0.18	0.02	0.95	0.95	**0.95**	0.95
CiC4993-03	1	0.35	0.26	0.26	0.00	0.00	0.40	0.32	0.42	0.47	0.00	0.38	0.00	0.00	0.00	0.24	1.00	0.50	1.00	0.50	0.00	0.38	0.09	0.27	0.20	**0.41**	0.32
CiC5001-05	1	0.30	0.36	-0.21	0.00	0.00	1.00	0.50	0.58	0.41	0.50	0.38	0.00	0.00	0.14	0.13	0.00	0.00	1.00	0.50	0.25	0.22	-0.46	-0.08	0.26	**0.44**	0.25
CiC5003-01	1	0.38	0.39	-0.03	0.00	0.00	0.00	0.00	0.67	0.49	0.50	0.38	1.00	0.50	0.86	0.49	0.00	0.00	0.00	0.00	0.20	0.18	-0.32	0.39	0.53	**0.69**	0.55
CiC5037-01	1	0.45	0.28	0.39	0.00	0.00	0.00	0.00	0.17	0.15	0.00	0.00	1.00	0.50	0.14	0.13	1.00	0.50	1.00	0.50	0.40	0.48	-0.03	0.90	0.90	**0.95**	0.91
CiC5072-01	1	0.28	0.22	0.21	0.00	0.00	0.80	0.48	0.42	0.33	0.25	0.47	0.00	0.00	0.00	0.24	0.00	0.00	0.00	0.00	0.20	0.18	-0.37	0.11	0.35	**0.32**	0.16
CiC5072-02	1	0.45	0.43	0.06	0.00	0.00	0.20	0.18	0.83	0.49	0.50	0.38	1.00	0.50	0.00	0.49	1.00	0.50	1.00	0.50	0.20	0.18	-0.60	0.17	0.48	**0.79**	0.56
CiC5076-06	1	0.14	0.15	-0.07	0.00	0.00	0.00	0.00	0.00	0.00	0.25	0.22	0.00	0.00	0.00	0.00	1.00	0.50	1.00	0.50	0.00	0.00	M	M		**0.00**	
CiC5085-01	1	0.44	0.43	0.04	0.10	0.10	0.60	0.42	0.50	0.44	0.00	0.00	1.00	0.50	0.43	0.46	1.00	0.50	1.00	0.50	0.00	0.00	-0.14	0.33	0.41	**0.54**	0.55
CiC5087-01	1	0.15	0.17	-0.08	0.00	0.00	0.00	0.00	0.17	0.15	0.00	0.00	0.00	0.00	0.00	0.00	1.00	0.50	1.00	0.50	0.00	0.00	-0.03	-0.01	0.02	0.09	0.03
CiC5087-03	1	0.09	0.09	-0.04	0.00	0.00	0.00	0.00	0.25	0.22	0.25	0.22	0.00	0.00	0.00	0.00	0.00	0.00	0.00	0.00	0.00	0.00	-0.08	-0.02	0.06	0.16	0.08
CiC5089-02	1	0.29	0.31	-0.08	0.10	0.10	0.00	0.00	0.33	0.38	0.25	0.22	0.00	0.00	0.57	0.41	1.00	0.50	1.00	0.50	0.00	0.00	0.14	0.22	0.10	0.10	0.09
CiC5089-06	1	0.29	0.35	-0.21	0.00	0.00	0.00	0.00	0.50	0.38	0.25	0.22	1.00	0.50	0.43	0.34	0.00	0.00	1.00	0.50	0.20	0.18	-0.28	-0.03	0.19	**0.37**	0.21
CiC5118-01	1	0.46	0.26	0.44	0.00	0.00	0.00	0.00	0.33	0.28	0.25	0.22	1.00	0.50	0.14	0.13	0.00	0.00	1.00	0.50	0.00	0.00	-0.14	0.79	0.82	**0.88**	0.81
CiC5118-03	1	0.45	0.39	0.15	0.00	0.00	0.00	0.00	0.33	0.28	0.50	0.38	1.00	0.50	0.86	0.49	0.00	0.00	1.00	0.50	0.20	0.18	-0.14	0.78	0.81	**0.88**	0.81
CiC5132-01	1	0.42	0.30	0.30	0.10	0.10	0.00	0.00	0.33	0.28	0.25	0.22	1.00	0.50	0.86	0.49	0.00	0.00	0.00	0.00	0.20	0.18	-0.11	0.72	0.75	**0.82**	0.75
CiC5132-02	1	0.41	0.28	0.33	0.00	0.00	0.00	0.00	0.33	0.28	0.25	0.22	1.00	0.50	0.86	0.49	0.00	0.00	0.00	0.00	0.20	0.18	-0.14	0.78	0.81	**0.88**	0.81
CiC5164-02	1	0.43	0.37	0.15	0.30	0.26	0.00	0.00	0.33	0.28	0.25	0.22	1.00	0.50	0.14	0.13	1.00	0.50	1.00	0.50	0.00	0.00	-0.13	0.59	0.63	**0.71**	0.62
CiC5171-05	1	0.48	0.31	0.35	0.00	0.00	0.00	0.00	0.17	0.15	0.50	0.38	1.00	0.50	0.71	0.46	0.00	0.00	1.00	0.50	0.20	0.42	-0.03	0.90	0.90	**0.93**	0.91
CiC5171-06	1	0.48	0.37	0.24	0.10	0.10	0.00	0.00	0.08	0.08	0.50	0.38	1.00	0.50	1.00	0.50	0.00	0.00	1.00	0.50	0.40	0.48	0.01	0.90	0.90	**0.92**	0.90
CiC5181-03	1	0.30	0.22	0.27	0.00	0.00	0.00	0.00	0.42	0.50	0.00	0.00	0.00	0.00	0.00	0.00	1.00	0.50	1.00	0.50	0.00	0.00	0.22	0.59	0.47	**0.54**	0.49
CiC5209-05	1	0.42	0.37	0.12	0.00	0.00	0.20	0.18	0.50	0.38	0.50	0.38	1.00	0.50	1.00	0.50	0.00	0.00	0.00	0.00	0.20	0.18	-0.24	0.57	0.66	**0.83**	0.72
CiC5256-01	1	0.46	0.28	0.41	0.00	0.00	0.00	0.00	0.25	0.33	0.00	0.00	1.00	0.50	0.71	0.46	1.00	0.50	0.00	0.00	0.20	0.18	0.30	0.83	0.75	**0.72**	0.76
CiC5261-01	1	0.44	0.38	0.14	0.30	0.38	0.00	0.00	0.00	0.00	0.75	0.47	0.00	0.00	1.00	0.50	0.00	0.00	1.00	0.50	0.00	0.32	0.26	0.79	0.72	0.21	0.21
CiC5266-02	1	0.23	0.22	0.04	0.00	0.00	1.00	0.50	0.33	0.28	0.00	0.00	0.00	0.00	0.00	0.24	0.00	0.00	1.00	0.50	0.25	0.22	-0.24	-0.01	0.18	**0.29**	0.12
CiC5327-03	1	0.46	0.28	0.41	0.00	0.00	0.00	0.00	0.00	0.00	0.25	0.22	1.00	0.50	0.57	0.41	1.00	0.50	1.00	0.50	0.20	0.18	1.00	1.00	1.00	**0.97**	1.00
CiC5327-04	1	0.46	0.28	0.41	0.00	0.00	0.00	0.00	0.00	0.00	0.25	0.22	1.00	0.50	0.57	0.41	1.00	0.50	1.00	0.50	0.20	0.18	1.00	1.00	1.00	**0.97**	1.00
CiC5332-01	1	0.17	0.19	-0.09	0.00	0.00	0.00	0.00	0.25	0.22	0.00	0.00	0.00	0.00	0.00	0.00	1.00	0.50	1.00	0.50	0.00	0.00	-0.08	-0.02	0.06	0.10	0.08
CiC5349-01	1	0.23	0.26	-0.14	0.40	0.32	0.00	0.00	0.25	0.22	0.25	0.22	0.00	0.00	0.00	0.00	0.50	0.38	1.00	0.50	0.00	0.00	-0.14	-0.12	0.02	0.04	-0.02
CiC5355-05	1	0.14	0.11	0.20	0.00	0.00	0.60	0.42	0.08	0.22	0.00	0.00	0.00	0.00	0.00	0.00	0.00	0.00	0.00	0.00	0.20	0.18	0.21	0.29	0.11	0.13	0.04
CiC5363-01	1	0.49	0.46	0.07	0.00	0.00	0.80	0.48	0.50	0.44	0.50	0.38	1.00	0.50	0.14	0.50	1.00	0.50	1.00	0.50	0.60	0.42	-0.24	0.38	0.50	**0.64**	0.63
CiC5365-01	1	0.48	0.33	0.31	0.00	0.00	0.00	0.00	0.25	0.22	0.50	0.38	1.00	0.50	0.57	0.41	0.00	0.00	1.00	0.50	0.00	0.48	-0.08	0.84	0.85	**0.83**	0.86
CiC5376-05	1	0.46	0.30	0.36	0.10	0.10	0.00	0.00	0.08	0.08	0.25	0.22	1.00	0.50	0.71	0.46	0.00	0.00	1.00	0.50	0.20	0.18	0.01	0.90	0.90	**0.91**	0.90
CiC5391-01	1	0.10	0.11	-0.05	0.00	0.00	0.00	0.00	0.42	0.33	0.00	0.00	0.00	0.00	0.00	0.00	0.00	0.00	0.00	0.00	0.00	0.00	-0.21	-0.03	0.15	0.27	0.16
CiC5414-02	1	0.39	0.28	0.30	0.30	0.26	0.00	0.00	0.33	0.28	0.00	0.00	0.00	0.00	0.14	0.13	1.00	0.50	1.00	0.50	0.00	0.00	-0.13	0.59	0.63	**0.71**	0.62
CiC5414-03	1	0.39	0.28	0.30	0.30	0.26	0.00	0.00	0.33	0.28	0.00	0.00	0.00	0.00	0.14	0.13	1.00	0.50	1.00	0.50	0.00	0.00	-0.13	0.59	0.63	**0.71**	0.62
CiC5444-05	1	0.29	0.28	0.05	0.00	0.00	0.00	0.00	0.67	0.50	0.00	0.00	0.00	0.00	0.14	0.13	0.00	0.00	1.00	0.50	0.20	0.18	-0.28	0.29	0.45	**0.55**	0.46
CiC5459-04	1	0.20	0.22	-0.12	0.00	0.00	0.00	0.00	0.42	0.33	0.00	0.00	0.00	0.00	0.00	0.00	1.00	0.50	1.00	0.50	0.00	0.00	-0.21	-0.03	0.15	0.22	0.16

CiC5465-01	1	0.06	0.06	-0.02	0.00	0.00	0.00	0.00	0.17	0.15	0.00	0.00	0.00	0.00	0.00	0.00	0.00	0.00	0.00	0.00	0.00	0.00	-0.03	-0.01	0.02	0.13	0.03
CiC5465-04	1	0.39	0.35	0.13	0.10	0.10	0.40	0.32	0.45	0.43	0.00	0.00	1.00	0.50	1.00	0.50	0.00	0.00	0.00	0.00	0.20	0.18	-0.03	0.44	0.46	**0.57**	0.57
CiC5471-01	1	0.25	0.21	0.15	0.00	0.00	0.00	0.38	0.33	0.38	0.25	0.22	0.00	0.00	0.43	0.34	0.00	0.00	0.00	0.00	0.00	0.00	0.39	0.44	0.08	**0.20**	0.18
CiC5481-03	1	0.48	0.35	0.27	0.00	0.00	0.00	0.00	0.08	0.08	0.50	0.38	1.00	0.50	0.57	0.41	1.00	0.50	1.00	0.50	0.20	0.42	0.02	0.95	0.95	**0.95**	0.95
CiC5481-06	1	0.12	0.13	-0.06	0.00	0.00	0.00	0.00	0.08	0.08	0.00	0.00	0.00	0.00	0.00	0.00	0.00	0.00	1.00	0.50	0.20	0.18	0.02	-0.01	-0.02	0.02	-0.01
CiC5485-09	1	0.48	0.45	0.07	0.00	0.00	0.00	0.00	0.22	0.20	1.00	0.50	1.00	0.50	1.00	0.50	0.00	0.00	1.00	0.50	0.40	0.48	-0.06	0.87	0.88	**0.82**	0.89
CiC5492-10	1	0.38	0.28	0.27	0.00	0.00	0.00	0.00	0.75	0.50	0.00	0.00	0.00	0.00	0.14	0.13	0.00	0.00	1.00	0.50	0.00	0.00	-0.47	0.26	0.49	**0.65**	0.51
CiC5507-01	1	0.35	0.42	-0.18	0.70	0.46	0.00	0.00	0.17	0.15	0.50	0.38	0.00	0.00	0.29	0.24	0.00	0.00	1.00	0.50	0.80	0.48	-0.36	-0.11	0.19	**0.29**	0.17
CiC5534-02	1	0.38	0.24	0.37	0.00	0.00	0.00	0.00	0.50	0.44	0.00	0.00	0.00	0.00	0.57	0.41	0.00	0.00	0.00	0.00	0.20	0.18	-0.07	0.59	0.62	**0.71**	0.63
CiC5567-01	1	0.33	0.30	0.09	0.70	0.46	0.00	0.00	0.09	0.43	0.00	0.00	0.00	0.00	0.14	0.13	1.00	0.50	1.00	0.50	0.00	0.00	0.20	0.25	0.07	0.01	-0.06
CiC5577-01	1	0.29	0.28	0.05	0.10	0.10	0.50	0.38	0.58	0.41	0.50	0.38	0.00	0.00	0.14	0.13	0.00	0.00	0.00	0.00	0.20	0.50	-0.29	-0.18	0.09	**0.32**	0.15
CiC5585-03	1	0.45	0.39	0.15	0.00	0.00	0.00	0.00	0.33	0.28	0.50	0.38	1.00	0.50	1.00	0.50	0.00	0.00	1.00	0.50	0.20	0.18	-0.14	0.78	0.81	**0.87**	0.81
CiC5585-04	1	0.18	0.17	0.10	0.00	0.18	0.00	0.00	0.08	0.08	0.00	0.00	0.00	0.00	0.00	0.00	1.00	0.50	1.00	0.50	0.20	0.18	0.67	0.65	-0.06	0.02	-0.05
CiC5589-01	1	0.47	0.30	0.37	0.00	0.00	0.00	0.00	0.00	0.00	0.25	0.22	1.00	0.50	0.57	0.41	1.00	0.50	1.00	0.50	0.40	0.32	1.00	1.00	1.00	**0.97**	1.00
CiC5609-01	1	0.21	0.20	0.05	0.00	0.00	1.00	0.50	0.42	0.33	0.00	0.00	0.00	0.00	0.00	0.00	0.00	0.00	0.00	0.00	0.00	0.32	-0.51	-0.10	0.27	0.29	0.16
CiC5682-01	1	0.30	0.26	0.15	0.10	0.10	0.00	0.00	0.25	0.47	0.00	0.00	1.00	0.50	0.14	0.13	0.00	0.00	1.00	0.50	0.00	0.00	0.44	0.56	0.21	0.20	0.21
CiC5690-01	1	0.37	0.19	0.50	0.00	0.00	0.00	0.00	0.25	0.33	0.25	0.22	0.00	0.00	0.57	0.41	0.00	0.00	0.00	0.00	0.20	0.18	0.30	0.83	0.75	**0.83**	0.76
CiC5692-01	1	0.41	0.46	-0.12	0.00	0.00	0.80	0.48	0.58	0.41	0.25	0.22	1.00	0.50	0.14	0.50	1.00	0.50	1.00	0.50	0.40	0.32	-0.45	-0.15	0.21	**0.37**	0.25
CiC5696-01	1	0.38	0.30	0.24	0.00	0.00	0.00	0.00	0.42	0.41	0.00	0.00	1.00	0.50	1.00	0.50	0.00	0.00	0.00	0.00	0.20	0.18	0.05	0.68	0.66	**0.72**	0.67
CiC5696-02	1	0.47	0.25	0.48	0.00	0.00	1.00	0.50	0.42	0.41	0.00	0.00	1.00	0.50	0.00	0.00	0.00	0.00	0.00	0.00	0.20	0.18	-0.24	0.44	0.54	**0.72**	0.67
CiC5702-01	1	0.10	0.11	-0.05	0.00	0.00	0.00	0.00	0.42	0.33	0.00	0.00	0.00	0.00	0.00	0.00	0.00	0.00	0.00	0.00	0.00	0.00	-0.21	-0.03	0.15	0.29	0.16
CiC5704-01	1	0.50	0.14	0.73	0.00	0.00	0.60	0.42	0.08	0.33	0.00	0.38	0.00	0.00	0.33	0.44	0.00	0.00	0.00	0.00	0.00	0.32	0.39	0.77	0.62	**0.71**	0.76
CiC5708-09	1	0.47	0.26	0.45	0.00	0.00	0.00	0.00	0.08	0.08	0.25	0.22	0.00	0.00	0.57	0.41	1.00	0.50	1.00	0.50	0.20	0.18	0.02	0.95	0.95	**0.95**	0.95
CiC5718-01	1	0.27	0.20	0.27	0.00	0.00	1.00	0.50	0.33	0.28	0.25	0.22	1.00	0.50	0.14	0.50	0.00	0.00	0.00	0.00	0.00	0.00	-0.24	-0.01	0.18	0.19	0.12
CiC5719-01	1	0.24	0.20	0.16	0.20	0.42	0.00	0.00	0.08	0.08	0.00	0.00	0.00	0.00	0.00	0.00	1.00	0.50	1.00	0.50	0.20	0.18	0.47	0.55	0.15	0.17	0.17
CiC5719-08	1	0.28	0.26	0.08	0.00	0.00	0.00	0.00	0.58	0.47	0.25	0.22	1.00	0.50	0.43	0.46	0.00	0.00	0.00	0.00	0.00	0.00	-0.19	0.18	0.31	**0.40**	0.33
CiC5720-01	1	0.24	0.28	-0.15	0.00	0.00	0.00	0.00	0.58	0.41	0.00	0.00	1.00	0.50	0.43	0.34	0.00	0.00	0.00	0.00	0.00	0.00	-0.36	-0.04	0.24	**0.44**	0.25
CiC5725-02	1	0.42	0.22	0.47	0.00	0.00	0.00	0.00	0.08	0.08	0.00	0.00	1.00	0.50	0.14	0.13	1.00	0.50	1.00	0.50	0.00	0.00	0.02	0.95	0.95	**0.96**	0.95
CiC5725-08	1	0.48	0.37	0.23	0.00	0.00	0.00	0.00	0.08	0.08	0.50	0.38	1.00	0.50	0.86	0.49	1.00	0.50	1.00	0.50	0.20	0.18	0.02	0.95	0.95	**0.96**	0.95
CiC5737-03	1	0.48	0.43	0.12	0.00	0.00	1.00	0.50	0.64	0.43	0.00	0.38	1.00	0.50	0.17	0.15	1.00	0.50	1.00	0.50	0.00	0.00	-0.61	0.21	0.51	**0.81**	0.66
CiC5748-02	1	0.12	0.13	-0.06	0.10	0.10	0.00	0.00	0.42	0.33	0.00	0.00	0.00	0.00	0.00	0.00	0.00	0.00	0.00	0.00	0.00	0.00	-0.17	-0.08	0.08	0.16	0.07
CiC5757-01	1	0.17	0.19	-0.09	0.50	0.38	0.00	0.00	0.00	0.00	0.00	0.00	0.00	0.00	0.00	0.00	0.00	0.00	1.00	0.50	0.00	0.00	-0.28	0.00	0.22	**0.36**	0.24
CiC5774-01	1	0.48	0.37	0.23	0.00	0.00	0.00	0.00	0.17	0.15	0.25	0.22	1.00	0.50	1.00	0.50	1.00	0.50	1.00	0.50	0.20	0.42	-0.03	0.90	0.90	**0.95**	0.91
CiC5781-04	1	0.30	0.37	-0.22	0.10	0.10	0.00	0.00	0.33	0.28	0.25	0.22	1.00	0.50	0.14	0.13	1.00	0.50	1.00	0.50	0.50	0.38	-0.11	-0.07	0.04	0.05	0.03
CiC5781-07	1	0.48	0.43	0.12	0.10	0.10	0.00	0.00	0.33	0.28	0.50	0.38	1.00	0.50	0.86	0.49	1.00	0.50	1.00	0.50	0.00	0.00	-0.11	0.66	0.69	0.05	0.03
CiC5785-01	1	0.07	0.07	-0.03	0.10	0.10	0.00	0.00	0.17	0.15	0.00	0.00	0.00	0.00	0.00	0.00	0.00	0.00	0.00	0.00	0.00	0.00	-0.02	-0.05	-0.03	0.03	-0.04
CiC5788-03	1	0.43	0.41	0.07	0.00	0.00	0.00	0.00	0.50	0.38	0.25	0.22	1.00	0.50	0.29	0.24	1.00	0.50	1.00	0.50	0.80	0.48	-0.28	0.63	0.71	**0.84**	0.72
CiC5788-16	1	0.49	0.30	0.41	0.00	0.00	1.00	0.50	0.08	0.08	0.25	0.47	1.00	0.50	0.00	0.49	1.00	0.50	1.00	0.50	0.00	0.00	-0.71	0.69	0.82	**0.95**	0.95
CiC5796-03	1	0.49	0.41	0.18	0.00	0.00	0.00	0.00	0.17	0.15	0.75	0.47	1.00	0.50	1.00	0.50	1.00	0.50	1.00	0.50	0.20	0.50	-0.03	0.90	0.90	**0.93**	0.91
CiC5796-12	1	0.37	0.23	0.40	0.30	0.26	0.00	0.00	0.08	0.08	0.25	0.22	0.00	0.00	0.00	0.00	1.00	0.50	1.00	0.50	0.00	0.00	-0.08	0.76	0.78	**0.83**	0.80
CiC5814-01	1	0.46	0.30	0.35	0.00	0.00	0.00	0.00	0.50	0.38	0.00	0.00	1.00	0.50	0.00	0.00	1.00	0.50	1.00	0.50	0.00	0.32	-0.28	0.68	0.75	**0.77**	0.72
CiC5815-02	1	0.23	0.26	-0.14	0.60	0.42	0.20	0.18	0.00	0.00	0.00	0.00	0.00	0.00	0.00	0.00	1.00	0.50	1.00	0.50	0.00	0.00	-0.32	-0.05	0.20	**0.44**	0.30
CiC5825-01	1	0.23	0.15	0.35	0.00	0.00	0.00	0.00	0.50	0.44	0.25	0.22	0.00	0.00	0.00	0.41	0.00	0.00	0.00	0.00	0.00	0.00	-0.07	0.22	0.27	**0.35**	0.28
CiC5825-03	1	0.30	0.29	0.04	0.22	0.20	0.75	0.47	0.25	0.22	0.00	0.38	1.00	0.50	0.00	0.00	0.00	0.00	1.00	0.50	0.00	0.00	-0.21	-0.15	0.05	0.01	-0.05
CiC5842-02	1	0.32	0.22	0.32	0.00	0.00	0.00	0.00	0.25	0.50	0.25	0.22	1.00	0.50	0.71	0.46	0.00	0.00	0.00	0.00	0.00	0.00	0.54	0.75	0.46	**0.48**	0.48
CiC5851-01	1	0.12	0.13	-0.06	0.00	0.00	0.00	0.00	0.42	0.33	0.25	0.22	0.00	0.00	0.00	0.00	0.00	0.00	0.00	0.00	0.00	0.00	-0.21	-0.03	0.15	0.29	0.16
CiC5860-01	1	0.44	0.37	0.18	0.00	0.00	0.00	0.00	0.33	0.28	0.50	0.38	1.00	0.50	0.86	0.49	0.00	0.00	1.00	0.50	0.20	0.18	-0.14	0.78	0.81	**0.88**	0.81

CiC5860-02	1	0.44	0.50	-0.13	0.80	0.48	0.00	0.00	0.33	0.28	0.75	0.47	1.00	0.50	0.14	0.13	0.00	0.00	1.00	0.50	0.40	0.48	-0.43	-0.20	0.16	**0.26**	0.11
CiC5911-01	1	0.05	0.06	-0.02	0.00	0.00	0.00	0.00	0.17	0.15	0.00	0.00	0.00	0.00	0.00	0.00	0.00	0.00	0.00	0.00	0.00	0.00	-0.03	-0.01	0.02	0.10	0.03
CiC5938-01	1	0.43	0.26	0.41	0.10	0.10	0.00	0.00	0.17	0.15	0.00	0.00	1.00	0.50	0.14	0.13	1.00	0.50	1.00	0.50	0.00	0.32	-0.02	0.84	0.84	**0.89**	0.85
CiC5938-02	1	0.39	0.24	0.40	0.10	0.10	0.00	0.00	0.25	0.41	0.25	0.22	0.00	0.00	0.14	0.13	1.00	0.50	1.00	0.50	0.00	0.32	0.37	0.74	0.59	**0.63**	0.59
CiC5948-03	1	0.24	0.20	0.16	0.00	0.00	0.00	0.00	0.33	0.28	0.00	0.00	1.00	0.50	0.14	0.34	0.00	0.00	0.00	0.00	0.40	0.48	-0.14	-0.02	0.11	**0.28**	0.12
CiC5950-02	1	0.48	0.37	0.24	0.00	0.00	0.00	0.00	0.00	0.00	0.25	0.22	1.00	0.50	1.00	0.50	1.00	0.50	1.00	0.50	0.20	0.18	1.00	1.00	1.00	**0.97**	1.00
CiC5950-03	1	0.12	0.09	0.25	0.00	0.18	0.00	0.00	0.00	0.00	0.00	0.00	0.00	0.00	0.00	0.00	0.00	0.00	1.00	0.50	0.00	0.00	1.00	1.00	-0.02	0.05	0.02
CiC5979-03	1	0.38	0.24	0.37	0.10	0.50	0.00	0.00	0.67	0.50	0.25	0.22	0.00	0.00	0.00	0.00	1.00	0.50	0.00	0.00	0.00	0.00	0.23	0.38	0.19	0.00	-0.05
CiC6022-01	1	0.18	0.17	0.10	0.00	0.00	0.00	0.00	0.42	0.41	0.25	0.22	0.00	0.00	0.29	0.24	0.00	0.00	0.00	0.00	0.00	0.00	0.05	0.26	0.22	**0.34**	0.24
CiC6110-02	1	0.36	0.36	0.02	0.00	0.00	0.75	0.47	0.67	0.50	0.00	0.00	0.00	0.00	0.14	0.13	1.00	0.50	1.00	0.50	0.00	0.32	-0.34	0.11	0.34	**0.57**	0.46
CiC6116-02	1	0.49	0.21	0.58	0.20	0.18	0.00	0.00	0.30	0.26	0.25	0.47	0.00	0.00	0.29	0.24	0.00	0.00	0.00	0.00	0.00	0.00	-0.09	0.70	0.72	**0.66**	0.71
CiC6116-04	1	0.45	0.31	0.31	0.20	0.18	0.00	0.00	0.17	0.15	0.50	0.38	1.00	0.50	0.29	0.24	0.00	0.00	1.00	0.50	0.00	0.00	-0.04	0.78	0.78	**0.86**	0.79
CiC6122-02	1	0.42	0.24	0.44	0.10	0.10	0.00	0.00	0.42	0.41	0.00	0.00	1.00	0.50	0.43	0.34	0.00	0.00	0.00	0.00	0.20	0.18	0.04	0.61	0.60	**0.66**	0.60
CiC6122-04	1	0.44	0.41	0.09	0.00	0.00	0.00	0.00	0.42	0.33	0.75	0.47	0.00	0.00	1.00	0.50	0.00	0.00	1.00	0.50	0.00	0.32	-0.21	0.66	0.72	0.21	0.16
CiC6128-06	1	0.14	0.15	-0.07	0.30	0.26	0.00	0.00	0.00	0.00	0.00	0.00	0.00	0.00	0.00	0.00	0.00	0.00	1.00	0.50	0.00	0.00	-0.12	0.00	0.10	0.19	0.13
CiC6172-03	1	0.39	0.42	-0.06	0.00	0.20	0.00	0.00	0.83	0.50	0.00	0.00	1.00	0.50	0.29	0.24	0.00	0.00	1.00	0.50	0.20	0.42	-0.23	0.14	0.30	**0.33**	0.26
CiC6193-01	1	0.50	0.22	0.56	0.00	0.00	0.20	0.18	0.00	0.00	0.00	0.50	1.00	0.50	0.00	0.24	1.00	0.50	1.00	0.50	0.33	0.50	-0.05	0.95	0.95	**0.97**	1.00
CiC6193-09	1	0.48	0.38	0.21	0.00	0.00	0.00	0.00	0.08	0.08	0.25	0.22	1.00	0.50	1.00	0.50	1.00	0.50	1.00	0.50	0.20	0.18	0.02	0.95	0.95	**0.87**	0.95
CiC6213-02	1	0.43	0.26	0.41	0.00	0.00	0.00	0.00	0.08	0.08	0.25	0.22	1.00	0.50	0.14	0.13	1.00	0.50	1.00	0.50	0.20	0.18	0.02	0.95	0.95	**0.95**	0.95
CiC6213-07	1	0.49	0.39	0.22	0.00	0.00	0.00	0.00	0.17	0.15	0.75	0.47	1.00	0.50	0.86	0.49	1.00	0.50	1.00	0.50	0.00	0.48	-0.03	0.90	0.90	**0.94**	0.91
CiC6235-01	1	0.09	0.09	-0.04	0.00	0.00	0.00	0.00	0.25	0.22	0.25	0.22	0.00	0.00	0.00	0.00	0.00	0.00	0.00	0.00	0.00	0.00	-0.08	-0.02	0.06	0.16	0.08
CiC6243-01	1	0.48	0.25	0.48	0.00	0.00	0.00	0.00	0.08	0.22	0.25	0.47	1.00	0.50	0.29	0.24	0.00	0.00	1.00	0.50	0.00	0.00	0.65	0.95	0.85	**0.84**	0.85
CiC6243-03	1	0.49	0.17	0.65	0.00	0.00	0.00	0.00	0.08	0.22	0.25	0.47	1.00	0.50	0.00	0.41	0.00	0.00	1.00	0.50	0.00	0.00	0.65	0.95	0.85	**0.84**	0.85
CiC6249-01	1	0.32	0.41	-0.25	0.00	0.00	0.00	0.00	0.58	0.41	0.00	0.00	1.00	0.50	0.71	0.46	1.00	0.50	1.00	0.50	0.00	0.00	-0.36	-0.04	0.24	0.34	0.25
CiC6249-02	1	0.36	0.31	0.12	0.00	0.00	0.00	0.00	0.67	0.44	0.25	0.22	0.00	0.00	0.29	0.24	0.00	0.00	1.00	0.50	0.20	0.18	-0.45	0.46	0.63	**0.82**	0.64
CiC6259-01	1	0.38	0.22	0.43	0.20	0.18	0.00	0.00	0.00	0.00	0.25	0.22	1.00	0.50	0.00	0.00	1.00	0.50	1.00	0.50	0.00	0.00	-0.05	0.89	0.89	**0.89**	0.90
CiC6259-03	1	0.05	0.06	-0.02	0.00	0.00	0.00	0.00	0.17	0.15	0.00	0.00	0.00	0.00	0.00	0.00	0.00	0.00	0.00	0.00	0.00	0.00	-0.03	-0.01	0.02	0.10	0.03
CiC6260-02	1	0.32	0.41	-0.25	0.90	0.50	0.00	0.00	0.00	0.00	0.25	0.22	1.00	0.50	0.43	0.34	0.00	0.00	1.00	0.50	0.00	0.00	-0.80	0.00	0.44	**0.82**	0.47
CiC6288-02	1	0.45	0.43	0.06	0.00	0.00	0.00	0.00	0.42	0.33	0.50	0.38	1.00	0.50	0.71	0.46	1.00	0.50	1.00	0.50	0.20	0.18	-0.21	0.71	0.76	**0.81**	0.77
CiC6288-04	1	0.07	0.07	-0.03	0.00	0.00	0.00	0.00	0.25	0.22	0.00	0.00	0.00	0.00	0.00	0.00	0.00	0.00	0.00	0.00	0.00	0.00	-0.08	-0.02	0.06	0.17	0.08
CiC6294-02	1	0.25	0.26	-0.02	0.00	0.00	0.00	0.00	0.50	0.44	0.00	0.00	0.00	0.00	0.29	0.24	0.00	0.00	1.00	0.50	0.20	0.18	-0.07	0.22	0.27	**0.38**	0.28
CiC6306-01	1	0.24	0.28	-0.15	0.00	0.00	0.00	0.00	0.50	0.38	0.25	0.22	1.00	0.50	0.57	0.41	0.00	0.00	0.00	0.00	0.00	0.00	-0.28	-0.03	0.19	**0.32**	0.21
CiC6306-06	1	0.39	0.33	0.17	0.00	0.00	0.00	0.00	0.45	0.43	0.25	0.22	0.00	0.00	0.29	0.24	1.00	0.50	1.00	0.50	0.20	0.18	0.03	0.62	0.61	**0.55**	0.61
CiC6314-14	1	0.30	0.30	0.03	0.00	0.00	0.00	0.00	0.58	0.50	0.25	0.22	1.00	0.50	0.57	0.41	0.00	0.00	0.00	0.00	0.20	0.18	-0.12	0.33	0.40	**0.52**	0.41
CiC6315-01	1	0.26	0.23	0.12	0.00	0.00	0.00	0.00	0.58	0.50	0.00	0.00	0.00	0.00	0.33	0.28	0.00	0.00	0.00	0.00	0.20	0.18	-0.11	0.32	0.39	**0.59**	0.41
CiC6320-02	1	0.45	0.43	0.06	0.00	0.00	0.00	0.00	0.42	0.33	0.25	0.22	1.00	0.50	0.71	0.46	1.00	0.50	1.00	0.50	0.40	0.32	-0.21	0.71	0.76	**0.81**	0.77
CiC6320-04	1	0.45	0.45	0.02	0.00	0.00	0.50	0.38	0.40	0.32	0.25	0.22	1.00	0.50	0.71	0.46	1.00	0.50	1.00	0.50	0.20	0.18	-0.20	0.67	0.72	**0.74**	0.79
CiC6324-01	1	0.43	0.44	-0.02	0.00	0.00	0.00	0.00	0.50	0.44	0.25	0.22	1.00	0.50	0.86	0.49	1.00	0.50	1.00	0.50	0.20	0.18	-0.07	0.59	0.62	**0.72**	0.63
CiC6324-03	1	0.49	0.25	0.49	0.22	0.35	0.00	0.00	0.33	0.38	0.00	0.00	0.00	0.00	0.29	0.24	0.00	0.00	1.00	0.50	0.00	0.32	0.27	0.62	0.47	**0.41**	0.40
CiC6391-02	1	0.49	0.31	0.36	0.00	0.00	0.00	0.00	0.17	0.15	0.75	0.47	1.00	0.50	0.71	0.46	0.00	0.00	1.00	0.50	0.00	0.32	-0.03	0.90	0.90	**0.94**	0.91
CiC6391-03	1	0.44	0.31	0.29	0.00	0.00	0.00	0.00	0.25	0.22	0.25	0.22	1.00	0.50	0.71	0.46	0.00	0.00	1.00	0.50	0.20	0.18	-0.08	0.84	0.85	**0.90**	0.86
CiC6441-17	1	0.20	0.22	-0.12	0.00	0.00	0.00	0.00	0.33	0.28	0.00	0.00	0.00	0.00	0.14	0.13	1.00	0.50	1.00	0.50	0.00	0.00	-0.14	-0.02	0.11	0.13	0.12
CiC6458-12	1	0.46	0.39	0.17	0.00	0.00	0.00	0.00	0.25	0.22	0.50	0.38	1.00	0.50	0.57	0.41	1.00	0.50	1.00	0.50	0.20	0.18	-0.08	0.84	0.85	**0.82**	0.86
AocM503	1	0.46	0.41	0.12	0.00	0.00	0.00	0.00	0.33	0.28	0.50	0.38	1.00	0.50	0.71	0.46	1.00	0.50	1.00	0.50	0.20	0.18	-0.14	0.78	0.81	**0.82**	0.81
AocP256	1	0.50	0.55	-0.09	0.20	0.18	0.80	0.48	0.42	0.33	0.50	0.38	1.00	0.50	0.50	0.49	1.00	0.50	1.00	0.50	0.67	0.44	-0.30	0.33	0.49	**0.68**	0.63
CHI-M-104	1	0.24	0.28	-0.15	0.00	0.00	0.80	0.48	0.08	0.08	0.25	0.22	0.00	0.00	0.43	0.34	0.00	0.00	1.00	0.50	0.20	0.18	-0.44	0.04	0.34	0.02	-0.01
CHI-M-170	1	0.49	0.38	0.24	0.00	0.00	0.00	0.00	0.33	0.28	0.25	0.47	0.00	0.00	0.86	0.49	1.00	0.50	1.00	0.50	0.20	0.18	-0.14	0.78	0.80	**0.82**	0.81

HKT1c800F141	1	0.27	0.09	0.66	0.00	0.00	0.00	0.00	0.00	0.50	0.00	0.00	0.00	0.00	0.43	0.34	0.00	0.00	0.00	0.00	0.20	0.18	1.00	1.00	0.40	**0.38**	0.43
LCY2-M-376	1	0.38	0.39	-0.03	0.00	0.00	0.00	0.00	0.42	0.50	0.25	0.22	1.00	0.50	0.71	0.46	1.00	0.50	1.00	0.50	0.20	0.18	0.22	0.52	0.38	**0.44**	0.40
LCY2-P-243	1	0.44	0.30	0.34	0.00	0.00	0.00	0.00	0.25	0.22	0.25	0.22	1.00	0.50	0.14	0.13	1.00	0.50	1.00	0.50	0.00	0.00	-0.08	0.85	0.86	**0.88**	0.86
LCYB-M-201	1	0.46	0.37	0.20	0.00	0.00	0.00	0.00	0.33	0.28	0.50	0.38	1.00	0.50	0.86	0.49	0.00	0.00	1.00	0.50	0.20	0.42	-0.14	0.78	0.81	**0.88**	0.81
LCYB-M-480	1	0.46	0.37	0.20	0.00	0.00	0.00	0.00	0.33	0.28	0.50	0.38	1.00	0.50	0.86	0.49	0.00	0.00	1.00	0.50	0.20	0.42	-0.14	0.78	0.81	**0.88**	0.81
PKF-M-186	1	0.46	0.37	0.20	0.00	0.00	0.00	0.00	0.50	0.38	1.00	0.50	1.00	0.50	1.00	0.50	0.00	0.00	0.00	0.00	0.00	0.48	-0.28	0.63	0.71	**0.83**	0.72
PSY-M-289	1	0.43	0.43	0.00	0.00	0.00	0.00	0.00	0.55	0.40	0.25	0.22	1.00	0.50	0.71	0.46	1.00	0.50	1.00	0.50	0.20	0.18	-0.32	0.60	0.70	**0.77**	0.71
TRPA-M-287	1	0.41	0.35	0.15	0.00	0.00	0.00	0.00	0.50	0.38	0.50	0.38	1.00	0.50	1.00	0.50	0.00	0.00	0.00	0.00	0.20	0.18	-0.28	0.63	0.71	**0.83**	0.72
TRPA-M-370	1	0.41	0.35	0.15	0.00	0.00	0.00	0.00	0.50	0.38	0.50	0.38	1.00	0.50	1.00	0.50	0.00	0.00	0.00	0.00	0.20	0.18	-0.28	0.63	0.71	**0.83**	0.72
TScMI331	1	0.29	0.28	0.05	0.00	0.00	0.00	0.00	0.42	0.47	0.25	0.22	1.00	0.50	0.71	0.46	0.00	0.00	0.00	0.00	0.00	0.00	0.17	0.42	0.30	**0.40**	0.32
CiC0913-01	0	0.46	0.33	0.29	0.00	0.00	0.00	0.00	0.33	0.28	0.00	0.00	1.00	0.50	0.67	0.50	1.00	0.50	1.00	0.50	0.25	0.22	-0.14	0.78	0.81	**0.92**	0.81
CiC1317-03	0	0.25	0.22	0.13	0.00	0.00	0.20	0.18	0.42	0.33	0.00	0.38	0.00	0.00	0.00	0.00	1.00	0.50	1.00	0.50	0.00	0.00	-0.18	-0.07	0.09	**0.27**	0.16
CiC1820-01	0	0.46	0.24	0.49	0.00	0.00	0.20	0.18	0.42	0.33	0.25	0.22	0.00	0.00	0.14	0.50	1.00	0.50	0.75	0.47	0.00	0.32	-0.18	0.65	0.70	**0.82**	0.77
CiC2335-06	0	0.43	0.19	0.58	0.00	0.00	0.00	0.00	0.00	0.00	0.75	0.47	1.00	0.50	0.14	0.13	1.00	0.50	0.00	0.00	0.40	0.48	1.00	1.00	1.00	**0.97**	1.00
CiC3770-06	0	0.36	0.25	0.33	0.00	0.00	0.00	0.00	0.00	0.00	1.00	0.50	0.00	0.00	1.00	0.50	0.00	0.00	0.00	0.00	0.00	0.38	1.00	1.00	1.00	**0.00**	
CiC4175-08	0	0.39	0.49	-0.25	0.00	0.00	0.00	0.00	0.75	0.47	0.50	0.38	1.00	0.50	0.83	0.49	1.00	0.50	1.00	0.50	0.40	0.32	-0.56	-0.05	0.33	**0.60**	0.34
CiC4255-03	0	0.48	0.38	0.21	0.00	0.00	0.25	0.22	0.42	0.33	0.25	0.22	1.00	0.50	0.71	0.50	1.00	0.50	1.00	0.50	0.00	0.38	-0.18	0.64	0.69	**0.83**	0.76
CiC4841-04	0	0.46	0.30	0.36	0.20	0.18	0.00	0.00	0.08	0.22	0.25	0.22	0.00	0.00	0.86	0.49	1.00	0.50	1.00	0.50	0.00	0.00	0.37	0.84	0.74	**0.75**	0.73
CiC4973-03	0	0.42	0.07	0.83	0.00	0.00	0.00	0.00	0.00	0.00	0.00	0.00	0.00	0.00	0.57	0.41	0.00	0.00	0.00	0.00	0.00	0.00	1.00	1.00	1.00	**0.00**	
CiC5528-03	0	0.46	0.31	0.35	0.00	0.00	0.00	0.00	0.33	0.28	0.25	0.22	1.00	0.50	0.60	0.50	1.00	0.50	1.00	0.50	0.00	0.32	-0.14	0.78	0.81	**0.89**	0.81
CiC5659-01	0	0.49	0.22	0.56	0.00	0.00	0.00	0.00	0.17	0.15	0.00	0.38	0.00	0.00	0.43	0.46	1.00	0.50	1.00	0.50	0.20	0.42	-0.03	0.90	0.90	**0.95**	0.91
CiC5744-07	0	0.50	0.13	0.74	0.00	0.00	0.00	0.00	0.08	0.08	0.00	0.38	0.00	0.00	0.57	0.49	1.00	0.50	0.00	0.00	0.00	0.32	0.02	0.95	0.95	**0.95**	0.95
CiC5744-08	0	0.42	0.13	0.70	0.00	0.00	0.00	0.00	0.08	0.41	0.00	0.00	0.00	0.00	0.43	0.34	1.00	0.50	0.00	0.00	0.20	0.18	0.82	0.94	0.64	**0.64**	0.66
CiC5839-02	0	0.49	0.37	0.25	0.10	0.10	0.20	0.18	0.08	0.22	0.50	0.38	1.00	0.50	0.86	0.49	1.00	0.50	1.00	0.50	0.20	0.50	0.38	0.84	0.73	**0.79**	0.79
CiC5920-04	0	0.40	0.30	0.27	0.00	0.00	0.00	0.00	0.42	0.47	0.00	0.00	1.00	0.50	0.43	0.46	0.00	0.00	1.00	0.50	0.00	0.00	0.17	0.64	0.57	**0.58**	0.58
CiC6234-01	0	0.44	0.33	0.26	0.00	0.00	0.00	0.00	0.58	0.47	0.00	0.00	1.00	0.50	0.71	0.46	1.00	0.50	0.00	0.00	0.00	0.00	-0.19	0.50	0.58	**0.62**	0.59
CiC6363-05	0	0.49	0.33	0.33	0.00	0.00	0.80	0.48	0.25	0.33	0.25	0.47	1.00	0.50	0.00	0.24	1.00	0.50	1.00	0.50	0.00	0.32	-0.04	0.60	0.62	**0.82**	0.76
CiC6401-02	0	0.49	0.37	0.25	0.00	0.00	0.00	0.00	0.42	0.33	0.50	0.38	1.00	0.50	1.00	0.50	1.00	0.50	0.00	0.00	0.20	0.42	-0.21	0.71	0.76	**0.82**	0.77
AocC634	0	0.33	0.23	0.32	0.00	0.00	0.00	0.00	0.00	0.00	0.75	0.47	0.00	0.00	1.00	0.50	0.00	0.00	0.00	0.00	0.00	0.00	1.00	1.00	1.00	0.00	
Cax1C176	0	0.36	0.24	0.33	0.00	0.00	0.00	0.00	0.00	0.00	1.00	0.50	0.00	0.00	1.00	0.50	0.00	0.00	0.00	0.00	0.00	0.32	1.00	1.00	1.00	0.00	
Cax1M226	0	0.37	0.11	0.70	0.00	0.00	0.00	0.00	0.00	0.00	0.25	0.22	1.00	0.50	0.00	0.00	1.00	0.50	0.00	0.00	0.00	0.00	1.00	1.00	1.00	**0.97**	1.00
Cax1P460	0	0.34	0.17	0.51	0.30	0.26	0.00	0.00	0.00	0.00	0.25	0.22	1.00	0.50	0.00	0.00	1.00	0.50	0.00	0.00	0.00	0.00	-0.12	0.82	0.84	**0.86**	0.86
LCY2-C-306	0	0.42	0.20	0.53	0.00	0.00	0.00	0.00	0.00	0.00	0.50	0.38	0.00	0.00	0.86	0.49	0.00	0.00	0.00	0.00	0.20	0.18	1.00	1.00	1.00	0.00	
LCY2-P-75	0	0.30	0.11	0.64	0.30	0.26	0.00	0.00	0.08	0.08	0.00	0.00	0.00	0.00	0.00	0.00	1.00	0.50	0.00	0.00	0.00	0.00	-0.08	0.76	0.78	**0.79**	0.80
LCYB-C-367	0	0.32	0.22	0.32	0.00	0.00	0.00	0.00	0.00	0.00	0.75	0.47	0.00	0.00	1.00	0.50	0.00	0.00	0.00	0.00	0.00	0.00	1.00	1.00	1.00	0.00	
LCYB-P-667	0	0.32	0.15	0.55	0.20	0.48	0.20	0.18	0.00	0.00	0.25	0.22	1.00	0.50	0.00	0.00	1.00	0.50	0.00	0.00	0.00	0.00	0.52	0.76	0.49	**0.50**	0.59
PKF-C-64	0	0.36	0.24	0.33	0.00	0.00	0.00	0.00	0.00	0.00	1.00	0.50	0.00	0.00	1.00	0.50	0.00	0.00	0.00	0.00	0.00	0.32	1.00	1.00	1.00	0.00	
PSY-C-426	0	0.32	0.21	0.36	0.00	0.00	0.00	0.00	0.00	0.00	0.75	0.47	0.00	0.00	1.00	0.50	0.00	0.00	0.00	0.00	0.00	0.00	1.00	1.00	1.00	0.00	
PSY-C-497	0	0.42	0.09	0.79	0.00	0.00	0.00	0.00	0.08	0.08	0.00	0.38	0.00	0.00	0.57	0.41	0.00	0.00	0.00	0.00	0.00	0.32	0.02	0.92	0.92	0.03	-0.01
TScC440	0	0.36	0.24	0.33	0.00	0.00	0.00	0.00	0.00	0.00	1.00	0.50	0.00	0.00	1.00	0.50	0.00	0.00	0.00	0.00	0.00	0.32	1.00	1.00	1.00	**0.00**	
