# Supplementary material for: SNP mining in C. clementina BAC end sequences; transferability in the Citrus genus (Rutaceae), phylogenetic inferences and perspectives for genetic mapping
Source: BMC Genomics. 2012 Jan 10;13:13. doi: 10.1186/1471-2164-13-13 (PMC3320530; doi:10.1186/1471-2164-13-13)
Supplement: Additional file 4 — Additional figures. This file contains two figures. Figure S1: Correlation between the contribution of SNPs loci to the first axis of PCA analysis (C. maxima and C. reticulata as active individuals) and the Fst values for C. maxima/C. reticulata differentiation. Figure S2: Co-distribution of LD between loci without null allele (WONA) pairs for Chandler × Clementine progeny and germplasm population [file 1471-2164-13-13-S4.PDF]

Additional file 4

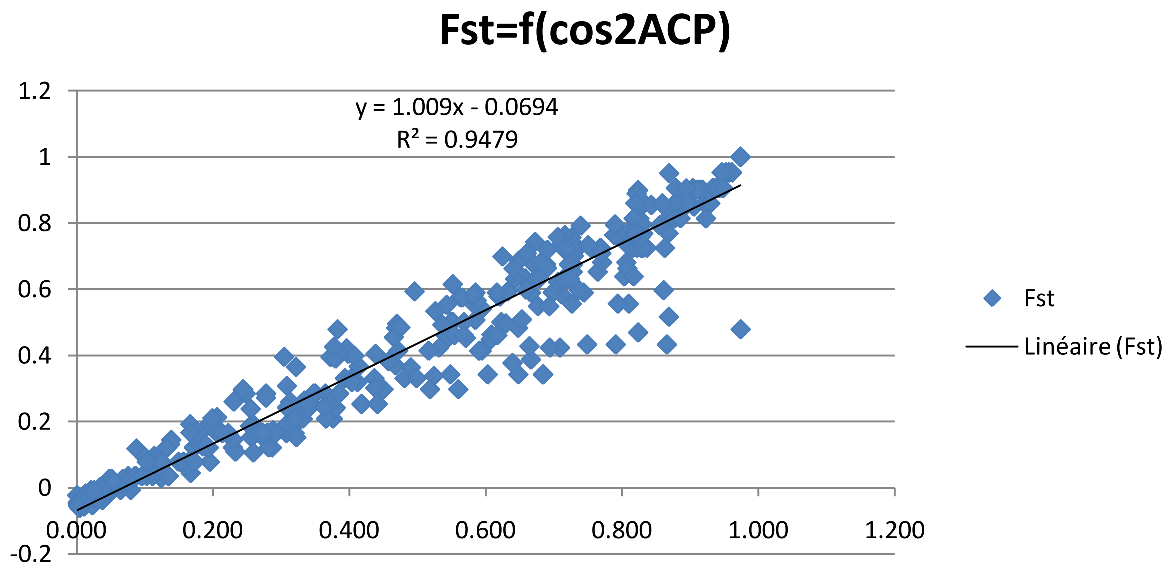

Figure A1: Correlation between the contribution of SNPs loci to the first axis of PCA analysis (*C. maxima* and *C. reticulata* as active individuals) and the Fst values for *C. maxima/C. reticulata* differentiation

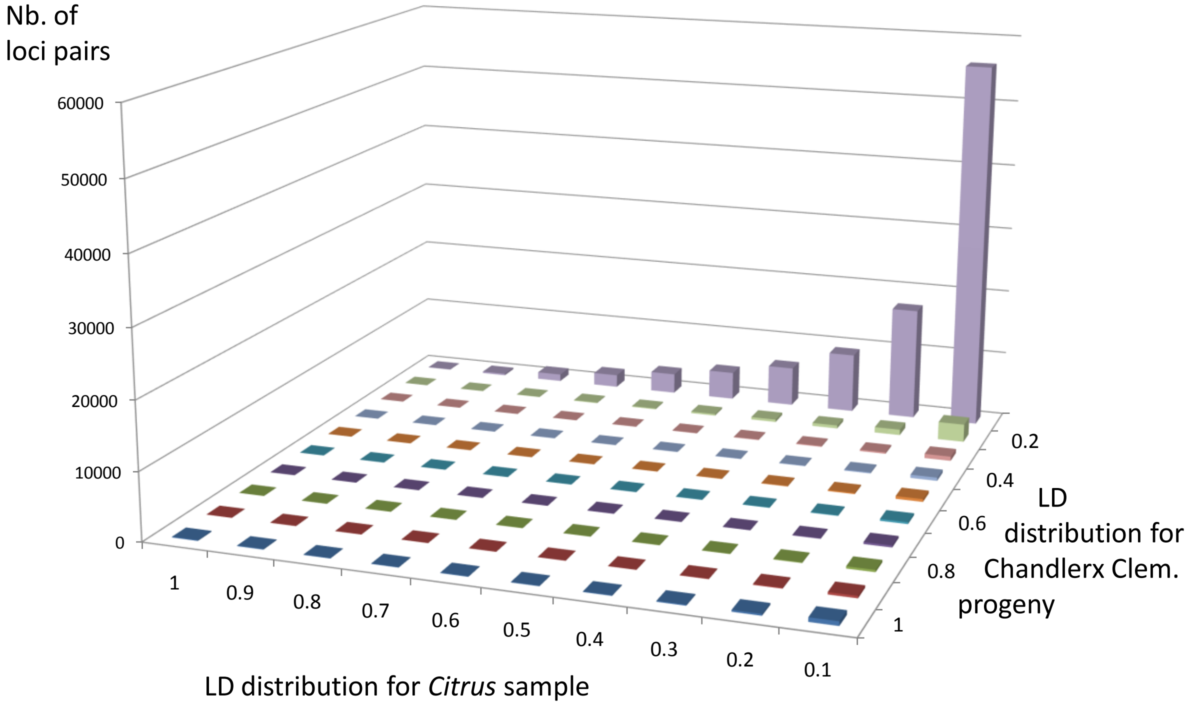

Figure A2: Co-distribution of Linkage Desequilibrium (LD estimated by r^2) between WONA locus pairs for Chandler x Clementine progeny and germplasm population.
