# Supplementary material for: SNP mining in C. clementina BAC end sequences; transferability in the Citrus genus (Rutaceae), phylogenetic inferences and perspectives for genetic mapping
Source: BMC Genomics. 2012 Jan 10;13:13. doi: 10.1186/1471-2164-13-13 (PMC3320530; doi:10.1186/1471-2164-13-13)
Supplement: Additional file 5 — Detailed diversity results for loci with null allele (WNA). This file contains main data on the results obtained with WNA loci. It includes (i) SNP heterozygosity and heterozygosity for null allele in Clementine, (ii) number of individual in heterozygosity (SNPs), homozygous for one SNP allele and homozygous for null allele in the whole population and within the different species, (iii) genotypic diversity in the whole population (PIC). [file 1471-2164-13-13-S5.PDF]

Additional File 5: Detailed observed polymorphism for WNA loci

	Clem	Het.	All individuals (54)				*C. maxima (10)*			*C. medica (5)*			*C. reticulata (12)*			*C. aurantifolia (4)*			*C. aurantium (2)*			*C. limon (7)*			*C. paradisi (2)*			*C.sinensis (4)*			*Papeda (5)*		
	SNP	X/0	hom	het	nul	PIC	hom	het	nul	hom	het	nul	hom	het	nul	hom	het	nul	hom	het	nul	hom	het	nul	hom	het	nul	hom	het	nul	hom	het	nul
**CiC0054-07**	0	1	23	0	31	0.49	0	0	10	0	0	5	11	0	1	0	0	4	2	0	0	7	0	0	0	0	2	0	0	4	1	0	4
**CiC0204-01**	1	0	23	8	23	0.70	0	0	10	0	0	5	5	7	0	2	0	2	2	0	0	7	0	0	2	0	0	4	0	0	1	0	4
**CiC0339-02**	1	0	38	13	3	0.56	9	0	1	5	0	0	8	3	1	3	1	0	0	2	0	6	1	0	2	0	0	0	4	0	3	1	1
**CiC0416-01**	0	1	13	0	41	0.37	1	0	9	0	0	5	4	0	8	0	0	4	0	0	2	1	0	6	2	0	0	4	0	0	0	0	5
**CiC0443-07**	0	1	15	0	39	0.40	1	0	9	0	0	5	4	0	8	2	0	2	2	0	0	1	0	6	0	0	2	0	0	4	3	0	2
**CiC0518-01**	1	0	30	2	22	0.54	0	0	10	0	0	5	11	1	0	2	0	2	2	0	0	7	0	0	2	0	0	4	0	0	1	0	4
**CiC0571-01**	1	0	31	17	6	0.67	6	0	4	5	0	0	7	5	0	2	2	0	0	2	0	4	3	0	2	0	0	0	4	0	3	0	2
**CiC0640-05**	1	0	30	2	22	0.61	0	0	10	1	0	4	10	1	1	2	0	2	2	0	0	6	0	1	2	0	0	4	0	0	3	0	2
**CiC0744-07**	1	0	27	3	24	0.65	1	0	9	0	0	5	10	2	0	1	0	3	2	0	0	6	0	1	2	0	0	4	0	0	0	0	5
**CiC0762-07**	0	0	28	0	26	0.50	0	0	10	0	0	5	12	0	0	1	0	3	2	0	0	4	0	3	0	0	2	4	0	0	2	0	3
**CiC0770-01**	0	1	9	0	45	0.28	0	0	10	0	0	5	3	0	9	2	0	2	0	0	2	0	0	7	0	0	2	0	0	4	3	0	2
**CiC0832-01**	1	0	39	4	11	0.48	1	0	9	5	0	0	10	2	0	4	0	0	2	0	0	6	1	0	2	0	0	4	0	0	3	0	2
**CiC0844-06**	0	0	33	0	21	0.48	0	0	10	0	0	5	12	0	0	2	0	2	2	0	0	7	0	0	2	0	0	4	0	0	1	0	4
**CiC0875-01**	1	0	29	4	21	0.69	1	0	9	5	0	0	8	1	3	3	0	1	0	0	2	5	2	0	0	0	2	4	0	0	1	0	4
**CiC0974-01**	0	0	13	0	41	0.37	0	0	10	0	0	5	5	0	7	1	0	3	0	0	2	0	0	7	2	0	0	4	0	0	0	0	5
**CiC1014-01**	0	1	25	0	29	0.50	0	0	10	0	0	5	12	0	0	2	0	2	2	0	0	7	0	0	0	0	2	0	0	4	1	0	4
**CiC1101-02**	0	0	32	0	22	0.48	0	0	10	0	0	5	12	0	0	2	0	2	2	0	0	7	0	0	2	0	0	4	0	0	2	0	3
**CiC1139-02**	1	0	24	7	23	0.62	1	1	8	0	0	5	9	3	0	0	0	4	2	0	0	4	2	1	2	0	0	4	0	0	0	0	5
**CiC1162-04**	0	1	12	0	42	0.35	0	0	10	0	0	5	4	0	8	0	0	4	0	0	2	1	0	6	2	0	0	4	0	0	0	0	5
**CiC1203-05**	1	0	26	9	19	0.72	0	0	10	0	0	5	5	7	0	4	0	0	2	0	0	5	1	1	2	0	0	4	0	0	4	0	1
**CiC1216-04**	1	0	23	4	27	0.58	0	0	10	0	0	5	9	3	0	1	0	3	2	0	0	4	0	3	0	0	2	4	0	0	1	0	4
**CiC1251-01**	1	0	33	14	7	0.67	9	0	1	0	1	4	5	7	0	4	0	0	2	0	0	7	0	0	2	0	0	0	4	0	2	1	2
**CiC1352-01**	1	0	39	10	5	0.44	7	1	2	5	0	0	8	4	0	4	0	0	2	0	0	7	0	0	2	0	0	0	4	0	2	0	3
**CiC1352-06**	1	0	35	10	9	0.52	10	0	0	0	1	4	8	4	0	2	0	2	2	0	0	7	0	0	2	0	0	0	4	0	2	0	3
**CiC1380-05**	1	0	45	5	4	0.35	7	0	3	5	0	0	12	0	0	4	0	0	2	0	0	7	0	0	2	0	0	0	4	0	4	0	1
**CiC1423-01**	0	0	31	0	23	0.49	0	0	10	0	0	5	12	0	0	1	0	3	2	0	0	6	0	1	2	0	0	4	0	0	1	0	4
**CiC1453-02**	1	0	19	29	6	0.63	5	1	4	0	4	1	2	10	0	3	1	0	1	1	0	4	2	1	0	2	0	0	4	0	2	3	0
**CiC1478-01**	1	0	25	6	23	0.67	0	0	10	0	0	5	7	5	0	1	0	3	2	0	0	6	0	1	2	0	0	4	0	0	2	0	3
**CiC1478-02**	1	0	31	3	20	0.57	0	0	10	0	0	5	11	1	0	3	0	1	2	0	0	6	0	1	2	0	0	4	0	0	2	1	2
**CiC1497-09**	0	1	15	0	39	0.40	0	0	10	0	0	5	8	0	4	0	0	4	0	0	2	4	0	3	0	0	2	0	0	4	2	0	3
**CiC1550-01**	1	0	25	7	22	0.68	0	0	10	0	0	5	6	6	0	1	0	3	2	0	0	7	0	0	2	0	0	4	0	0	2	0	3
**CiC1663-03**	0	0	31	0	23	0.49	10	0	0	0	0	5	2	0	10	3	0	1	2	0	0	2	0	5	2	0	0	4	0	0	3	0	2
**CiC1749-04**	1	0	45	1	8	0.40	5	0	5	5	0	0	12	0	0	4	0	0	2	0	0	7	0	0	0	0	2	4	0	0	4	0	1
**CiC1875-01**	1	0	45	7	2	0.45	9	0	1	5	0	0	10	1	1	3	1	0	2	0	0	3	4	0	2	0	0	4	0	0	5	0	0
**CiC1876-02**	1	0	33	14	7	0.56	7	0	3	5	0	0	4	7	1	4	0	0	0	2	0	2	3	2	2	0	0	4	0	0	4	0	1
**CiC1986-04**	0	1	28	0	26	0.50	0	0	10	0	0	5	12	0	0	2	0	2	2	0	0	6	0	1	0	0	2	4	0	0	1	0	4
**CiC2110-02**	1	0	22	10	22	0.67	0	0	10	1	0	4	3	9	0	1	0	3	2	0	0	7	0	0	2	0	0	4	0	0	1	0	4
**CiC2145-01**	0	0	46	0	8	0.25	10	0	0	5	0	0	4	0	8	4	0	0	2	0	0	7	0	0	2	0	0	4	0	0	5	0	0
**CiC2151-02**	0	0	20	0	34	0.47	10	0	0	0	0	5	0	0	12	1	0	3	2	0	0	0	0	7	2	0	0	4	0	0	0	0	5
**CiC2159-01**	1	0	34	9	11	0.53	0	0	10	5	0	0	4	8	0	4	0	0	2	0	0	7	0	0	2	0	0	4	0	0	4	0	1
**CiC2167-02**	1	0	20	9	25	0.67	0	0	10	0	0	5	8	4	0	1	0	3	2	0	0	5	0	2	2	0	0	0	4	0	1	0	4
**CiC2375-01**	0	0	27	1	26	0.52	10	0	0	0	0	5	3	0	9	4	0	0	2	0	0	0	1	6	2	0	0	0	0	4	4	0	1
**CiC2417-07**	0	1	5	0	49	0.17	0	0	10	0	0	5	3	0	9	0	0	4	0	0	2	1	0	6	0	0	2	0	0	4	0	0	5

	Clem	Het.	All individuals (54)				*C. maxima (10)*			*C. medica (5)*			*C. reticulata (12)*			*C. aurantifolia (4)*			*C. aurantium (2)*			*C. limon (7)*			*C. paradisi (2)*			*C.sinensis (4)*			*Papeda (5)*		
	SNP	X/0	hom	het	nul	PIC	hom	het	nul	hom	het	nul	hom	het	nul	hom	het	nul	hom	het	nul	hom	het	nul	hom	het	nul	hom	het	nul	hom	het	nul
**CiC2431-01**	1	0	38	9	7	0.71	10	0	0	1	1	3	7	5	0	2	0	2	0	2	0	7	0	0	2	0	0	4	0	0	3	0	2
**CiC2458-01**	0	0	44	0	10	0.46	9	0	1	4	0	1	12	0	0	1	0	3	2	0	0	5	0	2	2	0	0	4	0	0	2	0	3
**CiC2494-02**	0	1	24	0	30	0.49	0	0	10	0	0	5	8	0	4	1	0	3	2	0	0	5	0	2	2	0	0	4	0	0	1	0	4
**CiC2507-03**	1	0	28	16	10	0.74	0	0	10	5	0	0	7	5	0	3	1	0	2	0	0	3	4	0	2	0	0	0	4	0	5	0	0
**CiC2590-10**	0	1	12	0	42	0.36	1	0	9	0	0	5	4	0	8	0	0	4	2	0	0	0	0	7	0	0	2	4	0	0	0	0	5
**CiC2590-11**	1	0	37	17	0	0.60	9	1	0	0	5	0	6	6	0	3	1	0	2	0	0	5	2	0	2	0	0	4	0	0	4	1	0
**CiC2635-06**	1	0	37	6	11	0.57	0	0	10	5	0	0	9	3	0	4	0	0	2	0	0	5	2	0	2	0	0	4	0	0	4	0	1
**CiC2644-02**	1	0	20	11	23	0.71	0	0	10	0	0	5	6	6	0	1	0	3	2	0	0	6	0	1	2	0	0	0	4	0	2	0	3
**CiC2768-01**	1	0	25	7	22	0.71	0	0	10	0	0	5	6	6	0	2	0	2	2	0	0	6	0	1	2	0	0	4	0	0	2	0	3
**CiC2790-02**	1	0	35	10	9	0.64	10	0	0	1	3	1	6	6	0	1	0	3	2	0	0	6	0	1	2	0	0	4	0	0	2	0	3
**CiC2790-03**	1	0	25	6	23	0.69	0	0	10	2	0	3	7	5	0	1	0	3	2	0	0	5	0	2	2	0	0	4	0	0	1	0	4
**CiC2798-01**	0	0	33	0	21	0.48	5	0	5	0	0	5	12	0	0	1	0	3	0	0	2	4	0	3	2	0	0	4	0	0	4	0	1
**CiC2809-01**	1	0	12	14	28	0.64	0	0	10	0	0	5	3	9	0	1	0	3	0	0	2	3	0	4	2	0	0	0	4	0	3	0	2
**CiC2810-01**	1	0	40	8	6	0.44	10	0	0	0	0	5	6	6	0	4	0	0	2	0	0	6	1	0	2	0	0	4	0	0	4	0	1
**CiC2824-04**	1	0	38	15	1	0.66	9	1	0	5	0	0	8	4	0	3	1	0	0	2	0	4	3	0	2	0	0	4	0	0	3	1	1
**CiC2868-05**	0	0	33	0	21	0.48	0	0	10	0	0	5	12	0	0	2	0	2	2	0	0	7	0	0	2	0	0	4	0	0	1	0	4
**CiC2945-10**	0	1	28	0	26	0.50	0	0	10	0	0	5	11	0	1	1	0	3	2	0	0	6	0	1	2	0	0	4	0	0	1	0	4
**CiC3056-07**	0	1	24	5	25	0.63	9	1	0	1	0	4	8	1	3	1	0	3	0	2	0	3	1	3	0	0	2	0	0	4	0	0	5
**CiC3064-07**	0	1	21	0	33	0.48	0	0	10	0	0	5	11	0	1	0	0	4	2	0	0	4	0	3	0	0	2	0	0	4	1	0	4
**CiC3275-02**	1	0	35	4	15	0.50	0	0	10	5	0	0	10	2	0	3	1	0	2	0	0	7	0	0	0	0	2	4	0	0	2	0	3
**CiC3282-04**	0	0	25	0	29	0.50	10	0	0	0	0	5	0	0	12	1	0	3	2	0	0	3	0	4	2	0	0	4	0	0	0	0	5
**CiC3302-04**	1	0	42	12	0	0.53	10	0	0	5	0	0	10	2	0	3	1	0	2	0	0	5	2	0	0	2	0	0	4	0	5	0	0
**CiC3352-02**	1	0	23	3	28	0.56	1	0	9	0	0	5	10	2	0	1	0	3	2	0	0	3	0	4	0	0	2	4	0	0	1	0	4
**CiC3440-07**	1	0	29	15	10	0.72	2	0	8	5	0	0	4	8	0	4	0	0	2	0	0	3	4	0	0	0	2	4	0	0	5	0	0
**CiC3445-13**	0	0	34	0	20	0.47	0	0	10	0	0	5	12	0	0	2	0	2	2	0	0	7	0	0	2	0	0	4	0	0	2	0	3
**CiC3448-06**	1	0	40	6	8	0.47	1	1	8	5	0	0	8	4	0	4	0	0	2	0	0	7	0	0	2	0	0	4	0	0	5	0	0
**CiC3468-02**	0	0	29	24	1	0.66	3	7	0	1	4	0	11	1	0	2	1	1	0	2	0	3	4	0	0	2	0	4	0	0	3	2	0
**CiC3541-14**	0	1	11	0	43	0.32	0	0	10	0	0	5	5	0	7	0	0	4	0	0	2	2	0	5	0	0	2	0	0	4	3	0	2
**CiC3546-06**	1	0	38	3	13	0.44	0	0	10	5	0	0	11	1	0	2	1	1	2	0	0	7	0	0	0	0	2	4	0	0	5	0	0
**CiC3567-02**	1	0	28	4	22	0.64	1	0	9	0	0	5	9	3	0	1	0	3	2	0	0	6	0	1	2	0	0	4	0	0	2	0	3
**CiC3567-04**	1	0	27	4	23	0.64	0	0	10	0	0	5	9	3	0	1	0	3	2	0	0	7	0	0	2	0	0	4	0	0	1	0	4
**CiC3573-01**	1	0	27	14	13	0.66	0	0	10	5	0	0	6	6	0	3	1	0	2	0	0	5	2	0	2	0	0	0	4	0	2	0	3
**CiC3667-12**	0	0	32	0	22	0.48	0	0	10	0	0	5	12	0	0	2	0	2	2	0	0	7	0	0	2	0	0	4	0	0	1	0	4
**CiC3740-01**	1	0	42	2	10	0.39	7	0	3	0	0	5	11	1	0	4	0	0	2	0	0	7	0	0	0	0	2	4	0	0	5	0	0
**CiC3740-02**	1	0	37	14	3	0.60	6	1	3	5	0	0	8	4	0	4	0	0	2	0	0	4	3	0	2	0	0	0	4	0	4	1	0
**CiC3742-04**	1	0	32	14	8	0.73	8	1	1	0	0	5	7	5	0	4	0	0	0	2	0	6	0	1	2	0	0	0	4	0	3	1	1
**CiC3750-11**	0	0	39	0	15	0.54	0	0	10	4	0	1	12	0	0	2	0	2	2	0	0	7	0	0	2	0	0	4	0	0	3	0	2
**CiC3807-06**	0	1	20	0	34	0.47	0	0	10	0	0	5	11	0	1	1	0	3	0	0	2	2	0	5	2	0	0	3	0	1	0	0	5
**CiC3959-10**	1	0	33	21	0	0.64	7	3	0	5	0	0	10	2	0	2	2	0	2	0	0	0	7	0	2	0	0	0	4	0	5	0	0
**CiC4131-01**	1	0	39	7	8	0.47	10	0	0	0	1	4	8	4	0	2	0	2	2	0	0	6	1	0	2	0	0	4	0	0	3	0	2
**CiC4207-01**	0	0	35	0	19	0.46	0	0	10	3	0	2	12	0	0	3	0	1	2	0	0	6	0	1	2	0	0	4	0	0	1	0	4
**CiC4534-01**	1	0	40	13	1	0.58	9	0	1	5	0	0	11	1	0	3	1	0	0	2	0	5	2	0	2	0	0	0	4	0	5	0	0
**CiC4539-09**	0	0	34	0	20	0.55	0	0	10	4	0	1	11	0	1	2	0	2	2	0	0	7	0	0	2	0	0	4	0	0	1	0	4
**CiC4643-03**	1	0	22	11	21	0.71	2	0	8	0	0	5	6	6	0	1	0	3	2	0	0	6	0	1	2	0	0	0	4	0	1	0	4
**CiC4743-02**	1	0	35	12	7	0.62	7	0	3	0	1	4	5	7	0	4	0	0	2	0	0	6	1	0	0	2	0	4	0	0	5	0	0
**CiC4877-04**	1	0	35	11	8	0.58	4	4	2	0	0	5	10	2	0	4	0	0	2	0	0	7	0	0	2	0	0	0	4	0	5	0	0

	Clem	Het.	All individuals (54)				*C. maxima (10)*			*C. medica (5)*			*C. reticulata (12)*			*C. aurantifolia (4)*			*C. aurantium (2)*			*C. limon (7)*			*C. paradisi (2)*			*C.sinensis (4)*			*Papeda (5)*		
	SNP	X/0	hom	het	nul	PIC	hom	het	nul	hom	het	nul	hom	het	nul	hom	het	nul	hom	het	nul	hom	het	nul	hom	het	nul	hom	het	nul	hom	het	nul
**CiC4954-02**	1	0	35	13	6	0.51	10	0	0	0	0	5	7	5	0	3	0	1	2	0	0	6	1	0	0	2	0	0	4	0	5	0	0
**CiC5078-07**	1	0	28	17	9	0.73	10	0	0	0	0	5	4	6	2	3	0	1	0	2	0	6	1	0	0	2	0	0	4	0	3	1	1
**CiC5173-01**	1	0	40	13	1	0.39	8	1	1	5	0	0	7	5	0	4	0	0	2	0	0	7	0	0	0	2	0	0	4	0	5	0	0
**CiC5274-01**	1	0	23	4	27	0.58	0	0	10	0	0	5	9	3	0	1	0	3	2	0	0	4	0	3	0	0	2	4	0	0	1	0	4
**CiC5283-06**	0	0	33	0	21	0.48	0	0	10	0	0	5	12	0	0	2	0	2	2	0	0	7	0	0	2	0	0	4	0	0	1	0	4
**CiC5439-02**	0	1	37	0	17	0.43	1	0	9	5	0	0	12	0	0	2	0	2	2	0	0	7	0	0	0	0	2	4	0	0	2	0	3
**CiC5485-05**	0	1	44	0	10	0.41	3	0	7	5	0	0	12	0	0	4	0	0	2	0	0	7	0	0	0	0	2	4	0	0	4	0	1
**CiC5535-01**	1	0	24	6	24	0.64	0	0	10	0	0	5	7	5	0	1	0	3	2	0	0	6	0	1	0	0	2	4	0	0	2	0	3
**CiC5589-03**	1	0	46	8	0	0.37	7	3	0	5	0	0	12	0	0	4	0	0	2	0	0	7	0	0	2	0	0	0	4	0	5	0	0
**CiC5609-02**	1	0	21	5	28	0.63	0	0	10	0	0	5	8	4	0	0	0	4	2	0	0	4	0	3	0	0	2	4	0	0	1	0	4
**CiC5650-01**	1	0	23	10	21	0.67	0	0	10	1	0	4	3	9	0	1	0	3	2	0	0	7	0	0	2	0	0	4	0	0	2	0	3
**CiC5685-01**	0	0	31	17	6	0.70	10	0	0	0	0	5	5	7	0	3	1	0	0	2	0	7	0	0	0	2	0	0	4	0	3	1	1
**CiC5737-06**	1	0	37	11	6	0.66	7	3	0	0	1	4	12	0	0	3	0	1	0	2	0	7	0	0	2	0	0	0	4	0	4	0	1
**CiC5766-06**	1	0	29	5	20	0.59	2	0	8	0	0	5	8	4	0	1	0	3	2	0	0	6	0	1	2	0	0	4	0	0	2	0	3
**CiC5791-06**	0	0	16	0	38	0.42	0	0	10	0	0	5	8	0	4	1	0	3	0	0	2	0	0	7	2	0	0	4	0	0	0	0	5
**CiC5805-01**	1	0	23	8	23	0.62	0	0	10	0	0	5	5	7	0	2	0	2	2	0	0	7	0	0	2	0	0	4	0	0	1	0	4
**CiC5810-01**	0	0	29	19	6	0.72	6	4	0	0	0	5	6	6	0	4	0	0	0	2	0	7	0	0	0	2	0	0	4	0	3	1	1
**CiC5810-04**	0	0	36	7	11	0.68	5	1	4	0	2	3	8	4	0	4	0	0	2	0	0	7	0	0	2	0	0	4	0	0	3	0	2
**CiC5833-02**	1	0	30	18	6	0.72	9	1	0	0	0	5	7	5	0	1	3	0	0	2	0	7	0	0	2	0	0	0	4	0	2	2	1
**CiC5843-03**	1	0	21	11	22	0.71	0	1	9	0	0	5	4	8	0	3	0	1	2	0	0	5	0	2	0	0	2	4	0	0	3	1	1
**CiC5874-05**	1	0	40	12	2	0.56	10	0	0	5	0	0	6	6	0	4	0	0	0	2	0	4	2	1	2	0	0	4	0	0	5	0	0
**CiC6014-14**	0	1	38	14	2	0.51	6	4	0	5	0	0	3	7	2	4	0	0	0	2	0	7	0	0	2	0	0	4	0	0	4	1	0
**CiC6022-03**	0	1	20	0	34	0.47	0	0	10	0	0	5	7	0	5	0	0	4	2	0	0	3	0	4	2	0	0	4	0	0	0	0	5
**CiC6106-02**	0	0	50	4	0	0.55	10	0	0	5	0	0	12	0	0	4	0	0	2	0	0	6	1	0	0	2	0	4	0	0	4	1	0
**CiC6278-01**	1	0	33	4	17	0.52	8	1	1	0	0	5	8	1	3	2	0	2	2	0	0	4	1	2	2	0	0	4	0	0	3	0	2
**CiC6294-03**	1	0	40	14	0	0.61	9	1	0	5	0	0	9	3	0	3	1	0	0	2	0	6	1	0	2	0	0	0	4	0	5	0	0
**CiC6314-03**	1	0	44	10	0	0.50	10	0	0	5	0	0	8	4	0	4	0	0	2	0	0	3	4	0	2	0	0	4	0	0	4	1	0
**Mid Nb / loc**			64.2	14	37.8		41.1	4.3	70.6	36.8	4.8	74.4	76	27.2	12.8	63.8	5.25	47	86.5	17.5	12	84	10	22	80	10	26	77.8	28	10.3	56.8	4.2	55
**Freq**			0.55	0.12	0.33		0.35	0.04	0.61	0.32	0.04	0.64	0.66	0.23	0.11	0.55	0.05	0.41	0.75	0.15	0.1	0.72	0.09	0.19	0.69	0.09	0.22	0.67	0.24	0.09	0.49	0.04	0.47
