# Supplementary material for: SNP mining in C. clementina BAC end sequences; transferability in the Citrus genus (Rutaceae), phylogenetic inferences and perspectives for genetic mapping
Source: BMC Genomics. 2012 Jan 10;13:13. doi: 10.1186/1471-2164-13-13 (PMC3320530; doi:10.1186/1471-2164-13-13)
Supplement: Additional file 6 — List of germplasm analyzed. This file contains the list of citrus germplasm accession analyzed. It includes the gerplasm bank, the accession number, the varietal group, the common name, the Latin name according Swingle and Reece and Tanaka classifications and the use for SNP genotyping validation by sequencing. [file 1471-2164-13-13-S6.PDF]

Additional file 6: List of germplasm analysed

Number	Varietal group	Common name	Swingle and Reece (1967)	Tanaka (1961)	Germplasm Bank	Accession number	SNP validation
1	Pummelo	Azimboa	*C. maxima*	*C. maxima*	IVIA	420	
2	Pummelo	Chandler	*C. maxima*	*C. maxima*	IVIA	207	1
3	Pummelo	DaXanh	*C. maxima*	*C. maxima*	IVIA	589	
4	Pummelo	Deep Red	*C. maxima*	*C. maxima*	IVIA	277	
5	Pummelo	Flores	*C. maxima*	*C. maxima*	INRA/CIRAD	ICVN 0100673	
6	Pummelo	Nam Roi	*C. maxima*	*C. maxima*	IVIA	590	1
7	Pummelo	Pink	*C. maxima*	*C. maxima*	IVIA	275	1
8	Pummelo	Sans pepins	*C. maxima*	*C. maxima*	INRA/CIRAD	ICVN 0110317	1
9	Pummelo	Tahiti	*C. maxima*	*C. maxima*	INRA/CIRAD	ICVN 0100727	1
10	Pummelo	Timor	*C. maxima*	*C. maxima*	INRA/CIRAD	ICVN 0100707	
11	Citron	Arizona	*C. medica*	*C. medica*	IVIA	169	1
12	Citron	Corsican	*C. medica*	*C. medica*	IVIA	567	1
13	Citron	Damas	*C. medica*	*C. medica*	INRA/CIRAD	ICVN 0100837	
14	Citron	Diamante	*C. medica*	*C. medica*	IVIA	560	1
15	Citron	Buddha's hand	*C. medica*	*C. medica*	IVIA	202	1
16	Citron	Humpang	*C. medica*	*C. medica*	INRA/CIRAD	ICVN 0100722	
17	Citron	Rhobs el Arsa	*C. medica*	*C. medica*	INRA/CIRAD	ICVN 0110244	
18	Mandarin	Mediterranean	*C. reticulata*	*C. deliciosa*	IVIA	44	1
19	Mandarin	Citrus depressa	*C. reticulata*	*C. depressa*	IVIA	238	
20	Mandarin	Fuzhu	*C. reticulata*	*C. erythrosa*	IVIA	571	
21	Mandarin	King	*C. reticulata*	*C. nobilis*	IVIA	477	1
22	Mandarin	Cleopatra	*C. reticulata*	*C. reshni*	IVIA	385	1
23	Mandarin	Anana	*C. reticulata*	*C. reticulata*	IVIA	390	
24	Mandarin	Imperial Australia	*C. reticulata*	*C. reticulata*	IVIA	576	
25	Mandarin	Ponkan	*C. reticulata*	*C. reticulata*	IVIA	482	1
26	Mandarin	Sun Chu Sha	*C. reticulata*	*C. reticulata*	IVIA	483	
27	Mandarin	Sunki	*C. reticulata*	*C. sunki*	IVIA	239	1
28	Mandarin	Dancy	*C. reticulata*	*C. tangerina*	IVIA	434	1
29	Mandarin	Clauselina	*C. reticulata*	*C. unshiu*	IVIA	19	1
30	Papeda	Nasnaran	*C. reti. Hyb.*	*C. amblycarpa*	IVIA	478	
31	Papeda	Ichang	*C. ichangensis*	*C. ichangensis*	IVIA	235	
32	Papeda	Indica	*C. indica*	*C. indica*	IVIA	550	
33	Papeda	Micrantha	*C. micrantha*	*C. micrantha*	IVIA	Micrantha	1
34	Papeda	Combava	*C. hystrix*	*C. hystrix*	IVIA	178	
35	Lime	Mexican	*C. aurantifolia*	*C. aurantifolia*	IVIA	164	1
36	Lime	Bergamot Calabria	*C. aurantifolia*	*C. bergamia*	IVIA	254	
37	Lime	Palestine Sweet	*C. aurantifolia*	*C. limettioides*	IVIA	305	
38	Lime	Alemow	*C. aurantifolia*	*C. macrophylla*	IVIA	288	
39	Sour orange	Bouquet de fleurs	*C. aurantium*	*C. aurantium*	IVIA	139	1
40	Sour orange	Sevillano	*C. aurantium*	*C. aurantium*	IVIA	117	1
41	Clementine	Nules	*C. reticulata*	*C. clementina*	IVIA	22	1
42	Lemon	Rough lemon	*C. lemon*	*C. jambhiri*	IVIA	333	
43	Lemon	Limonette Marrakech	*C. lemon*	*C. limetta*	IVIA	484	
44	Lemon	Eureka	*C. lemon*	*C. lemon*	IVIA	297	1
45	Lemon	Lisbon Frost	*C. lemon*	*C. lemon*	IVIA	219	
46	Lemon	Rangpur lime	*C. lemon*	*C. limonia*	IVIA	334	
47	Lemon	Meyer	*C. lemon*	*C. meyeri*	IVIA	145	
48	Lemon	Volkamer lemon	*C. lemon*	*C. limonia*	IVIA	432	
49	Grapefruit	Marsh	*C. paradisi*	*C. paradisi*	IVIA	176	1
50	Grapefruit	Star ruby	*C. paradisi*	*C. paradisi*	IVIA	197	
51	Sweet orange	Moro	*C. sinensis*	*C. sinensis*	INRA/CIRAD	ICVN 0100301	
52	Sweet orange	Sanguinelli	*C. sinensis*	*C. sinensis*	IVIA	34	
53	Sweet orange	Valencia late delta	*C. sinensis*	*C. sinensis*	IVIA	363	1
54	Sweet orange	Washington Frost	*C. sinensis*	*C. sinensis*	IVIA	222	
