# Supplementary material for: SNP mining in C. clementina BAC end sequences; transferability in the Citrus genus (Rutaceae), phylogenetic inferences and perspectives for genetic mapping
Source: BMC Genomics. 2012 Jan 10;13:13. doi: 10.1186/1471-2164-13-13 (PMC3320530; doi:10.1186/1471-2164-13-13)
Supplement: Additional file 7 — Primers for analyzing the origin of unexpected genotyping in the GoldenGate array. this file contains the main information on the primers used for analyzing the origin of unexpected genotyping in the GoldenGate array. It includes the locus name, the abnormality type, the primer sequences, annealing temperature and amplicon size. [file 1471-2164-13-13-S7.PDF]

Additional file 7: Primers for analyzing the origin of unexpected genotyping in the GoldenGate array.

SNP locus	Abnormality type	Primers	Ta	product length
**CiC0002-01**	Clem homozygosity	F: GCTCATCCGGAATTGGACTA<br>R: ACCCTTCGGGAGACTTGAAT	60	249
**CiC0049-02**	Clem homozygosity	F: AGAGGACTGCCTGGTTGCTA<br>R: CAATTGATCACGCCGTAAAA	60	292
**CiC0063-12**	Clem homozygosity	F: TCTTCCTTAGGGATGATGCAA<br>R: GCTCTTGCTTTAGTCGTTTCTG	58	222
**CiC0074-09**	Clem homozygosity	F: GGGGAAGGTGAAACTCTTAGG<br>R: AAATTGAATTAGTTAGGGTTGTGGA	58	379
**CiC0091-09**	Clem homozygosity	F: TGGCCGTGAGTTCTCTCAAT<br>R: CGTTGATTAACGCCCGTAGT	60	370
**CiC0113-01**	Clem homozygosity	F: TCTGGGATCAGACCAGACAA<br>R: CTTCCTCAGTGCCTCAACAA	58	399
**CiC2553-01**	Clem homozygosity	F: GGGCTCCTCAAGTGGTGATA<br>R: TCTTTCTTCCCCTTCATCCA	60	293
**CiC4252-10**	fixed heterozygosity	F: AGAAATTCCTCGACGTTCCA<br>R: GGTCGGAGACTGGGTATTGA	60	318
**CiC3064-07**	null allele	F: CATCAGCTGCCACGAAATTA<br>R: GTGGTCATGATGGCTGATTG	60	471
**CiC3275-02**	null allele	F: GGATTTGGATGGAAATGTGG<br>R: GACTCCAGGGGTTGAAACAA	60	478
**CiC2151-02**	null allele	TCGAAGTGAGTTGGCAATTTT<br>TCATGCAAAAATGTGGTGGT	60	336
